# Characterization of Accessible Chromatin Regions in Cattle Rumen Epithelial Tissue during Weaning

**DOI:** 10.3390/genes13030535

**Published:** 2022-03-18

**Authors:** Clarissa Boschiero, Yahui Gao, Ransom L. Baldwin, Li Ma, George E. Liu, Cong-Jun Li

**Affiliations:** 1 Animal Genomics and Improvement Laboratory, BARC, Agricultural Research Service, United States Department of Agriculture, Beltsville, MD 20705, USA; clarissa.boschiero@usda.gov (C.B.); gyhalvin@gmail.com (Y.G.); ransom.baldwin@usda.gov (R.L.B.VI); george.liu@usda.gov (G.E.L.); 2 Department of Animal and Avian Sciences, University of Maryland, College Park, MD 20742, USA; lima@umd.edu

**Keywords:** ATAC-seq, cattle, epithelial tissue, open chromatin, rumen development, weaning

## Abstract

Weaning in ruminants is characterized by the transition from a milk-based diet to a solid diet, which drives a critical gastrointestinal tract transformation. Understanding the regulatory control of this transformation during weaning can help to identify strategies to improve rumen health. This study aimed to identify regions of accessible chromatin in rumen epithelial tissue in pre- and post-weaning calves and investigate differentially accessible regions (DARs) to uncover regulatory elements in cattle rumen development using the ATAC-seq approach. A total of 126,071 peaks were identified, covering 1.15% of the cattle genome. From these accessible regions, 2766 DARs were discovered. Gene ontology enrichment resulted in GO terms related to the cell adhesion, anchoring junction, growth, cell migration, motility, and morphogenesis. In addition, putative regulatory canonical pathways were identified (TGFβ, integrin-linked kinase, integrin signaling, and regulation of the epithelial–mesenchymal transition). Canonical pathways integrated with co-expression results showed that TGFβ and ILK signaling pathways play essential roles in rumen development through the regulation of cellular adhesions. In this study, DARs during weaning were identified, revealing enhancers, transcription factors, and candidate target genes that represent potential biomarkers for the bovine rumen development, which will serve as a molecular tool for rumen development studies.

## 1. Introduction

The rumen is a complex organ that hosts a complex microbial community that facilitates the digestion of lignocellulose biomass. This microbial population contains prokaryotic and eukaryotic microorganisms that convert non-protein nitrogen into microbial proteins [1]. Moreover, this bacteria population also produces volatile fatty acids (VFAs), including acetic acid (~70%), propionic acid (~20%), and butyric acid (~10%), which serve as the primarily absorbed energy substrates for ruminants. Further, rumen has a role not only in cattle nutrition but also in cattle health as a defensive barrier to harmful substances [2]. Thus, the study of rumen biology and its development is fundamental to improving livestock management, nutrition, performance, and health [3].

Rumen development begins upon the establishment of a viable microbiota and ruminal fermentation after the intake of solid feeds [3]. The weaning process in ruminants is characterized by the transition from a milk-based diet to a solid diet. It is one of the essential gastrointestinal tract transformations resulting in structural and physiological changes [4]. In natural conditions, the weaning of calves occurs at the age of 6–9 months, but early weaning in the dairy industry is used to enhance herd management and improve health and productivity [5]. One obvious and important structural change during weaning is that rumen capacity increases from 30% to 70% of the gastrointestinal tract size [6]. In addition, the height and width of the rumen papillae increase during weaning, increasing the surface area for the absorption of VFAs [4].

It is of great interest for the dairy industry that optimal weaning strategies be identified to promote rumen development to ensure calf health. One way of improving the understanding of the process driving the rumen’s response to weaning is the identification of the genetic mechanisms in the rumen tissue that are affected by weaning. A previous study evaluated the gene expression in the bovine rumen during the introduction of solid feed and weaning, and two genes (*TRIM40* and *BPIFA1*) were differentially expressed post-weaning in the rumen epithelium [7]. Another study evaluated the expression of 11 selected genes in the rumen, reticulum, omasum, and abomasum in Japanese male calves, and three genes (*HMGCS2, AKR1C1*, and *FABP3*) were induced by the weaning [8]. Genome-wide studies have also been reported, including a transcriptome analysis of rumen papillae of weaned calves that identified 871 differentially expressed genes in the weaned group [9], and recently, a single-cell transcriptomic study identified distinct gene clusters in dairy cattle ruminal epithelial cells after weaning [10].

ATAC-seq (assay of transposase accessible chromatin sequencing) is a sensitive and fast method used to identify chromatin accessibility across the genome [11]. ATAC-seq enables the identification of chromatin accessibility and the examination of regulatory elements including promoters, enhancers, and insulators. The use of ATAC-seq for rumen epithelial tissue to generate a comprehensive landscape of chromatin events will advance our knowledge of the regulatory element mechanisms in play during rumen development. Few studies have used ATAC-seq in cattle [12,13,14]. Recent studies using ATAC-seq reported open chromatin regions for bronchial lymph nodes of dairy calves [12]; liver, muscle, and hypothalamus of indicine cattle [13]; and muscle tissue in adults and embryos from Qinchuan cattle [14]; however, none of them evaluated rumen, especially in the critical weaning period. The main objectives of this study were to identify and characterize regions of accessible chromatin in pre- and post-weaning calves from rumen epithelial tissue and to identify differentially accessible chromatin regions (DARs) using the ATAC-seq approach to elucidate genetic regulatory elements during the weaning in cattle.

## 2. Materials and Methods

### 2.1. Rumen Epithelial Tissue Collection

The Beltsville Area Animal Care approved the animal care and tissue isolation work (Committee Protocol Number 07-025). Animals and tissue sample collection were described in a previous report [15]. Four Holstein bull calves were utilized: two (before weaning, BW) were fed with milk replacer only (MRO—Cornerstone 22:20, Purina Mills, St. Louis, MO, USA; 22.0% crude protein, 20.0% crude fat, 0.15% crude fiber, 0.75% to 1.25% Ca, 0.70% P, 66,000 IU/kg vitamin A, 11,000 IU/kg vitamin D3, and 220 IU/kg vitamin E) for two weeks; while the other two (after weaning, AW) were fed with MRO for six weeks, followed by a combination of milk replacer and grain-based commercial calf starter for four weeks. Calves were euthanized by captive bolt followed by exsanguination at day 14 or day 70 to represent development at the two stages of weaning on a grain concentrate diet. Rumen epithelial tissue was collected from the anterior portion of the ventral sac of the rumen beneath the reticulum and below the rumen fluid layer at slaughter. The epithelial layer of the rumen tissue was separated manually from the muscular layer. After being rinsed in tap water to remove residual feed particles, samples were further rinsed in ice-cold saline and snap frozen in liquid nitrogen before being moved to −80 °C for future use [7]. Two biological replicates for each condition were obtained. Then, samples were fixed in RNAlater RNA stabilization solution (Life Technologies, Grand Island, NY, USA) according to the manufacturer’s instructions and stored at −80 °C.

### 2.2. ATAC Sequencing

ATAC-seq of four samples (two BW and two AW) was performed by Active Motif, Inc. (Carlsbad, CA, USA). Before the sequencing, a 50 mL digestion solution (1% trypsin and 1.15 mmol CaCl_2_ in phosphate-buffered saline) was added to the rumen epithelial tissue and incubated at 37 °C for 15 min to dissociate the cells. Rumen epithelial fragments underwent five to six cycles of digestion with trypsin solution. The first two rounds of digestion solution were discarded, and the third, fourth, and fifth rounds were collected and combined. The cell samples were thawed in a 37 °C water bath, pelleted, and washed with cold PBS. The resulted cells were tagmented as previously described [11,16]. In brief, cell pellets were resuspended with lysis buffer, pelleted, and tagmented using the enzyme and buffer provided in the Nextera Library Prep Kit (Illumina, San Diego, CA, USA). The MinElute PCR purification kit (QIAGEN, Germantown, MD, USA) was used to purify tagmented DNA, and then, the DNA was amplified with 10 cycles of PCR and purified using Agencourt AMPure SPRI beads (Beckman Coulter, Pasadena, CA, USA). The resulting material was quantified using the KAPA Library Quantification Kit for Illumina platforms (KAPA Biosystems, Wilmington, MA, USA). The DNA libraries were sequenced (2 × 75 bp) on a HiSeq 2500 platform (Illumina, San Diego, CA, USA). 

### 2.3. Data Processing and Mapping

First, sequence reads were examined for quality using FastQC v.0.11.9 (https://www.bioinformatics.babraham.ac.uk/projects/fastqc/, accessed on 18 May 2021), and then, adapters and reads with low quality (<20) were removed. Reads were then aligned to the ARS-USD1.2 cattle reference genome assembly [17] using BWA v.0.7.17 with default settings [18]. Unmapped reads, reads mapped to multiple locations, reads with a mapping quality (MQ) < 10, and reads located on the mitochondrial chromosome were removed using SAMtools v.1.9 [19]. Duplicate reads were removed with Picard v.2.22.3 (https://broadinstitute.github.io/picard/, accessed on 25 May 2021). The fragment size distribution was obtained with SAMtools v.1.9 [19].

### 2.4. Peak Calling

Peaks were identified for each sample with MACS2 v.2.2.7.1 [20], using the BAMPE parameter (FDR < 0.05). Peaks located on chromosome X or unplaced ones were removed to reduce bias. The fraction of all mapped reads in enriched peaks (FRiP) was obtained for each sample. BEDtools v.2.25.0 [21] Jaccard was used for pairwise comparisons of all samples to obtain the similarity score between samples and the number of peak intersections, representing the ratio of the number of base pairs in the intersection to the number of base pairs in the union. In addition, the BEDtools v.2.25.0 [21] intersect option was used to merge replicate peaks, and the intersect −v option was used to obtain the specific number of peaks for each condition. The DiffBind Bioconductor package [22] was used to construct the correlation heatmap using peak information from each sample.

### 2.5. Identification of Differentially Accessible Regions

DiffReps v.1.55.6 [23] was used to identify the DARs of the after weaning vs. before weaning comparison. Sample BW2 was not considered due to its low quality. BAM files were used as an input with a defined window of 200 bp and G-tested (*p*-value < 0.05). The significant differentially accessible regions were defined with an FDR value < 0.01 and log2 fold change ≤ −1 or log2 fold change ≥ 1. Then, the significant DARs were mapped against the identified peaks to obtain DARs that overlapped with MACS2 results. DiffReps does not utilize peaks generated by MACS2. A similar approach was performed before in mice [24]. The identified peaks from weaning (BW1, AW1/AW2) were merged into a single file by BEDtools v.2.25.0 [21] with a merge option that generated a list of non-overlapping peaks. Then, the significant DARs were compared and overlapped against the merged peak list using BEDtools v.2.25.0 [21] with the intersect function. The DARs that coincided with MACS2 peaks in at least one replicate were further analyzed (induced and repressed DARs together).

### 2.6. Annotation of Differentially Accessible Regions

A total of 2766 unique DARs were annotated with the annotatePeak function from the R/Bioconductor ChIPseeker package [25]. Promoter regions were defined as ±2 kb from the TSS. In addition, the annotatePeak function from the ChIPseeker package [25] was used to generate the plot of the distribution of transcription factor-binding loci relative to the TSS of the DARs. The distance from the regions (binding sites) to the TSS of the nearest gene was calculated using annotatePeak. 

In addition, weaning DARs were compared with 15 chromatin state segments previously identified by our group in the cattle [15] using the ChromHMM tool [26]. First, all segment coordinates were converted to the ARS-USD1.2 cattle reference genome assembly [17] using liftOver (https://genome.ucsc.edu/cgi-bin/hgLiftOver, accessed on 9 November 2021) with the default parameters (minimum ratio of bases that must remap = 0.95). The converted coordinates were compared with weaning DARs using BEDtools v.2.25.0 [21] with intersect function. Then, the enrichment fold of each state was obtained using ChromHMM [26]. 

### 2.7. Gene Ontology and Pathway Analysis of Differentially Accessible Regions

Gene ontology (GO) enrichment analyses were performed using GREAT v.4.0.4 [27] with default parameters using the unique weaning DARs. Before the analysis, all coordinates of each DAR were converted to human hg38 using liftOver (https://genome.ucsc.edu/cgi-bin/hgLiftOver, accessed on 7 October 2021) (minMatch = 0.1). Only results from the hypergeometric test were considered (*p*-value adjusted < 0.05). The GO-Figure tool was used to plot a summary of the GO-enriched terms using semantic similarity (https://gitlab.com/evogenlab/GO-Figure, accessed on 9 October 2021). QIAGEN Ingenuity Pathway Analysis (IPA) v.68752261 [28] was used with default parameters to find signaling and metabolic pathways from 1959 unique genes from weaning DARs, including canonical pathways (*p*-value < 0.01), upstream regulators (*p*-value of overlap < 0.01), and molecular networks (network score > 20). 

### 2.8. Motif Enrichment of Differentially Accessible Regions

To obtain enriched motifs and predict target genes, i-*cis*Target v.6.0 [29] was used. Before the analysis, all coordinates from DARs were converted to human hg38 using liftOver (https://genome.ucsc.edu/cgi-bin/hgLiftOver, accessed on 5 October 2021) with the default parameters (minMatch = 0.1). Then, the converted hg38 coordinates were converted again to human hg19 (minMatch = 0.95). A total of 2368 converted coordinates were used as an input. All available databases were selected for the analysis, including 24,453 position weight matrices (PWM), 1331 TF binding sites, 2450 histone modifications, and 655 DHS and FAIRE.

### 2.9. Gene Co-Expression Network Analysis

To investigate gene co-expression and regulatory networks and compare with the regulatory elements identified, previously described RNA-seq data from weaning (six samples with three biological replicates) were utilized [15]. The RNA-seq data are available at the NCBI SRA database (BioProject ID: PRJNA658627). RNA-seq clean reads (Q > 20) were aligned to the ARS-USD1.2 cattle genome assembly [17] with STAR v.2.7 [30], and gene expressions were obtained using Cufflinks v.2.2.1 [31]. The FPKM value of each gene was utilized for the weighted correlation network analysis with WGCNA v.1.70-3 [32]. The topological overlap matrix (TOM) was constructed with a soft-thresholding power of 9, followed by a dissimilarity calculation (1-TOM). Then, the modules were identified using the dynamic tree cut method (minimum size of 20). Modules whose expression profiles were very similar were merged by calculating the dissimilarity of module eigengenes. For module grouping, a threshold of 0.2 was used and corresponded to a correlation of 0.8. The network of genes from selected pathways and co-expressed genes was constructed using VisANT v.5 [33]. 

## 3. Results 

### 3.1. Data Quality and Peak Calling

A total of 318,737,324 paired-end reads were generated for all four samples with an average of 79,684,331, and the sample BW1 presented the lowest number of reads (Table 1). Figure S1 shows the fragment size distribution of the reads for each sample, and all the samples exhibited the expected fragment sizes with abundant nucleosome-free fragments (<100 bp) and mononucleosomal spanning fragments. Approximately 95% of the reads were aligned to the ARS-USD1.2 cattle reference genome assembly [17] with 304,253,083 reads mapped and an average of 76,063,271 reads (Table 1). On average, 6.63% of the reads were mapped to the mitochondrial genome; 1.66% were duplicated, and 29.60% had a mapping quality < 10 (Table 1). A total of 197,491,122 clean paired-end reads were produced (Table 1).

The peaks were identified in the individual samples by the MACS2 [20]. A total of 197,491,122 clean reads were used for peak calling, generating 274,933 peaks (FDR < 0.05) for all samples with an average of 68,733 peaks, and an average peak length of 211 (Table 2). The chromosomal distribution of peaks was similar among all samples, with more peaks located on chromosomes 1–3, 5, 11, and 19 with an average of >3800 peaks for each chromosome, except for BW2 (Figure S2). There were more peaks identified in the AW (152,330) than in the BW samples (122,603) (Table 2). 

BW2 had the lowest number of peaks, with only 27,640 peaks. Because of the low number of peaks identified in this sample, the quality of the peaks in all biological replicates was further checked. In addition, a specific number of peaks was obtained for each condition. A total of 1848 BW-specific accessible chromatin regions and 31,210 AW-specific accessible chromatin regions were identified. 

**Figure 1:**
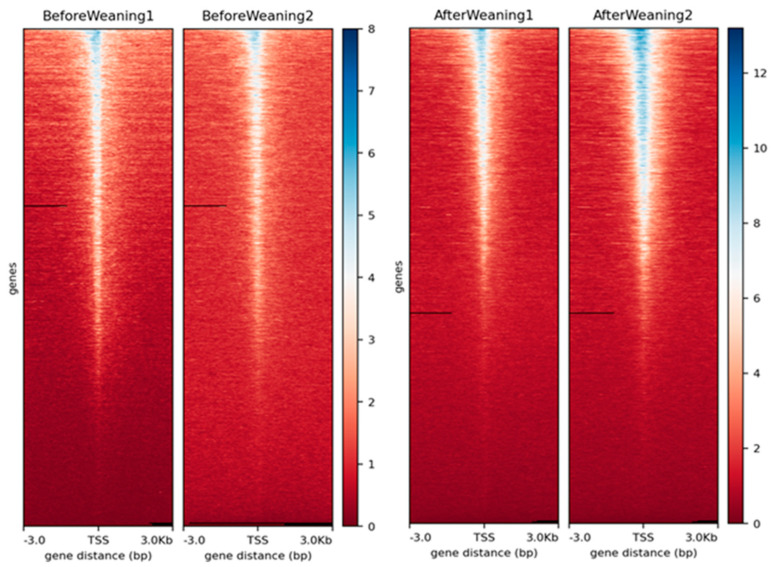
Heatmap profile of peaks relative to the transcription start sites (TSS) considering ±3 kb regions for each replicate in the weaning conditions (considering chromosomes 1–29). The blue color intensity reflects the level of peak enrichment. Each condition has two biological replicates.

Quality checks were performed to verify the quality of the peaks. The heatmap profile of peaks relative to transcription start sites (TSSs) considering ±3 kb regions for each replicate can be seen in Figure 1 and shows that the data have a good quality due to the enrichment close to the TSSs, especially in the after-weaning samples. 

**Figure 2:**
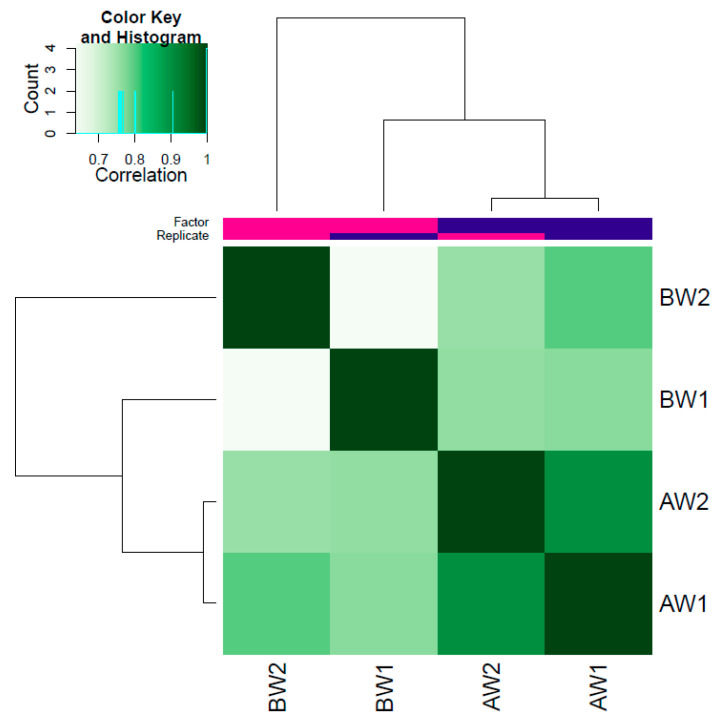
Correlation heatmap plot of replicates in the weaning conditions. BW: before weaning; AW: after weaning. Each condition has two biological replicates.

The correlation heatmap was obtained using DiffBind [22]. AW samples clustered together, showing a high correlation in the heatmap, but BW samples did not cluster together and presented a low correlation (Figure 2). The fraction of reads in peaks (FRiP) was obtained to measure the peak quality. The average FRiP for all samples was 0.11. BW2 presented the lowest FRiP of only 0.03, showing a low number of reads in peaks (Table 2). The Jaccard similarity index was obtained to measure the similarity of peaks between two samples, where 0.0 represents no overlap and 1.0 represents complete overlap. Sample BW2 had lower Jaccard scores than the other samples and showed low similarity with its biological replicate with a Jaccard index of 0.27 (Table S1). The Jaccard scores for AW samples were 0.46. Sample BW2 was excluded for subsequent analysis because it presented a low number of peaks (<30,000), a low number of reads in peaks, an FRiP of only 0.03, and a low correlation with its replicate BW1 (Table 2, Table S1, and Figure 2). 

### 3.2. Differentially Accessible Regions

The DARs were obtained using the DiffReps tool [23]. An initial total of 29,174 DARs (*p*-value < 0.05) was obtained for the AW × BW comparison (Table 3). Then, the DARs were filtered based on FDR < 0.01 and log2 fold change ≤ −1 or log2 fold change ≥ 1, and approximately 13% of the DARs were retained (Table 3). The 3818 significant DARs were then mapped against a list of 126,071 merged peaks from MACS2 (BW2 was omitted) (Table S2), which covered 1.15% of the cattle genome (Figure S3). Only the DARs that overlapped with MACS2 peaks in at least one sample were considered. Most of the DARs were mapped in the merged peaks, totaling 2907 DARs (Table S2, Table 3), and from these, 2766 unique DARs were used for further analyses. From the 2766 DARs, ~75% were classified as repressed DARs after weaning, and ~25% were induced DARs after weaning (Table 3). 

### 3.3. Annotation of Differentially Accessible Regions

Approximately 12% of DARs were in promoters with 340 DARs (Table 4, Table S3). Most of the DARs were located on distal intergenic regions (66.5%), introns (23.4%), and promoters (12.29%) (Table 4). DARs were also compared to a previous study that characterized chromatin states during weaning in cattle [15]. Most of the segments (~95%) were converted to the ARS-USD1.2 cattle reference genome assembly [17], and a total of 454,360 (BW) and 451,808 (AW) segments on 15 different chromatin states were then compared to the 2766 weaning DARs. In the BW samples, the majority of the DARs were located on ATAC states (28.25%), followed by those on enhancer-related states (EnhA, EnhAATAC, EnhWk, EnhPois, EnhPoisATAC, and EnhWkCTCFATAC) (22.03%), flanking bivalent TSS/enhancer (14.88%), and active TSSs (TssA, TssAATACCTCF, and TssAFlnk) (13.42%) (Table S4).

On AW, the majority of the DARs were located on enhancer-related states (EnhA, EnhAATAC, EnhWk, EnhPois, EnhPoisATAC, and EnhWkCTCFATAC) (28.87%), followed by active TSSs (TssA, TssAATACCTCF, and TssAFlnk) (20.55%), ATAC (15.27%), and flanking bivalent TSS/enhancer (14.40%) (Table S4).

**Figure 3:**
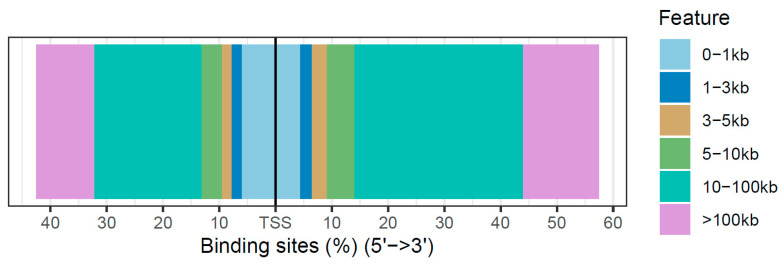
Distribution of transcription-factor-binding loci relative to the TSS of differentially accessible regions (DARs) for weaning.

In addition, the distribution of transcription-factor-binding sites relative to the TSS of the DARs for weaning was obtained (Figure 3). The majority of the DARs in the weaning fell in the 10–100 kb regions around the TSS.

### 3.4. Functional Annotation of Differentially Accessible Regions

Gene ontology (GO) enrichment analysis was performed with GREAT [27] using the 2766 unique DARs. A total of 2375 DARs were converted to hg38 (~86%). GO analysis identified significantly enriched terms (*p*-value adjusted <0.05)—71 for biological processes (BP), 22 for molecular function (MF), and 26 for cellular component (CC) (Table S5). Five significantly enriched GO terms were related to cell adhesion—regulation of cell adhesion (GO:0030155), regulation of cell-substrate adhesion (GO:0010810), regulation of cell-matrix adhesion (GO:0001952), cell adhesion molecule binding (GO:0050839), and focal adhesion (GO:0005925). In addition, six significantly enriched GO terms were related to adherens junctions - cell-substrate adherents junction (GO:0005924), cell-cell adherens junction (GO:0005913), adherens junction (GO:0005912), anchoring junction (GO:0070161), the cell-cell junction (GO:0005911), and cell-substrates junction (GO:0030055) (Table S5). Other important-significantly enriched GO terms were related to cadherin binding (GO:0045296), regulation of cell migration (GO:0030334), regulation of cell motility (GO:2000145 and GO:0048870), cell morphogenesis (GO:0000902 and GO:0022604), and the regulation of growth (GO:0040008).

In addition, an informative summary of the GO enriched terms using semantic similarity to facilitate the interpretation of GOs for weaning was plotted (Figure S4). Interesting terms were grouped in the regulation of cell migration, cell adhesion, anatomical structure morphogenesis, regulation of cellular response to stress, regulation of cytoskeleton organization, and cell morphogenesis and tube development (Figure S4A); cadherin binding and protein kinase binding (Figure S4B); anchoring junction and membrane raft and actomyosin (Figure S4C).

### 3.5. Pathway Analysis of Differentially Accessible Regions

Ingenuity pathway analysis (IPA) was used to obtain critical pathways from 1959 genes from weaning DARs. A total of 25 significant networks (network score > 20) were identified related to several essential biological functions, including cell-to-cell signaling and interaction, cellular function and maintenance, molecular transport; cell morphology, cellular assembly and organization, cellular function and maintenance; cell-to-cell signaling and interaction, DNA replication, recombination, and repair, post-translational modification; cell death and survival, organismal injury and abnormalities, renal necrosis/cell death; and cell-to-cell signaling and interaction, embryonic development, RNA post-transcriptional modification (Table S6). For canonical pathway analysis, 203 significant pathways (*p*-value < 0.01) were identified, such as TGFβ signaling, integrin-linked kinase (ILK) signaling, integrin signaling, and regulation of the epithelial–mesenchymal transition pathway (Table S7). For upstream regulators, 1435 significant regulators (*p*-value of the overlap < 0.01) were identified, such as TGFβ1 and important transcription regulators such as ATF3, BRCA1, EGR1, ETS1, ETS2, FOS, JUN, KLF4/5/6/11, SMARCA4, SMAD1/2/3/4/7, SP1, and others (Table S8). 

### 3.6. Motif Enrichment Analysis of Differentially Accessible Regions

**Figure 4:**
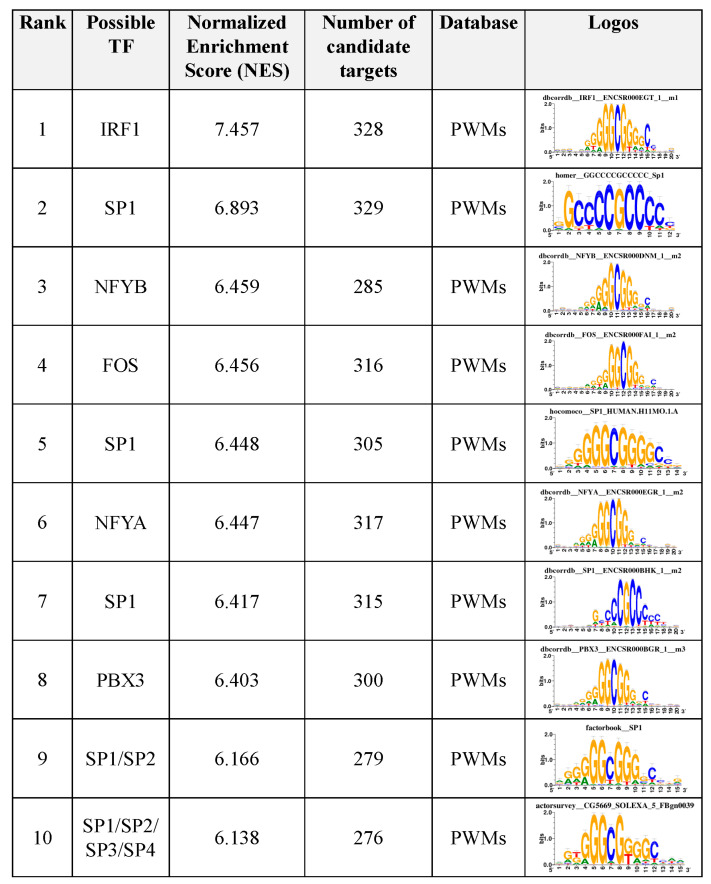
Top 10 motif enrichment discovery results on weaning differentially accessible regions, including TFs, target genes, and logos.

Enriched motifs and candidate targets were identified by the i-*cis*Target tool [29]. Results included the normalized enrichment score (NES), the area under the curve (AUC) score normalized by subtracting the mean of all AUC overall motifs and dividing it by the standard deviation for possible TFs, and candidate targets. The top 10 enriched motifs detected were IRF1, SP1 (HOMER), NFYB, FOS, SP1 (HOCOMOCO), NFYA, SP1 (DbcorrDB), PBX3, SP1/2 (Factorbook), and SP1/2/3/4 (FlyFactorSurvey) (Figure 4, File S1). In addition, 12 TFs previously determined in a study of weaning in cattle rumen tissue [10] were identified by the i-*cis*Target tool [29]—ATF3, ATF4, BRCA1, EGR1, ETS1, EZH2, FOS, KLF10, POLR2A, SMARCA4, SREBF2, and YY1.

### 3.7. Co-Expression and Network Visualization of Critical Pathways for Rumen

Gene co-expression analysis was conducted to validate essential pathways and construct gene networks. A total of 19,810 genes was generated by RNA-seq analysis and utilized for the WGCNA analysis [32], and 37 merged modules were generated, ranging from 22 to 3738 genes per module (Figure S5). Because of their potential roles in cellular adhesions, the TGFβ signaling pathway [34,35] and integrin-linked kinase (ILK) signaling pathway [36,37] were selected to study their biological relevance in rumen development during weaning in cattle (Table S7). Co-expression information and genes from each significant canonical pathway selected were utilized to construct the networks.

**Figure 5:**
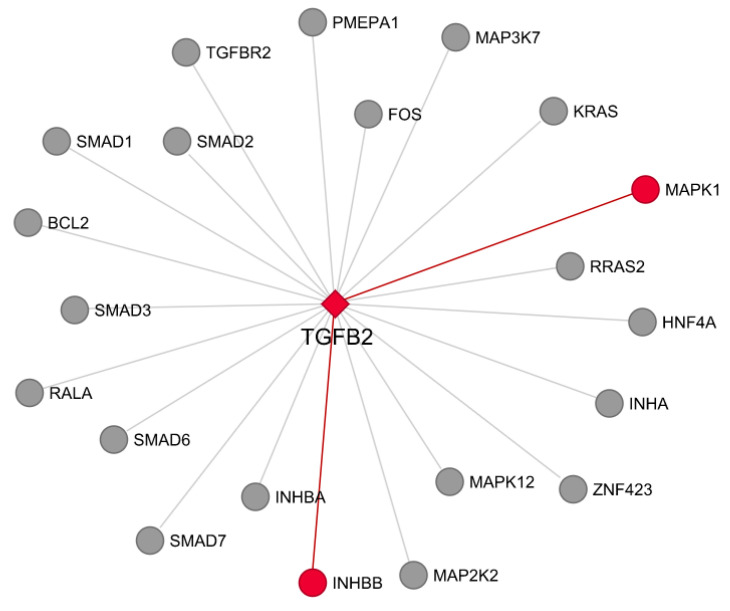
TGFβ signaling pathway network. Genes included are from the IPA canonical pathway, and all of them are in differentially accessible regions (DARs) in weaning. Red edges represent significant co-expressed genes (>0.8) using RNA-seq data. The *TGFβ2* was selected as the hub gene for the network.

Twenty-two genes annotated in DARs in weaning are part of the TGFβ pathway, such as *TGFB2* (located on repressed DARs for AW, Table S3), *TGFBR2* (located on repressed DARs), *FOS* (located on repressed DARs), *SMADs* (all located on repressed DARs), *MAPKs* (all located on repressed DARs), and others (Figure 5, Table S7). The *TGFB2* was selected as the hub gene for the network. The *TGFB1* gene was not included because no differentially accessible chromatin region was identified near this gene. From the 22 genes present in the network, two genes, *MAPK1* and *INHBB* (both located on repressed DARs for AW, Table S3), showed a high co-expression (>0.8) with *TGFB2* (Figure 5). 

**Figure 6:**
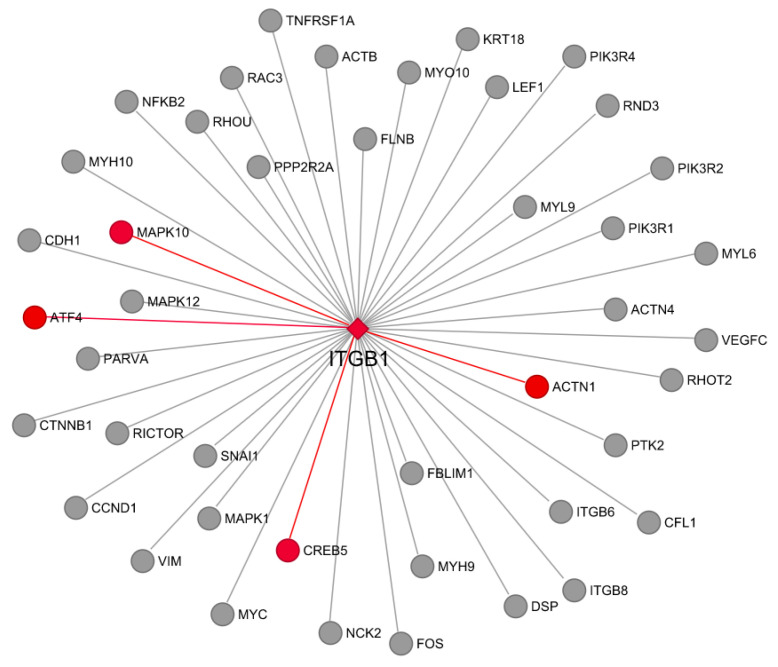
Integrin-linked kinase (ILK) signaling pathway network. Genes included are from the IPA canonical pathway, and all of them are in differentially accessible regions (DARs) in weaning. Red edges represent significant co-expressed genes (>0.8) using RNA-seq data. The *ITGB1* was selected as the hub gene for the network.

Another vital pathway selected was the integrin-linked kinase (ILK) signaling. The *ITGB1* gene was chosen as the hub gene for the network (located on a repressed DAR for AW, Table S3). From the 44 genes present in the network, four genes (all located on repressed DARs for AW, Table S3), *ACTN1*, *ATF4*, *MAPK10,* and *CREB5*, showed a high co-expression (>0.8) with *ITGB1* (Figure 6).

## 4. Discussion

The Encyclopedia of DNA Elements (ENCODE) Consortium is an international research group that aims to identify functional elements in humans, develop standardized protocols, and determine best practices [38]. The ATAC-seq technology was developed to characterize active regulatory elements and discover essential functions of the noncoding genome. The ENCODE guidelines for ATAC-seq projects recommend 50 million reads for paired-end sequencing for each replicate (https://www.encodeproject.org/atac-seq/, accessed on 6 September 2021). Recent studies in cattle identified accessible chromatin regions in different tissues, including muscle, bronchial lymph nodes, liver, lung, hypothalamus, brain, adipose, and spleen [12,13,14,39]; however, none of them evaluated rumen tissue. This study aimed to identify accessible chromatin regions and genomic regulatory elements that may control rumen epithelial development changes during weaning in calves.

The quality standard was met for AW with more than 50 M clean reads; however, BW samples exhibited less than the recommended number of reads. The initial number of reads obtained for BW was above the recommended 50 M, but this number was reduced by almost 40% after cleaning. The quality of the identified peaks for replicates was checked at different steps, using the fraction of reads in peaks (FRiP score), the Jaccard similarity index, and a correlation heatmap. Although most human ENCODE data sets have an FRiP > 0.1 and the 1% FRiP is acceptable for large mammalian genomes with thousands of occupancy sites [40], the ENCODE guidelines recommended a minimum FRiP score of 0.2 for ATAC-seq. The FRiP score was met only by BW1 but not BW2, for which the score was only 0.03. The Jaccard similarity index, a measurement of the sample similarity, indicated that BW2 had low similarity with its biological replicate with a Jaccard index of only 0.27. A previous study in cattle using the ATAC-seq approach also utilized the Jaccard index, and samples with low scores were removed [12]. In addition, the correlation heatmap revealed that AW samples clustered together, showing a high correlation, but BW samples presented a low correlation between them. All these quality measurements together indicate that BW2 was not appropriate to be used for further analysis. The low quality of the BW2 sample may have been a consequence of poor sample preparation, which should be monitored more carefully in future studies.

Additional steps were performed to minimize the impact of using one replicate for BW and ensure the quality of the identified DARs. The DiffReps software was utilized to detect DARs. This tool scans the genome for enrichment regions using a sliding window method to see differential chromatin sites. It provides the choice of several statistical tests, including the G-test that can be applied when there are no replicates [23]. Results indicated that DiffReps is a highly sensitive software for detecting differential sites from ChIP-seq data [23]. Additional steps were performed to ensure the high-quality results of the identified DARs from DiffReps, including filtration of DARs with an FDR < 0.01 and log2 fold change ≤ −1 or log2 fold change ≥ 1, and only DARs that had overlap with MACS2 peaks were considered for further analysis. DiffReps does not utilize peaks generated by MACS2 as input, and the choice of viewing only DARs that overlapped with MACS peaks was also adopted earlier in a study in mice [24].

A total of 340 DARs were in promoter regions. However, most of the DARs were in distal intergenic regions (1622) and introns (565), and most of the DARs were 10–100 kb away from the TSS, indicating that most of the ATAC-seq sites could be distal enhancers in cattle rumen tissue. A previous study in cattle also identified that most of the peaks in the hypothalamus and muscle were in distal intergenic regions and 10–100 kb away from the TSS [13]. Enhancers can regulate gene expression from over 1 million base pairs from the promoters in mammals and can be found within introns of neighboring genes [41,42]. A recent study of the chromatin states in rumen cattle during the weaning [15] revealed that weak enhancers and flanking active transcriptional start sites were the most dynamic states during weaning. Interestingly, overlapping DARs with 15 chromatin states previously identified in cattle rumen tissue [15] revealed that most DARs were located on enhancer and ATAC states.

Gene ontology enrichment results in combination with semantic similarity analysis revealed important enriched GO terms related to cell adhesion, anchoring junction, cadherin binding, regulation of cell migration, regulation of cell motility, cell morphogenesis, regulation of growth, regulation of cytoskeleton organization, tube development, anatomical structure morphogenesis, and membrane raft and actomyosin. Cell adhesion is the process through which cells attach to each other and the extracellular matrix, and it is essential for the development of several tissues. The study of cell adhesion has been of great interest to researchers due to its fundamental role in cell regulation and proliferation for the development and maintenance of tissues [43]. As mentioned before, the weaning process results in dramatic structural changes in the rumen epithelium, including an increase of height and width of the rumen papillae [4]; in this context, this study showed that weaning might have an essential effect on the regulation of cellular adhesions.

IPA analyses have also identified networks of biological relevance (cell-to-cell signaling and interaction, cellular function and maintenance, molecular transport, cell morphology, cellular assembly and organization, and others); canonical pathways (TGFβ, integrin-linked kinase, integrin signaling, and regulation of the epithelial–mesenchymal transition, and others), and upstream regulators (TGFβ1, FOS, JUN, ATF3, BRCA1, EGR1, ETS1/2, KLF4/5/6/11, SMAD1/2/3/4/7, SMARCA4, SP1, and others). In addition, TGFβ and ILK signaling pathways were selected due to their relevance in rumen biology and their potential role in the cellular adhesions [34,35,36,37]. TGFβ1 (transforming growth factor-β 1) is a secreted protein member of the transforming growth factor-β superfamily of cytokines that has several functions, including cell growth, proliferation and differentiation, and apoptosis [44]. Moreover, TGFβ1 can affect cell adhesion, according to studies conducted in humans, sheep, and cattle [45,46,47]. As previously reported in the cattle [48], during weaning, TGFβ1 was identified as a putative mediator of rumen epithelial tissue development. A recent study in cattle also suggested that TGFβ is a potential epithelial cell candidate gene [10]. Although TGFβ1 was not identified in an accessible region in this study, other related proteins were found, such as TGFβ2 and TGFβR2, and may have similar roles in cattle rumen. *TGFβ2* and *TGFβR2* genes were located on repressed DARs for AW and indicated that these two genes are possibly more active before and during weaning to stimulate the growth and development of the rumen. The *ITGB1, ITGB6* and *ITGB8* genes are part of the ILK network. Integrin proteins are cell adhesion molecules and have essential functions in cell migration, such as ITGB1 [49]. The *ITGB1* gene was identified as a potential cattle epidermal rumen marker in response to diet [50]. In this study, the *ITGB1* gene was also located on a repressed DAR for AW, showing that this gene is induced before weaning. Another study suggested that *ITGB1* is associated with the *PEAR1* gene and affects bovine cell migration and differentiation [51].

Furthermore, motif enrichment analyses revealed important candidate TFs for weaning, such as ATFs (1–7), ETS1, FOS, IRF1, KLFs (2, 3, 6–18), NFYA/B, PBX3, SMARCA4, and SPs (1–9). The same TFs were previously identified in a study evaluating weaning in Holstein ruminal epithelial tissue [10], including ATF3, BRCA1, EGR1, ETS1, EZH2, FOS, KLF10, POLR2A, SMARCA4, SREBF2, and YY1. These TFs have essential roles in cellular processes and development. The activator Protein-1 (AP1) transcription factor family has several members, including JUN and ATF3, and they are involved in cell proliferation and differentiation and death [52]. SP1 is involved in several processes, such as cell differentiation and proliferation, apoptosis, chromatin remodeling, and immune responses [53]. Similar to ATF3, ETS is involved in the cell differentiation and proliferation [54]. NFY regulates the transcription of many genes. A study in cattle identified that NFY regulates the PIA promoter activity, which is dominantly active in lipogenic tissues under favorable nutritional conditions [55]. SMARCA4 regulates cell differentiation and embryonic development in the cattle [56]. ETS1 regulates *FGF1* and induces angiogenesis [57]. Krüppel-like factors (KLFs) are part of the zinc-finger family of TFs and are closely related to the SP family and regulate several critical development processes by activating/repressing many genes [58,59]. KLF4 regulates cell proliferation, differentiation, and adipogenesis [58,60,61]. KLF10 is induced by TGFβ and is implicated in cell differentiation, apoptosis, osteoblast and osteoclast differentiation, gluconeogenesis, and inflammation [62,63,64,65]. An evolutionary study on genomic rearrangements in ruminants identified 25 TFs, including KLF4/5 and SP1 [66]. These TFs were enriched in the liver, suggesting their essential roles in ruminants regulating cell proliferation, differentiation, and metabolism, which are all responsive to changes in nutritional status.

## 5. Conclusions

This study evaluated the effect of weaning in cattle using the ATAC-seq approach to identify and characterize genome-wide differential open chromatin regions and regulatory elements. Open chromatin regions were identified for pre- and post-weaning, generating over 2700 DARs, showing their potential roles in rumen development in cattle. Functional analyses, including gene ontology enrichment, pathways, motif enrichment, and co-expression, were conducted on the DARs to explore putative biological functions, revealing crucial enhancers, transcription factors, and candidate target genes for rumen development during weaning. Downstream analyses revealed enriched GO terms related to cell adhesion, anchoring junction, cell migration, motility, and pathways of biological relevance (TGFβ and ILK), which were also associated with cell adhesion. Differentially accessible regions in weaning were identified for the first time in this study, revealing enhancers, TFs, and candidate target genes that represent potential biomarkers for the rumen biology and weaning process in cattle. These biomarkers will be useful as a molecular tool in future rumen nutrition and developmental studies.

## Figures and Tables

**Table 1:** Sequence read statistics showing the number of reads, number and percentages of mapped reads, mitochondrial reads, duplicate reads and reads with mapping quality < 10, and the number of clean reads used for peak calling.

Condition	No. of Reads	No. of Mapped Reads	% of Mapped Reads	No. of MT Reads	% of MT Reads ^1^	No. of Duplicate Reads	% of Duplicate Reads ^1^	No. of MQ < 10 Reads	% of MQ < 10 Reads ^1^	No. of Clean Reads ^2^
BW1	61,550,092	59,937,035	97.38	4,456,644	7.44	1,537,436	2.57	17,496,479	29.19	36,963,828
BW2	77,759,870	69,274,392	89.09	4,286,235	6.19	823,517	1.19	22,547,946	32.55	48,310,541
AW1	90,778,622	88,547,917	97.54	5,222,533	5.90	1,261,956	1.43	26,047,668	29.42	56,315,258
AW2	88,648,740	86,493,739	97.57	6,070,031	7.02	1,269,929	1.47	23,577,286	27.26	55,901,495
Total	318,737,324	304,253,083	−	20,035,443	−	4,892,838	−	89,669,379	−	197,491,122
Average	79,684,331	76,063,271	95.40	5,008,861	6.63	1,223,210	1.66	22,417,345	29.60	49,372,781

BW: before weaning. AW: after weaning. MQ: mapping quality. Each condition has two biological replicates. ^1^ Percentages were calculated considering the total number of mapped reads. ^2^ Reads uniquely mapped, MQ > 10, no duplicate reads or reads located on MT chromosome.

**Table 2:** Peak calling metrics showing the total number of clean reads used to call peaks and calculate the fraction of reads in peaks (FRiP), MACS2 peaks (FDR < 0.05), assigned reads in peaks, FRiP, average of peak lengths, and proportion of peaks near the TSS (±3 Kb, %).

Condition	No. of Clean Reads ^1^	No. of Clean Reads Used for FRiP ^2^	No. of MACS2 Peaks ^2^	No. of Assigned Reads in Peaks ^2^	FRiP ^3^	Average Peak Length	Proportion of Peaks Near TSS (±3 Kb, %)
BW1	36,963,828	36,056,626	94,963	7,267,896	0.20	171	16.08
BW2	48,310,541	46,833,278	27,640	1,483,647	0.03	210	25.33
AW1	56,315,258	54,744,212	65,523	4,449,195	0.08	211	18.70
AW2	55,901,495	54,363,957	86,807	7,436,305	0.13	253	16.55
Total	197,491,122	191,998,073	274,933	20,637,043	−	−	−
Average	49,372,781	47,999,518	68,733	5,159,261	0.11	211	19.17

BW: before weaning. AW: after weaning. Each condition has two biological replicates. ^1^ Reads uniquely mapped, with MQ > 10, no duplicate reads, or reads located on MT chromosome. ^2^ Reads located on chromosome X and unplaced reads were not included. ^3^ Fraction of reads in peaks.

**Table 3:** Number of differentially accessible regions (DARs) for after weaning vs. before weaning comparison, significant DARs (FDR < 0.01 and −1 ≤ log2FC ≥ 1), significant DARs that overlapped with peaks, and unique significant DARs that coincided with peaks, including induced and repressed DARs.

After Weaning × Before Weaning DARs	No. of DARs	DARs%
DARs initially identified (*p*-value < 0.05)	29,174	−
Significant DARs (FDR < 0.01 and −1 ≤ log2FC ≥ 1)	3818	13.09
Significant DARs that overlapped with peaks	2907	9.96
Unique significant DARs that overlapped with peaks	2766	−
Induced DARs with log2FC ≥ 1	686	24.80
Repressed DARs with log2FC ≤ −1	2080	75.20

**Table 4:** Annotation of differentially accessible regions (DARs) for weaning.

Feature	Number	Frequency (%)
Promoter (<1 kb)	289	10.45
Promoter (1–2 kb)	51	1.84
5’ UTR	1	0.04
3’ UTR	43	1.55
Exon	135	4.88
First Intron	153	5.53
Other Intron	412	14.90
Downstream (<1 kb)	15	0.54
Downstream (1–2 kb)	23	0.83
Downstream (2–3 kb)	22	0.80
Distal Intergenic	1622	58.64
Total	2766	100.00

## Data Availability

All high-throughput sequencing data analyzed in this study are deposited in NCBI. RNA-seq data are publicly available at the NCBI SRA database (BioProject ID: PRJNA658627). All ATAC-seq data were submitted to NCBI, SRA database (BioProject ID: PRJNA672996).

## Supplementary Materials

The following are available online at https://www.mdpi.com/article/10.3390/genes13030535/s1, Figure S1: Fragment size distribution of ATAC-seq reads in weaning. BW: before weaning; AW: after weaning. Each condition has two biological replicates. Figure S2: Chromosomal distribution of peaks detected by MACS2 (FDR < 0.05) in weaning. BW: before weaning; AW: after weaning. Each condition has two biological replicates. Figure S3: Distribution of merged peaks by chromosome for the weaning condition (considering chromosomes 1–29). Figure S4: Scatterplot of enriched GO terms (*p*-value adjusted < 0.05) for genes associated with weaning differentially accessible regions (DARs) using semantic similarity for biological process (A), molecular function (B), and cellular component (C). Figure S5: Dendrogram showing the gene co-expression network constructed using WGCNA from 19,810 genes for weaning. The branches of the hierarchical cluster tree and color bands represent the assigned module. The color bar labeled “Dynamic Tree Cut” beneath the dendrogram represents the initial module assignment of each gene, and the color bar labeled “Merged dynamic” represents the 37 merged modules grouped by similar gene expression profiles. Table S1: Matrix of the number of peaks per sample, the number of peak intersections between pairwise comparisons, and the Jaccard similarity score between pairwise comparisons for weaning samples. The top of the diagonal (light red) is the number of peak intersections between pairwise comparisons. The yellow diagonal line is the number of peaks per sample. The bottom of the diagonal (green) is the Jaccard score between pairwise comparisons. Each condition has two biological replicates. Table S2: Merged peaks information from the weaning condition (after × before weaning), and differentially accessible regions (DARs) that overlapped with the merged peaks (FDR < 0.01 and log2 fold change ≤ −1 or log2 fold change ≥ 1). Table S3: Weaning differentially accessible regions (DARs) with annotation information. Table S4: Fold of enrichments for differentially accessible regions (DARs) across 15 chromatin states in epithelial cells in cattle. Table S5: Enriched gene ontology (GO) terms (*p*-value adjusted < 0.05) for genes associated with weaning differentially accessible regions (DARs). Biological process (BP), molecular function (MF), and cellular component (CC). Table S6: Significant networks (network score > 20) were obtained of genes associated with DARs from the weaning condition. Table S7: Significant canonical pathways (*p*-value < 0.01) of genes associated with DARs from the weaning condition. Table S8: Significant upstream regulators (*p*-value of overlap < 0.01) of genes associated with DARs from the weaning condition. File S1: List all enriched regulatory elements in rumen tissue for weaning differentially accessible regions generated by the i-*cis*Target tool.
