# Supplementary material for: Characterization of Accessible Chromatin Regions in Cattle Rumen Epithelial Tissue during Weaning
Source: Genes (Basel). 2022 Mar 18;13(3):535. doi: 10.3390/genes13030535 (PMC8949786; doi:10.3390/genes13030535)
Supplement: Supplementary file 1 [file genes-13-00535-s001.zip › Figure S1.pptx]

## Slide 1
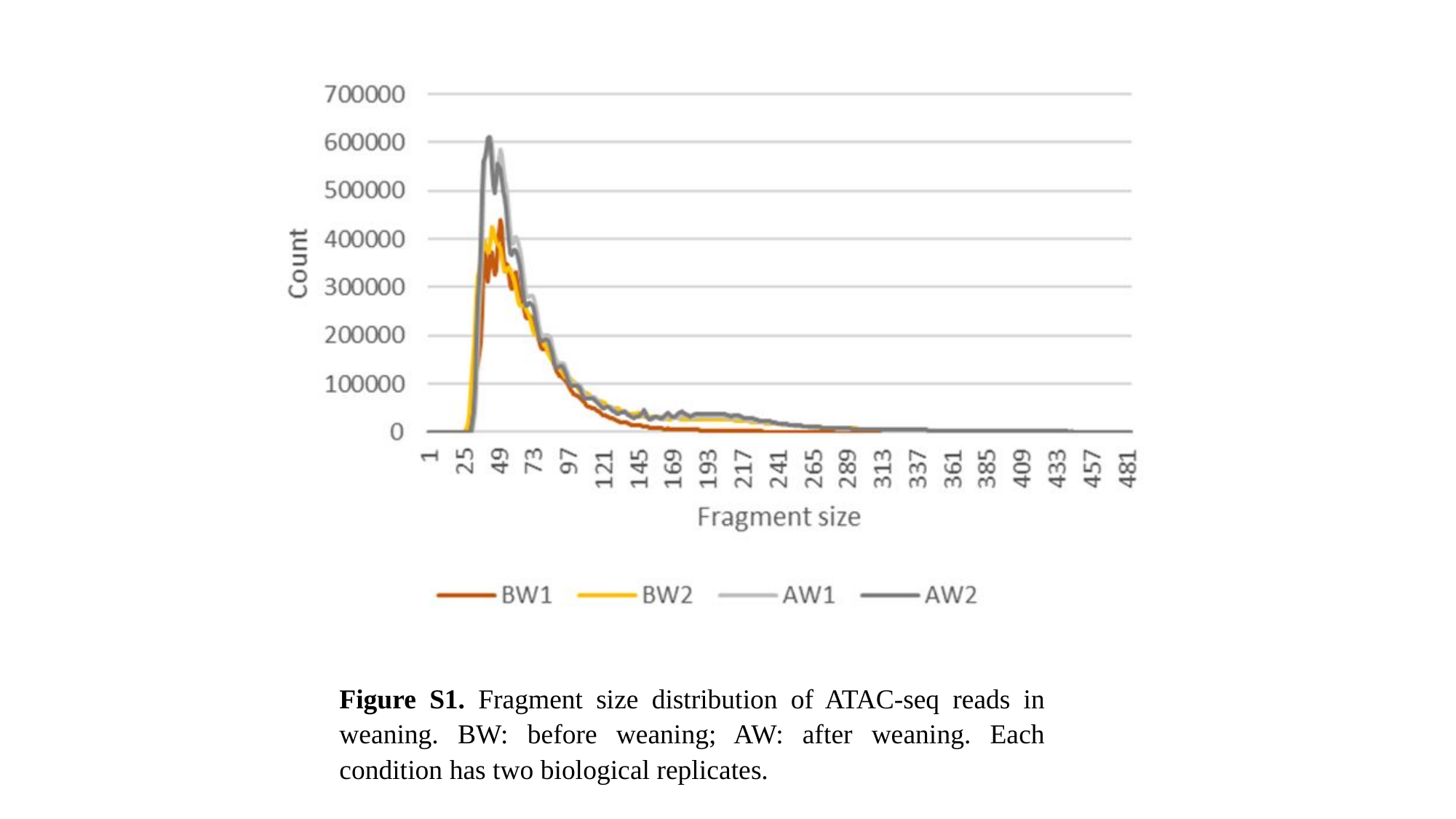

Figure S1. Fragment size distribution of ATAC-seq reads in weaning. BW: before weaning; AW: after weaning. Each condition has two biological replicates.
