# Supplementary material for: Characterization of Accessible Chromatin Regions in Cattle Rumen Epithelial Tissue during Weaning
Source: Genes (Basel). 2022 Mar 18;13(3):535. doi: 10.3390/genes13030535 (PMC8949786; doi:10.3390/genes13030535)
Supplement: Supplementary file 1 [file genes-13-00535-s001.zip › Figure S4.pptx]

## Slide 1
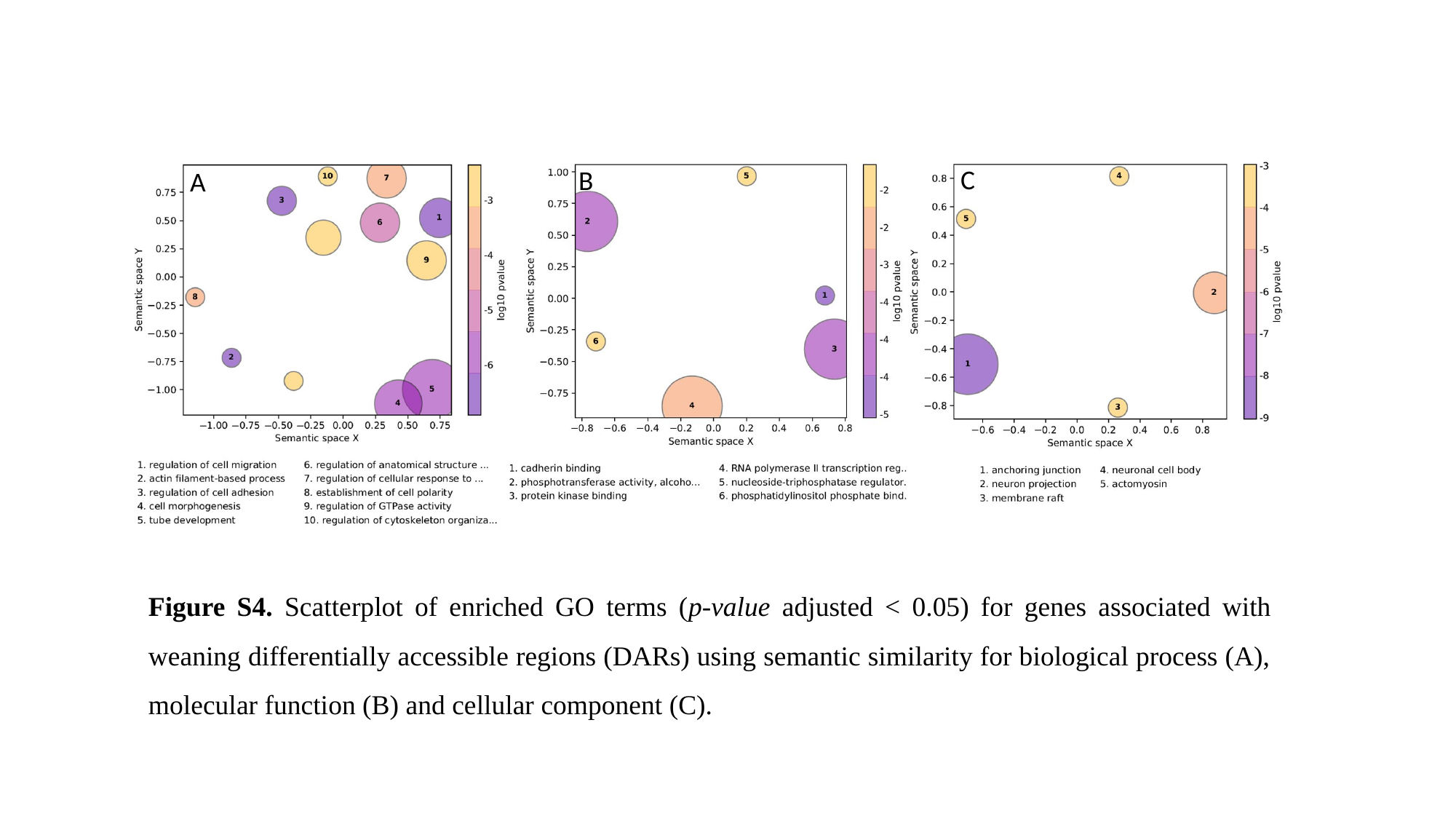

C
B
A
Figure S4. Scatterplot of enriched GO terms (p-value adjusted < 0.05) for genes associated with weaning differentially accessible regions (DARs) using semantic similarity for biological process (A), molecular function (B) and cellular component (C).
