# Supplementary material for: Characterization of Accessible Chromatin Regions in Cattle Rumen Epithelial Tissue during Weaning
Source: Genes (Basel). 2022 Mar 18;13(3):535. doi: 10.3390/genes13030535 (PMC8949786; doi:10.3390/genes13030535)
Supplement: Supplementary file 1 [file genes-13-00535-s001.zip › File S1.pdf]

i-cisTarget

An integrative genomics method for the prediction of regulatory features and cis-regulatory modules.

Parameters and statistics for clab4

Number of features	29890
Number of enriched features (NES > 3.0)	512
Total number of ranked regions	1223024
Type of input query	bed
Number of **i-cisTarget regions in input set**	2192 (**Results of the region mapping**)
Minimum fraction of overlap	0.4
Normalized enrichment score (NES) threshold	3.0
AUC threshold (fraction / # of ranked regions)	0.005 (6115)
Recovery curve threshold (# of regions)	20000

AUC distribution

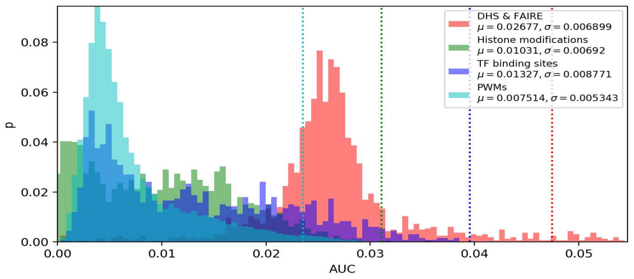

Recovery of best feature

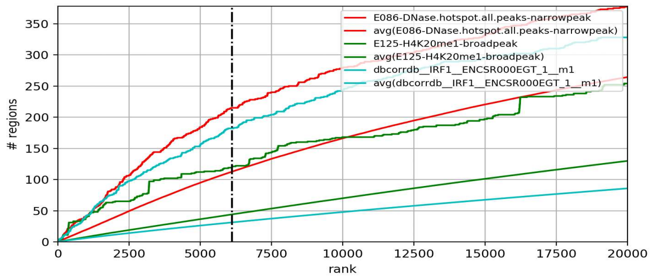

Results for clab4

Select features in the table below, select an operation and proceed.

1. Use candidate target regions as **filter** and use as input for i-cisTarget again.
2. **Scan** candidate target regions of selected features for multiple homotypic CRMs.
3. **Create SIF file** for the selected features.

This report is also available as an **archive**.

For some databases, randomly generated features were enriched. Also, non-random features in these same databases that have a lower enrichment than the first randomly generated enriched feature were removed from the report. The involved databases and their adjusted NES thresholds are: TF binding sites (3.00000).

#	Feature	NES	Logo	Recovery Curve	Candidate targets	All regions in top 20000	Database
1	dbcorrdb__IRF1__ENCSR000EGT_1__m1<br>Description: IRF1 (ENCSR000EGT-1, motif 1)<br>Possible TFs: IRF1	*7.45770*	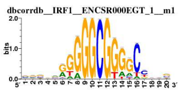	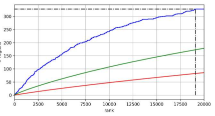	**link**	**link**	PWMs
2	homer__GGCCCCGCCCCC_Sp1<br>Description: Sp1(Zf)/Promoter/Homer	*6.89365*	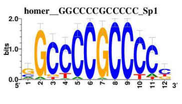	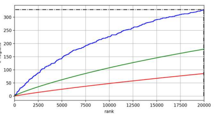	**link**	**link**	PWMs
3	dbcorrdb__NFYB__ENCSR000DNM_1__m2<br>Description: NFYB (ENCSR000DNM-1, motif 2)<br>Possible TFs: NFYB	*6.45966*	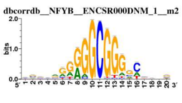	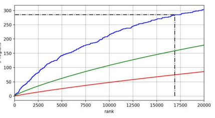	**link**	**link**	PWMs

#	Feature	NES	Logo	Recovery Curve	Candidate targets	All regions in top 20000	Database
4	☐ dbcorrdb__FOS__ENCSR000FAI_1__m2<br>Description: FOS (ENCSR000FAI-1, motif 2)<br>Possible TFs: FOS	*6.45612*	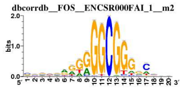	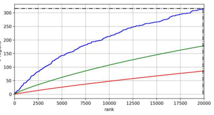	**link**	**link**	PWMs
5	☐ hocomoco__SP1_HUMAN.H11MO.1.A<br>Description: SP1_HUMAN<br>Possible TFs: SP1	*6.44868*	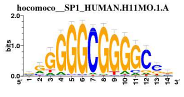	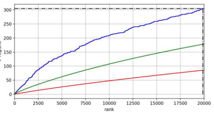	**link**	**link**	PWMs
6	☐ dbcorrdb__NFYA__ENCSR000EGR_1__m2<br>Description: NFYA (ENCSR000EGR-1, motif 2)<br>Possible TFs: NFYA	*6.44724*	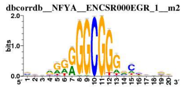	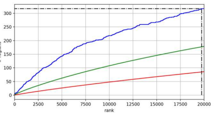	**link**	**link**	PWMs
7	☐ dbcorrdb__SP1__ENCSR000BHK_1__m2<br>Description: SP1 (ENCSR000BHK-1, motif 2)<br>Possible TFs: SP1	*6.41714*	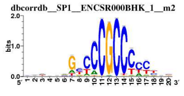	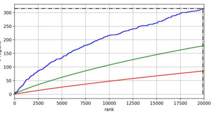	**link**	**link**	PWMs
8	☐ dbcorrdb__PBX3__ENCSR000BGR_1__m3<br>Description: PBX3 (ENCSR000BGR-1, motif 3)<br>Possible TFs: PBX3	*6.40358*	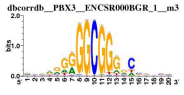	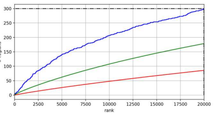	**link**	**link**	PWMs
9	☐ factorbook__SP1<br>Description: SP1<br>Possible TFs: SP1, SP2	*6.16652*	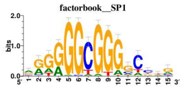	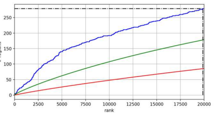	**link**	**link**	PWMs
10	☐ flyfactorsurvey__CG5669_SOLEXA_5_FBgn0039169<br>Description: CG5669_SOLEXA_5_FBgn0039169<br>Possible TFs: SP1, SP2, SP3, SP4	*6.13867*	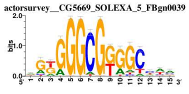	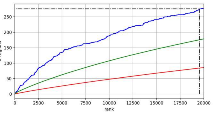	**link**	**link**	PWMs
11	☐ cisbp__M4871<br>Description: CG5669[gene ID: "FBgn0039169" species: "Drosophila melanogaster" TF status: "direct" TF family: "C2H2 ZF" DBDs: "zf-C2H2"]; SP5[gene ID: "ENSG00000204335" species: "Homo sapiens" TF status: "inferred" TF family: "C2H2 ZF" DBDs: "zf-C2H2"]; SP6[gene ID: "ENSG00000189120" species: "Homo sapiens" TF status: "inferred" TF family: "C2H2 ZF" DBDs: "zf-C2H2"]; SP7[gene ID: "ENSG00000170374" species: "Homo sapiens" TF status: "inferred" TF family: "C2H2 ZF" DBDs: "zf-C2H2"]; SP9[gene ID: "ENSG00000217236" species: "Homo sapiens" TF status: "inferred" TF family: "C2H2 ZF" DBDs: "zf-C2H2"]; Sp2[gene ID: "ENSMUSG00000018678" species: "Mus musculus" TF status: "inferred" TF family: "C2H2 ZF" DBDs: "zf-C2H2"]; Sp3[gene ID: "ENSMUSG00000027109" species: "Mus musculus" TF status: "inferred" TF family: "C2H2 ZF" DBDs: "zf-C2H2"]; Sp5[gene ID: "ENSMUSG00000075304" species: "Mus musculus" TF status: "inferred" TF family: "C2H2 ZF" DBDs: "zf-C2H2"]; Sp6[gene ID: "ENSMUSG00000038560" species: "Mus musculus" TF status: "inferred" TF family: "C2H2 ZF" DBDs: "zf-C2H2"]; Sp7[gene ID: "ENSMUSG00000060284" species: "Mus musculus" TF status: "inferred" TF family: "C2H2 ZF" DBDs: "zf-C2H2"]; Sp8[gene ID: "ENSMUSG00000048562" species: "Mus musculus" TF status: "inferred" TF family: "C2H2 ZF" DBDs: "zf-C2H2"]; Sp9[gene ID: "ENSMUSG00000068859" species: "Mus musculus" TF status: "inferred" TF family: "C2H2 ZF" DBDs: "zf-C2H2"]<br>Possible TFs: SP1, SP2, SP3, SP4	*6.01825*	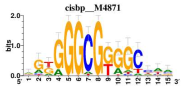	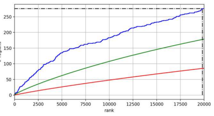	**link**	**link**	PWMs

#	Feature	NES	Logo	Recovery Curve	Candidate targets	All regions in top 20000	Database
12	☐ dbcorrdb__NFYA__ENCSR000DNS_1__m2<br>Description: NFYA (ENCSR000DNS-1, motif 2)<br>Possible TFs: NFYA	*5.95460*	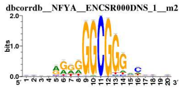	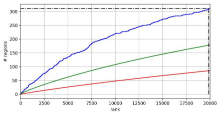	link	link	PWMs
13	☐ dbcorrdb__STAT1__ENCSR000DZM_1__m3<br>Description: STAT1 (ENCSR000DZM-1, motif 3)<br>Possible TFs: STAT1	*5.81373*	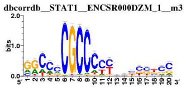	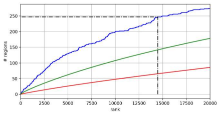	link	link	PWMs
14	☐ taipale__SP4_full_NWRGCCMCGCCCMCTNN<br>Description: SP4 monomeric C2H2 full<br>Possible TFs: SP4	*5.76850*	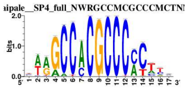	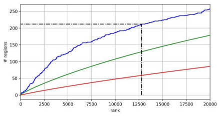	link	link	PWMs
15	☐ dbcorrdb__FOS__ENCSR000EYZ_1__m2<br>Description: FOS (ENCSR000EYZ-1, motif 2)<br>Possible TFs: FOS	*5.76815*	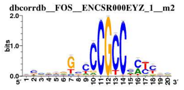	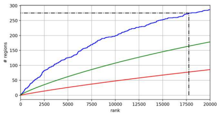	link	link	PWMs
16	☐ dbcorrdb__NFYB__ENCSR000DNR_1__m2<br>Description: NFYB (ENCSR000DNR-1, motif 2)<br>Possible TFs: NFYB	*5.66135*	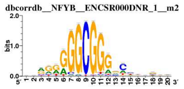	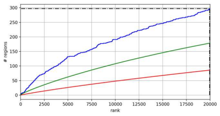	link	link	PWMs
17	☐ hocomoco__SP1_HUMAN.H11MO.0.A<br>Description: SP1_HUMAN<br>Possible TFs: SP1	*5.65861*	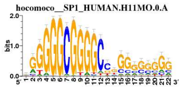	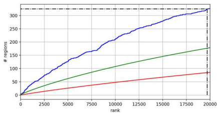	link	link	PWMs
18	☐ taipale_cyt_meth__KLF15_NCCMCGCCCMYN_FL_meth<br>Description: KLF15 [Znf_C2H2, CpG-meth]<br>Possible TFs: KLF15	*5.58215*	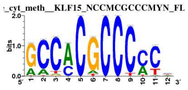	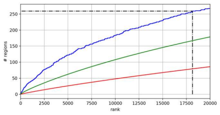	link	link	PWMs
19	☐ swissregulon__hs__SP1.p2<br>Description: hs__SP1.p2<br>Possible TFs: SP1	*5.48627*	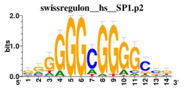	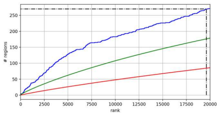	link	link	PWMs
20	☐ dbcorrdb__ATF3__ENCSR000DOG_1__m1<br>Description: ATF3 (ENCSR000DOG-1, motif 1)<br>Possible TFs: ATF3	*5.47379*	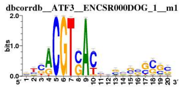	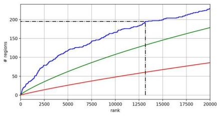	link	link	PWMs
21	☐ hocomoco__KLF12_HUMAN.H11MO.0.C<br>Description: KLF12_HUMAN<br>Possible TFs: KLF12	*5.38024*	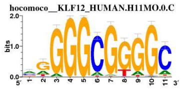	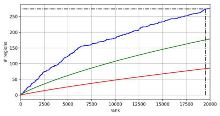	link	link	PWMs
22	☐ taipale_cyt_meth__SP1_NWRGCCACGCCCMYN_eDBD<br>Description: SP1 [Znf_C2H2]<br>Possible TFs: SP1	*5.33642*	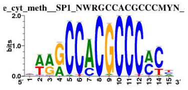	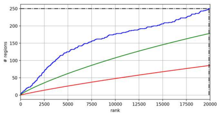	link	link	PWMs
23	☐ transfac_pro__M04744<br>Description: V$ATF3_02: ATF-3<br>Possible TFs: ATF3	*5.33428*	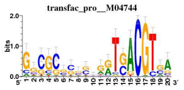	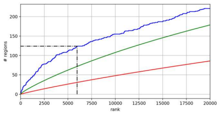	link	link	PWMs
24	☐ flyfactorsurvey__Sp1_SOLEXA_2.5_FBgn0020378<br>Description: Sp1_SOLEXA_2.5_FBgn0020378<br>Possible TFs: SP8, SP9, SP6, SP7	*5.29264*	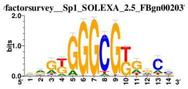	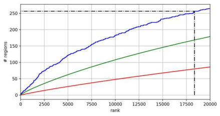	link	link	PWMs

#	Feature	NES	Logo	Recovery Curve	Candidate targets	All regions in top 20000	Database
25	☐ cisbp__M5855<br>Description: SP4[gene ID: "ENSG00000105866" species: "Homo sapiens" TF status: "direct" TF family: "C2H2 ZF" DBDs: "zf-C2H2"]; SP5[gene ID: "ENSG00000204335" species: "Homo sapiens" TF status: "inferred" TF family: "C2H2 ZF" DBDs: "zf-C2H2"]; SP6[gene ID: "ENSG00000189120" species: "Homo sapiens" TF status: "inferred" TF family: "C2H2 ZF" DBDs: "zf-C2H2"]; SP7[gene ID: "ENSG00000170374" species: "Homo sapiens" TF status: "inferred" TF family: "C2H2 ZF" DBDs: "zf-C2H2"]; SP9[gene ID: "ENSG00000217236" species: "Homo sapiens" TF status: "inferred" TF family: "C2H2 ZF" DBDs: "zf-C2H2"]; Sp2[gene ID: "ENSMUSG00000018678" species: "Mus musculus" TF status: "inferred" TF family: "C2H2 ZF" DBDs: "zf-C2H2"]; Sp3[gene ID: "ENSMUSG00000027109" species: "Mus musculus" TF status: "inferred" TF family: "C2H2 ZF" DBDs: "zf-C2H2"]; Sp5[gene ID: "ENSMUSG00000075304" species: "Mus musculus" TF status: "inferred" TF family: "C2H2 ZF" DBDs: "zf-C2H2"]; Sp6[gene ID: "ENSMUSG00000038560" species: "Mus musculus" TF status: "inferred" TF family: "C2H2 ZF" DBDs: "zf-C2H2"]; Sp7[gene ID: "ENSMUSG00000060284" species: "Mus musculus" TF status: "inferred" TF family: "C2H2 ZF" DBDs: "zf-C2H2"]; Sp8[gene ID: "ENSMUSG00000048562" species: "Mus musculus" TF status: "inferred" TF family: "C2H2 ZF" DBDs: "zf-C2H2"]; Sp9[gene ID: "ENSMUSG00000068859" species: "Mus musculus" TF status: "inferred" TF family: "C2H2 ZF" DBDs: "zf-C2H2"]<br>Possible TFs: SP4	*5.26463*	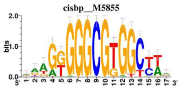	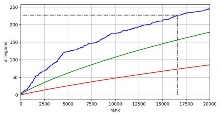	**link**	**link**	PWMs
26	☐ predrem__nrMotif1714<br>Description: 268_HCM-DS12599.M32	*5.24944*	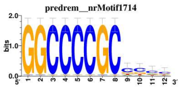	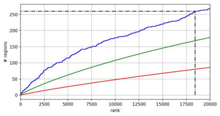	**link**	**link**	PWMs
27	☐ cisbp__M5210<br>Description: SP5[gene ID: "ENSG00000204335" species: "Homo sapiens" TF status: "inferred" TF family: "C2H2 ZF" DBDs: "zf-C2H2"]; SP6[gene ID: "ENSG00000189120" species: "Homo sapiens" TF status: "inferred" TF family: "C2H2 ZF" DBDs: "zf-C2H2"]; SP7[gene ID: "ENSG00000170374" species: "Homo sapiens" TF status: "inferred" TF family: "C2H2 ZF" DBDs: "zf-C2H2"]; SP9[gene ID: "ENSG00000217236" species: "Homo sapiens" TF status: "inferred" TF family: "C2H2 ZF" DBDs: "zf-C2H2"]; Sp1[gene ID: "FBgn0020378" species: "Drosophila melanogaster" TF status: "direct" TF family: "C2H2 ZF" DBDs: "zf-C2H2"]; Sp2[gene ID: "ENSMUSG00000018678" species: "Mus musculus" TF status: "inferred" TF family: "C2H2 ZF" DBDs: "zf-C2H2"]; Sp3[gene ID: "ENSMUSG00000027109" species: "Mus musculus" TF status: "inferred" TF family: "C2H2 ZF" DBDs: "zf-C2H2"]; Sp5[gene ID: "ENSMUSG00000075304" species: "Mus musculus" TF status: "inferred" TF family: "C2H2 ZF" DBDs: "zf-C2H2"]; Sp6[gene ID: "ENSMUSG00000038560" species: "Mus musculus" TF status: "inferred" TF family: "C2H2 ZF" DBDs: "zf-C2H2"]; Sp7[gene ID: "ENSMUSG00000060284" species: "Mus musculus" TF status: "inferred" TF family: "C2H2 ZF" DBDs: "zf-C2H2"]; Sp8[gene ID: "ENSMUSG00000048562" species: "Mus musculus" TF status: "inferred" TF family: "C2H2 ZF" DBDs: "zf-C2H2"]; Sp9[gene ID: "ENSMUSG00000068859" species: "Mus musculus" TF status: "inferred" TF family: "C2H2 ZF" DBDs: "zf-C2H2"]<br>Possible TFs: SP8, SP9, SP6, SP7	*5.20966*	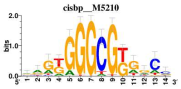	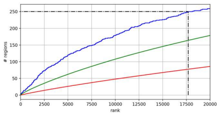	**link**	**link**	PWMs

#	Feature	NES	Logo	Recovery Curve	Candidate targets	All regions in top 20000	Database
28	☐ cisbp__M4870<br>Description: CG5669[gene ID: "FBgn0039169" species: "Drosophila melanogaster" TF status: "direct" TF family: "C2H2 ZF" DBDs: "zf-C2H2"]; SP5[gene ID: "ENSG00000204335" species: "Homo sapiens" TF status: "inferred" TF family: "C2H2 ZF" DBDs: "zf-C2H2"]; SP6[gene ID: "ENSG00000189120" species: "Homo sapiens" TF status: "inferred" TF family: "C2H2 ZF" DBDs: "zf-C2H2"]; SP7[gene ID: "ENSG00000170374" species: "Homo sapiens" TF status: "inferred" TF family: "C2H2 ZF" DBDs: "zf-C2H2"]; SP9[gene ID: "ENSG00000217236" species: "Homo sapiens" TF status: "inferred" TF family: "C2H2 ZF" DBDs: "zf-C2H2"]; Sp2[gene ID: "ENSMUSG00000018678" species: "Mus musculus" TF status: "inferred" TF family: "C2H2 ZF" DBDs: "zf-C2H2"]; Sp3[gene ID: "ENSMUSG00000027109" species: "Mus musculus" TF status: "inferred" TF family: "C2H2 ZF" DBDs: "zf-C2H2"]; Sp5[gene ID: "ENSMUSG00000075304" species: "Mus musculus" TF status: "inferred" TF family: "C2H2 ZF" DBDs: "zf-C2H2"]; Sp6[gene ID: "ENSMUSG00000038560" species: "Mus musculus" TF status: "inferred" TF family: "C2H2 ZF" DBDs: "zf-C2H2"]; Sp7[gene ID: "ENSMUSG00000060284" species: "Mus musculus" TF status: "inferred" TF family: "C2H2 ZF" DBDs: "zf-C2H2"]; Sp8[gene ID: "ENSMUSG00000048562" species: "Mus musculus" TF status: "inferred" TF family: "C2H2 ZF" DBDs: "zf-C2H2"]; Sp9[gene ID: "ENSMUSG00000068859" species: "Mus musculus" TF status: "inferred" TF family: "C2H2 ZF" DBDs: "zf-C2H2"]<br>Possible TFs: SP1, SP2, SP3, SP4	*5.17884*	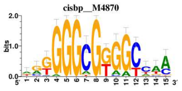	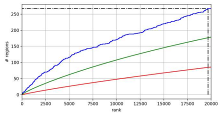	**link**	**link**	PWMs
29	☐ elemento__GCCCCGCCC<br>Description: Conserved regulatory element GCCCCGCCC between Hs and Mm	*4.97886*	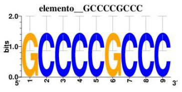	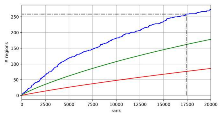	**link**	**link**	PWMs
30	☐ hocomoco__SP2_HUMAN.H11MO.0.A<br>Description: SP2_HUMAN<br>Possible TFs: SP2	*4.94066*	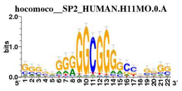	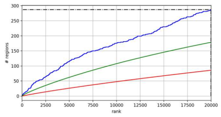	**link**	**link**	PWMs
31	☐ dbcorrdb__CREB1__ENCSR000BRB_1__m1<br>Description: CREB1 (ENCSR000BRB-1, motif 1)<br>Possible TFs: CREB1	*4.93186*	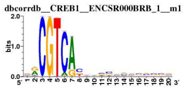	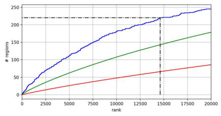	**link**	**link**	PWMs
32	☐ transfac_pro__M09005<br>Description: V$SP4_06: SP4<br>Possible TFs: SP4	*4.92933*	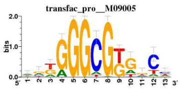	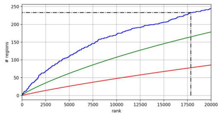	**link**	**link**	PWMs
33	☐ transfac_public__M00196<br>Description: V$SP1_Q6: Sp1<br>Possible TFs: SP1	*4.92523*	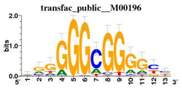	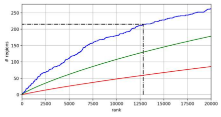	**link**	**link**	PWMs

#	Feature	NES	Logo	Recovery Curve	Candidate targets	All regions in top 20000	Database
34	☐ cisbp__M3926<br>Description: SP1[gene ID: "ENSG00000185591" species: "Homo sapiens" TF status: "direct" TF family: "C2H2 ZF" DBDs: "zf-C2H2"]; SP5[gene ID: "ENSG00000204335" species: "Homo sapiens" TF status: "inferred" TF family: "C2H2 ZF" DBDs: "zf-C2H2"]; SP6[gene ID: "ENSG00000189120" species: "Homo sapiens" TF status: "inferred" TF family: "C2H2 ZF" DBDs: "zf-C2H2"]; SP7[gene ID: "ENSG00000170374" species: "Homo sapiens" TF status: "inferred" TF family: "C2H2 ZF" DBDs: "zf-C2H2"]; SP9[gene ID: "ENSG00000217236" species: "Homo sapiens" TF status: "inferred" TF family: "C2H2 ZF" DBDs: "zf-C2H2"]; Sp2[gene ID: "ENSMUSG00000018678" species: "Mus musculus" TF status: "inferred" TF family: "C2H2 ZF" DBDs: "zf-C2H2"]; Sp3[gene ID: "ENSMUSG00000027109" species: "Mus musculus" TF status: "inferred" TF family: "C2H2 ZF" DBDs: "zf-C2H2"]; Sp5[gene ID: "ENSMUSG00000075304" species: "Mus musculus" TF status: "inferred" TF family: "C2H2 ZF" DBDs: "zf-C2H2"]; Sp6[gene ID: "ENSMUSG00000038560" species: "Mus musculus" TF status: "inferred" TF family: "C2H2 ZF" DBDs: "zf-C2H2"]; Sp7[gene ID: "ENSMUSG00000060284" species: "Mus musculus" TF status: "inferred" TF family: "C2H2 ZF" DBDs: "zf-C2H2"]; Sp8[gene ID: "ENSMUSG00000048562" species: "Mus musculus" TF status: "inferred" TF family: "C2H2 ZF" DBDs: "zf-C2H2"]; Sp9[gene ID: "ENSMUSG00000068859" species: "Mus musculus" TF status: "inferred" TF family: "C2H2 ZF" DBDs: "zf-C2H2"]<br>Possible TFs: SP1	*4.88175*	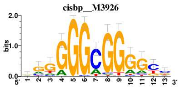	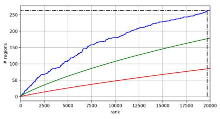	link	link	PWMs
35	☐ flyfactorsurvey__CG5669_SANGER_10_FBgn0039169<br>Description: CG5669_SANGER_10_FBgn0039169<br>Possible TFs: SP1, SP2, SP3, SP4	*4.87358*	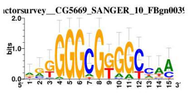	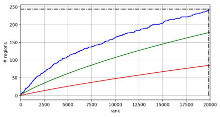	link	link	PWMs
36	☐ dbcorrdb__SP1__ENCSR000BKO_1__m2<br>Description: SP1 (ENCSR000BKO-1, motif 2)<br>Possible TFs: SP1	*4.85510*	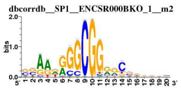	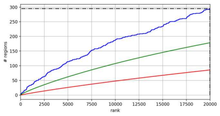	link	link	PWMs
37	☐ hocomoco__KLF9_HUMAN.H11MO.0.C<br>Description: KLF9_HUMAN<br>Possible TFs: KLF9	*4.62164*	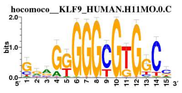	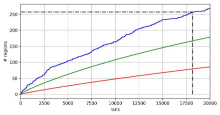	link	link	PWMs
38	☐ hocomoco__E2F4_MOUSE.H11MO.0.A<br>Description: E2F4_MOUSE<br>Possible TFs: E2F4	*4.50045*	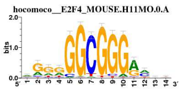	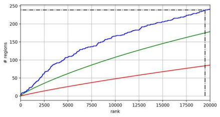	link	link	PWMs
39	☐ taipale_cyt_meth__SP3_NRCCMCGCCCMYN_eDBD<br>Description: SP3 [Znf_C2H2]<br>Possible TFs: SP3	*4.44174*	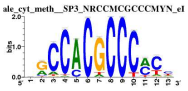	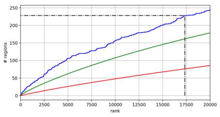	link	link	PWMs
40	☐ taipale_cyt_meth__KLF15_RCCACGCCCMYN_eDBD_meth<br>Description: KLF15 [Znf_C2H2, CpG-meth]<br>Possible TFs: KLF15	*4.43304*	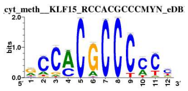	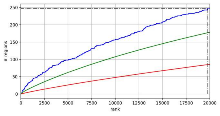	link	link	PWMs
41	☐ transfac_pro__M07615<br>Description: V$SP3_Q3_01: Sp3<br>Possible TFs: SP3	*4.42423*	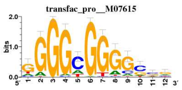	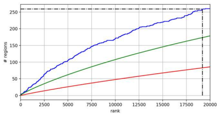	link	link	PWMs

#	Feature	NES	Logo	Recovery Curve	Candidate targets	All regions in top 20000	Database
42	☐ transfac_pro__M05319<br>Description: V$KLF7_05: KLF7<br>Possible TFs: KLF7	*4.42198*	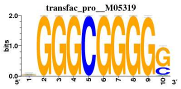	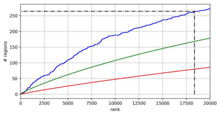	link	link	PWMs
43	☐ transfac_pro__M01861<br>Description: V$ATF1_Q6_01: ATF-1<br>Possible TFs: ATF1	*4.40866*	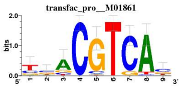	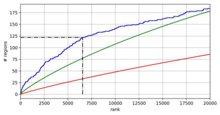	link	link	PWMs
44	☐ taipale__KLF14_DBD_NGCCACGCCCMCNT<br>Description: KLF14 monomeric C2H2 DBD<br>Possible TFs: KLF14	*4.39668*	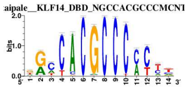	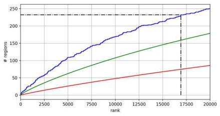	link	link	PWMs
45	☐ cisbp__M4837<br>Description: CG3065[gene ID: "FBgn0034946" species: "Drosophila melanogaster" TF status: "direct" TF family: "C2H2 ZF" DBDs: "zf-C2H2"]<br>Possible TFs: KLF18, KLF17	*4.39359*	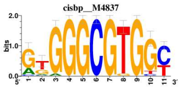	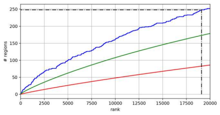	link	link	PWMs
46	☐ dbcorrdb__BRCA1__ENCSR000EDB_1__m1<br>Description: BRCA1 (ENCSR000EDB-1, motif 1)<br>Possible TFs: BRCA1	*4.38225*	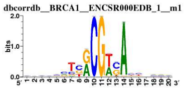	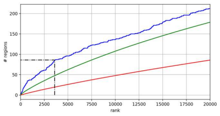	link	link	PWMs
47	☐ transfac_pro__M05318<br>Description: V$KLF8_01: KLF8<br>Possible TFs: KLF8	*4.35375*	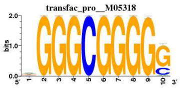	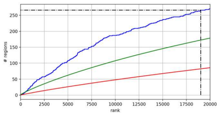	link	link	PWMs
48	☐ hocomoco__SP2_HUMAN.H11MO.1.B<br>Description: SP2_HUMAN<br>Possible TFs: SP2	*4.33349*	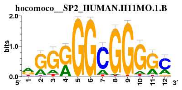	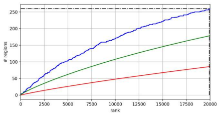	link	link	PWMs
49	☐ transfac_pro__M05437<br>Description: V$KLF7_06: Klf7<br>Possible TFs: KLF7	*4.32844*	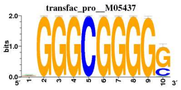	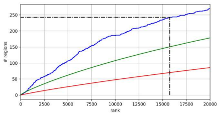	link	link	PWMs
50	☐ flyfactorsurvey__CG3065_F1-5_SOLEXA_2.5_FBgn0034946<br>Description: CG3065_F1-5_SOLEXA_2.5_FBgn0034946<br>Possible TFs: KLF18, KLF17	*4.21087*	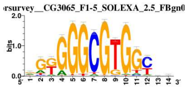	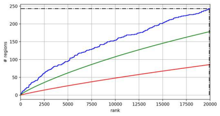	link	link	PWMs
51	☐ taipale_cyt_meth__SP1_NWRGCCACGCCCMYN_eDBD_meth<br>Description: SP1 [Znf_C2H2, CpG-meth]<br>Possible TFs: SP1	*4.19607*	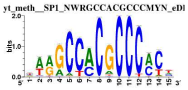	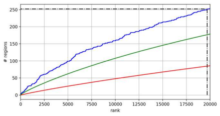	link	link	PWMs
52	☐ taipale_cyt_meth__CREB1_NRTGACGTN_eDBD_repr<br>Description: CREB1 [bZIP]<br>Possible TFs: CREB1	*4.18973*	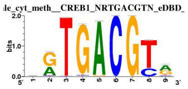	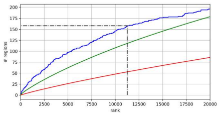	link	link	PWMs
53	☐ transfac_pro__M04687<br>Description: V$BRCA1_02: BRCA1<br>Possible TFs: BRCA1	*4.16645*	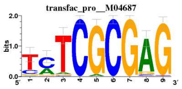	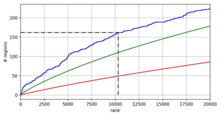	link	link	PWMs
54	☐ transfac_pro__M00931<br>Description: V$SP1_Q6_01: Sp1<br>Possible TFs: SP1, SP3, SP4	*4.16142*	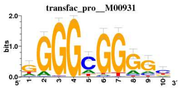	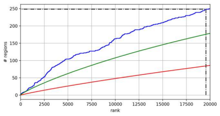	link	link	PWMs

#	Feature	NES	Logo	Recovery Curve	Candidate targets	All regions in top 20000	Database
55	☐ transfac_pro__M05555<br>Description: V$SP5_02: Sp5<br>Possible TFs: SP5	*4.15020*	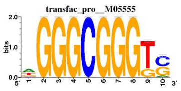	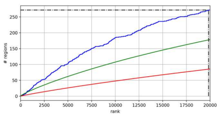	link	link	PWMs
56	☐ transfac_pro__M00932<br>Description: V$SP1_Q4_01: Sp1<br>Possible TFs: SP1, SP2, SP3, SP4	*4.14446*	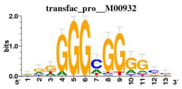	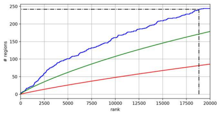	link	link	PWMs
57	☐ cisbp__M6180<br>Description: CREB1[gene ID: "ENSG00000118260" species: "Homo sapiens" TF status: "direct" TF family: "bZIP" DBDs: "bZIP_1"]; CrebB-17A[gene ID: "FBgn0014467" species: "Drosophila melanogaster" TF status: "inferred" TF family: "bZIP" DBDs: "bZIP_1"]<br>Possible TFs: CREB1	*4.13680*	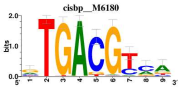	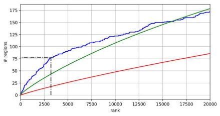	link	link	PWMs
58	☐ transfac_pro__M05552<br>Description: V$SP5_01: SP5<br>Possible TFs: SP5	*4.09022*	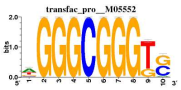	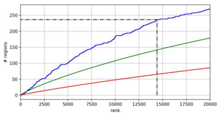	link	link	PWMs
59	☐ elemento__CCAATCG<br>Description: Conserved regulatory element CCAATCG between Hs and Mm	*4.08872*	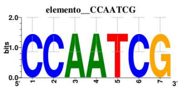	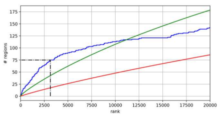	link	link	PWMs
60	☐ dbcorrdb__E2F4__ENCSR000DYY_1__m2<br>Description: E2F4 (ENCSR000DYY-1, motif 2)<br>Possible TFs: E2F4	*4.08690*	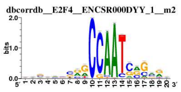	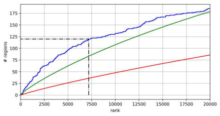	link	link	PWMs
61	☐ transfac_public__M00255<br>Description: V$GC_01: GC box	*4.06845*	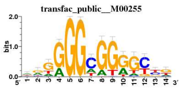	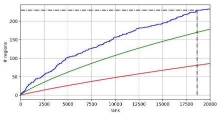	link	link	PWMs
62	☐ cisbp__M5592<br>Description: KLF14[gene ID: "ENSG00000174595" species: "Homo sapiens" TF status: "direct" TF family: "C2H2 ZF" DBDs: "zf-C2H2"]; KLF9[gene ID: "ENSG00000119138" species: "Homo sapiens" TF status: "inferred" TF family: "C2H2 ZF" DBDs: "zf-C2H2"]; Klf11[gene ID: "ENSMUSG00000020653" species: "Mus musculus" TF status: "inferred" TF family: "C2H2 ZF" DBDs: "zf-C2H2"]; Klf13[gene ID: "ENSMUSG00000052040" species: "Mus musculus" TF status: "inferred" TF family: "C2H2 ZF" DBDs: "zf-C2H2"]; Klf14[gene ID: "ENSMUSG00000073209" species: "Mus musculus" TF status: "inferred" TF family: "C2H2 ZF" DBDs: "zf-C2H2"]	*4.05783*	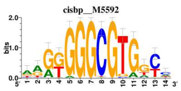	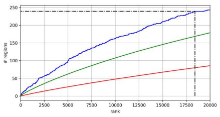	link	link	PWMs
63	☐ E086-DNase.hotspot.all.peaks-narrowpeak<br>Description: DNase in Fetal Kidney (E086, narrowpeak, hotspot)	*4.04578*		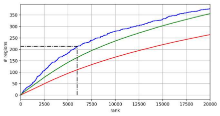	link	link	DHS & FAIRE
64	☐ transfac_pro__M05493<br>Description: V$SP9_01: SP9<br>Possible TFs: SP9	*4.03623*	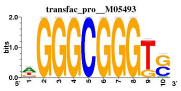	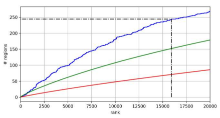	link	link	PWMs
65	☐ cisbp__M2247<br>Description: btd[gene ID: "FBgn0000233" species: "Drosophila melanogaster" TF status: "direct" TF family: "C2H2 ZF" DBDs: "zf-C2H2"]<br>Possible TFs: SP1, SP2, SP3, SP4	*4.02771*	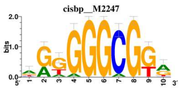	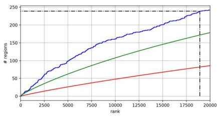	link	link	PWMs

#	Feature	NES	Logo	Recovery Curve	Candidate targets	All regions in top 20000	Database
66	☐ transfac_pro__M03807<br>Description: V$SP2_Q3_01: SP2<br>Possible TFs: SP2	*4.02549*	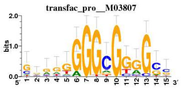	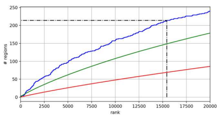	link	link	PWMs
67	☐ cisbp__M4839<br>Description: CG3065[gene ID: "FBgn0034946" species: "Drosophila melanogaster" TF status: "direct" TF family: "C2H2 ZF" DBDs: "zf-C2H2"]<br>Possible TFs: KLF18, KLF17	*4.02240*	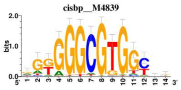	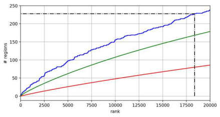	link	link	PWMs
68	☐ transfac_pro__M04797<br>Description: V$EGR1_09: Egr-1<br>Possible TFs: EGR1	*4.02004*	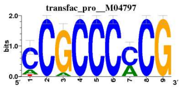	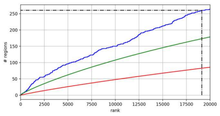	link	link	PWMs
69	☐ cisbp__M4782<br>Description: btd[gene ID: "FBgn0000233" species: "Drosophila melanogaster" TF status: "direct" TF family: "C2H2 ZF" DBDs: "zf-C2H2"]<br>Possible TFs: SP1, SP2, SP3, SP4	*4.01745*	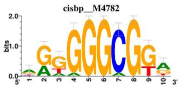	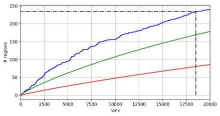	link	link	PWMs
70	☐ transfac_pro__M05553<br>Description: V$SP8_02: SP8<br>Possible TFs: SP8	*4.01739*	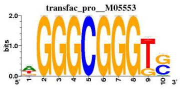	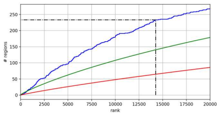	link	link	PWMs
71	☐ predrem__nrMotif583<br>Description: 154_fLung_R-DS17162.M642	*4.01023*	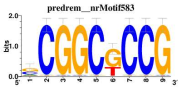	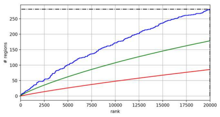	link	link	PWMs
72	☐ hocomoco__KLF14_HUMAN.H11MO.0.D<br>Description: KLF14_HUMAN	*3.99032*	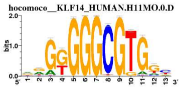	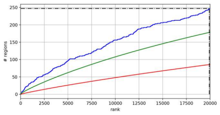	link	link	PWMs
73	☐ cisbp__M1748<br>Description: M1748	*3.98708*	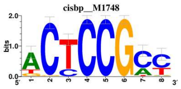	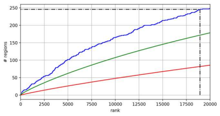	link	link	PWMs
74	☐ jaspar__MA1034.1<br>Description: Os05g0497200	*3.98676*	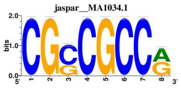	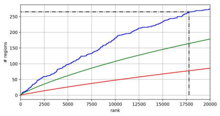	link	link	PWMs
75	☐ dbcorrdb__POLR2A__ENCSR000BHI_1__m1<br>Description: POLR2A (ENCSR000BHI-1, motif 1)<br>Possible TFs: POLR2A	*3.98593*	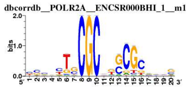	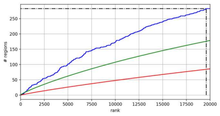	link	link	PWMs
76	☐ dbcorrdb__POLR2A__ENCSR000DNF_1__m1<br>Description: POLR2A (ENCSR000DNF-1, motif 1)<br>Possible TFs: POLR2A	*3.98585*	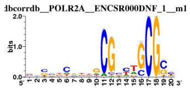	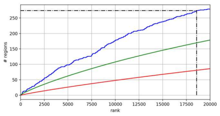	link	link	PWMs
77	☐ dbcorrdb__E2F4__ENCSR000DOR_1__m2<br>Description: E2F4 (ENCSR000DOR-1, motif 2)<br>Possible TFs: E2F4	*3.98419*	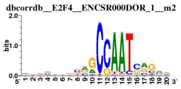	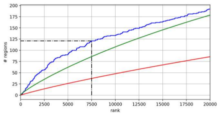	link	link	PWMs
78	☐ cisbp__M0043<br>Description: M0043	*3.98230*	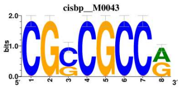	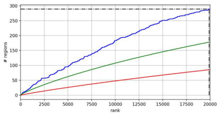	link	link	PWMs

#	Feature	NES	Logo	Recovery Curve	Candidate targets	All regions in top 20000	Database
79	☐ predrem__nrMotif258<br>Description: 261_HAc-DS14765.M101	3.97119	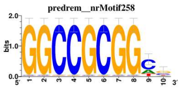	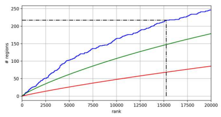	link	link	PWMs
80	☐ dbcorrdb__CHD2__ENCSR000EBT_1__m1<br>Description: CHD2 (ENCSR000EBT-1, motif 1)<br>Possible TFs: CHD2	3.96605	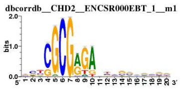	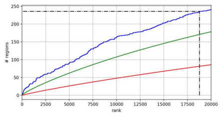	link	link	PWMs
81	☐ dbcorrdb__ATF3__ENCSR000BJY_1__m1<br>Description: ATF3 (ENCSR000BJY-1, motif 1)<br>Possible TFs: ATF3	3.96120	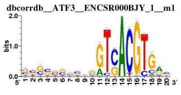	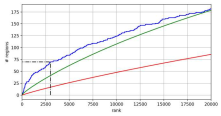	link	link	PWMs
82	☐ dbcorrdb__FOS__ENCSR000EYZ_1__m1<br>Description: FOS (ENCSR000EYZ-1, motif 1)<br>Possible TFs: FOS	3.95296	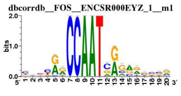	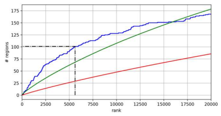	link	link	PWMs
83	☐ transfac_pro__M07395<br>Description: V$SP1_Q2_02: Sp1<br>Possible TFs: SP1	3.94406	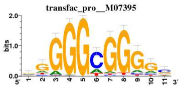	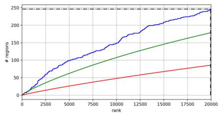	link	link	PWMs
84	☐ jaspar__MA0516.1<br>Description: SP2<br>Possible TFs: SP2	3.93293	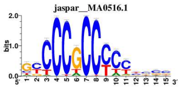	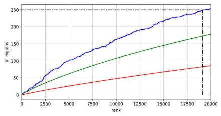	link	link	PWMs
85	☐ ENCFF001WEP<br>Description: DNase-seq on human CD20+ (RO01778)	3.92383		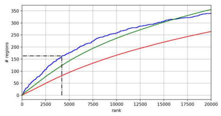	link	link	DHS & FAIRE
86	☐ ENCFF001WIW<br>Description: DNase-seq on human HepG2	3.92357		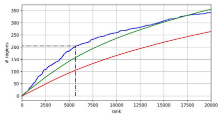	link	link	DHS & FAIRE
87	☐ taipale_cyt_meth__SP8_NCCACGCCCMYN_eDBD<br>Description: SP8 [Znf_C2H2]<br>Possible TFs: SP8	3.91954	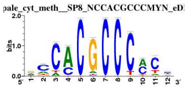	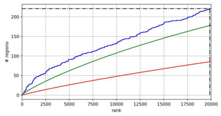	link	link	PWMs
88	☐ predrem__nrMotif1136<br>Description: 196_fMuscle_leg-DS19272.M145	3.90649	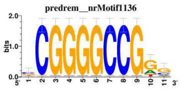	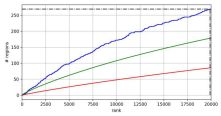	link	link	PWMs
89	☐ jaspar__MA0535.1<br>Description: Mad<br>Possible TFs: SMAD5, SMAD1, SMAD9	3.90585	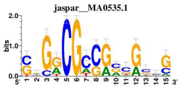	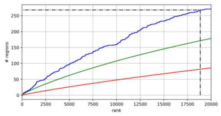	link	link	PWMs
90	☐ ENCFF001VYW<br>Description: DNase-seq on human CD20+ (RO01778)	3.90459		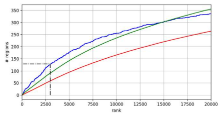	link	link	DHS & FAIRE
91	☐ hocomoco__SP1_MOUSE.H11MO.1.A<br>Description: SP1_MOUSE<br>Possible TFs: SP1	3.90056	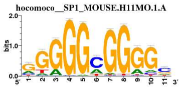	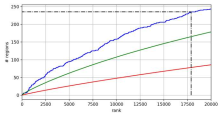	link	link	PWMs

#	Feature	NES	Logo	Recovery Curve	Candidate targets	All regions in top 20000	Database
92	☐ cisbp__M2329<br>Description: Mad[gene ID: "FBgn0011648" species: "Drosophila melanogaster" TF status: "direct" TF family: "SMAD" DBDs: "MH1"]; Smad1[gene ID: "ENSMUSG00000031681" species: "Mus musculus" TF status: "inferred" TF family: "SMAD" DBDs: "MH1"]<br>Possible TFs: SMAD5, SMAD1, SMAD9	*3.89641*	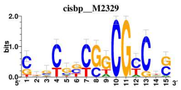	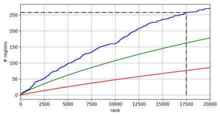	link	link	PWMs
93	☐ dbcorrdb__NFYB__ENCSR000DNR_1__m1<br>Description: NFYB (ENCSR000DNR-1, motif 1)<br>Possible TFs: NFYB	*3.88548*	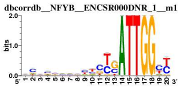	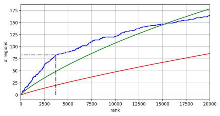	link	link	PWMs
94	☐ dbcorrdb__SP1__ENCSR000BHK_1__m1<br>Description: SP1 (ENCSR000BHK-1, motif 1)<br>Possible TFs: SP1	*3.88283*	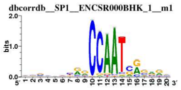	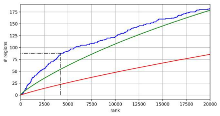	link	link	PWMs
95	☐ transfac_pro__M05066<br>Description: P$ERF1_05: ERF1(2)	*3.87716*	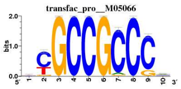	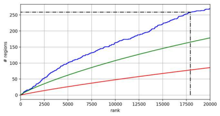	link	link	PWMs
96	☐ cisbp__M5209<br>Description: SP5[gene ID: "ENSG00000204335" species: "Homo sapiens" TF status: "inferred" TF family: "C2H2 ZF" DBDs: "zf-C2H2"]; SP6[gene ID: "ENSG00000189120" species: "Homo sapiens" TF status: "inferred" TF family: "C2H2 ZF" DBDs: "zf-C2H2"]; SP7[gene ID: "ENSG00000170374" species: "Homo sapiens" TF status: "inferred" TF family: "C2H2 ZF" DBDs: "zf-C2H2"]; SP9[gene ID: "ENSG00000217236" species: "Homo sapiens" TF status: "inferred" TF family: "C2H2 ZF" DBDs: "zf-C2H2"]; Sp1[gene ID: "FBgn0020378" species: "Drosophila melanogaster" TF status: "direct" TF family: "C2H2 ZF" DBDs: "zf-C2H2"]; Sp2[gene ID: "ENSMUSG00000018678" species: "Mus musculus" TF status: "inferred" TF family: "C2H2 ZF" DBDs: "zf-C2H2"]; Sp3[gene ID: "ENSMUSG00000027109" species: "Mus musculus" TF status: "inferred" TF family: "C2H2 ZF" DBDs: "zf-C2H2"]; Sp5[gene ID: "ENSMUSG00000075304" species: "Mus musculus" TF status: "inferred" TF family: "C2H2 ZF" DBDs: "zf-C2H2"]; Sp6[gene ID: "ENSMUSG00000038560" species: "Mus musculus" TF status: "inferred" TF family: "C2H2 ZF" DBDs: "zf-C2H2"]; Sp7[gene ID: "ENSMUSG00000060284" species: "Mus musculus" TF status: "inferred" TF family: "C2H2 ZF" DBDs: "zf-C2H2"]; Sp8[gene ID: "ENSMUSG00000048562" species: "Mus musculus" TF status: "inferred" TF family: "C2H2 ZF" DBDs: "zf-C2H2"]; Sp9[gene ID: "ENSMUSG00000068859" species: "Mus musculus" TF status: "inferred" TF family: "C2H2 ZF" DBDs: "zf-C2H2"]<br>Possible TFs: SP8, SP9, SP6, SP7	*3.87647*	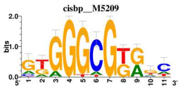	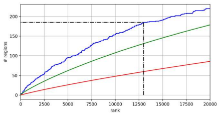	link	link	PWMs
97	☐ dbcorrdb__NFYA__ENCSR000DNS_1__m1<br>Description: NFYA (ENCSR000DNS-1, motif 1)<br>Possible TFs: NFYA	*3.87503*	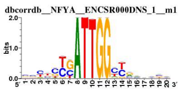	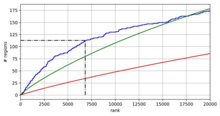	link	link	PWMs
98	☐ ENCFF001VZW<br>Description: DNase-seq on human HepG2	*3.86639*		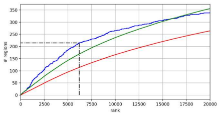	link	link	DHS & FAIRE

#	Feature	NES	Logo	Recovery Curve	Candidate targets	All regions in top 20000	Database
99	☐ cisbp__M2314<br>Description: SP2[gene ID: "ENSG00000167182" species: "Homo sapiens" TF status: "direct" TF family: "C2H2 ZF" DBDs: "zf-C2H2"]; SP5[gene ID: "ENSG00000204335" species: "Homo sapiens" TF status: "inferred" TF family: "C2H2 ZF" DBDs: "zf-C2H2"]; SP6[gene ID: "ENSG00000189120" species: "Homo sapiens" TF status: "inferred" TF family: "C2H2 ZF" DBDs: "zf-C2H2"]; SP7[gene ID: "ENSG00000170374" species: "Homo sapiens" TF status: "inferred" TF family: "C2H2 ZF" DBDs: "zf-C2H2"]; SP9[gene ID: "ENSG00000217236" species: "Homo sapiens" TF status: "inferred" TF family: "C2H2 ZF" DBDs: "zf-C2H2"]; Sp2[gene ID: "ENSMUSG00000018678" species: "Mus musculus" TF status: "inferred" TF family: "C2H2 ZF" DBDs: "zf-C2H2"]; Sp3[gene ID: "ENSMUSG00000027109" species: "Mus musculus" TF status: "inferred" TF family: "C2H2 ZF" DBDs: "zf-C2H2"]; Sp5[gene ID: "ENSMUSG00000075304" species: "Mus musculus" TF status: "inferred" TF family: "C2H2 ZF" DBDs: "zf-C2H2"]; Sp6[gene ID: "ENSMUSG00000038560" species: "Mus musculus" TF status: "inferred" TF family: "C2H2 ZF" DBDs: "zf-C2H2"]; Sp7[gene ID: "ENSMUSG00000060284" species: "Mus musculus" TF status: "inferred" TF family: "C2H2 ZF" DBDs: "zf-C2H2"]; Sp8[gene ID: "ENSMUSG00000048562" species: "Mus musculus" TF status: "inferred" TF family: "C2H2 ZF" DBDs: "zf-C2H2"]; Sp9[gene ID: "ENSMUSG00000068859" species: "Mus musculus" TF status: "inferred" TF family: "C2H2 ZF" DBDs: "zf-C2H2"]<br>Possible TFs: SP2	*3.86575*	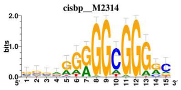	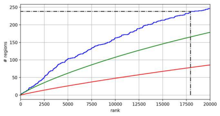	**link**	**link**	PWMs
100	☐ dbcorrdb__CHD2__ENCSR000EED_1__m1<br>Description: CHD2 (ENCSR000EED-1, motif 1)<br>Possible TFs: CHD2	*3.86472*	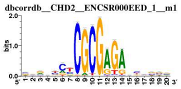	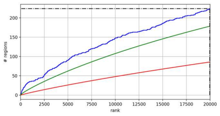	**link**	**link**	PWMs
101	☐ predrem__nrMotif374<br>Description: 304_hTH1-DS7840.M360	*3.84494*	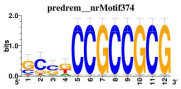	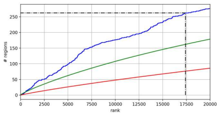	**link**	**link**	PWMs
102	☐ dbcorrdb__POLR2A__ENCSR000DMZ_1__m2<br>Description: POLR2A (ENCSR000DMZ-1, motif 2)<br>Possible TFs: POLR2A	*3.82615*	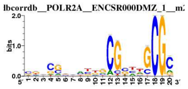	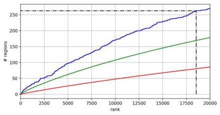	**link**	**link**	PWMs
103	☐ elemento__CGCCGCG<br>Description: Conserved regulatory element CGCCGCG between Hs and Mm	*3.81802*	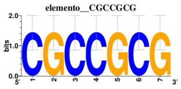	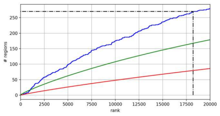	**link**	**link**	PWMs

#	Feature	NES	Logo	Recovery Curve	Candidate targets	All regions in top 20000	Database
104	☐ cisbp__M6479<br>Description: SP1[gene ID: "ENSG00000185591" species: "Homo sapiens" TF status: "direct" TF family: "C2H2 ZF" DBDs: "zf-C2H2"]; SP5[gene ID: "ENSG00000204335" species: "Homo sapiens" TF status: "inferred" TF family: "C2H2 ZF" DBDs: "zf-C2H2"]; SP6[gene ID: "ENSG00000189120" species: "Homo sapiens" TF status: "inferred" TF family: "C2H2 ZF" DBDs: "zf-C2H2"]; SP7[gene ID: "ENSG00000170374" species: "Homo sapiens" TF status: "inferred" TF family: "C2H2 ZF" DBDs: "zf-C2H2"]; SP9[gene ID: "ENSG00000217236" species: "Homo sapiens" TF status: "inferred" TF family: "C2H2 ZF" DBDs: "zf-C2H2"]; Sp2[gene ID: "ENSMUSG00000018678" species: "Mus musculus" TF status: "inferred" TF family: "C2H2 ZF" DBDs: "zf-C2H2"]; Sp3[gene ID: "ENSMUSG00000027109" species: "Mus musculus" TF status: "inferred" TF family: "C2H2 ZF" DBDs: "zf-C2H2"]; Sp5[gene ID: "ENSMUSG00000075304" species: "Mus musculus" TF status: "inferred" TF family: "C2H2 ZF" DBDs: "zf-C2H2"]; Sp6[gene ID: "ENSMUSG00000038560" species: "Mus musculus" TF status: "inferred" TF family: "C2H2 ZF" DBDs: "zf-C2H2"]; Sp7[gene ID: "ENSMUSG00000060284" species: "Mus musculus" TF status: "inferred" TF family: "C2H2 ZF" DBDs: "zf-C2H2"]; Sp8[gene ID: "ENSMUSG00000048562" species: "Mus musculus" TF status: "inferred" TF family: "C2H2 ZF" DBDs: "zf-C2H2"]; Sp9[gene ID: "ENSMUSG00000068859" species: "Mus musculus" TF status: "inferred" TF family: "C2H2 ZF" DBDs: "zf-C2H2"]<br>Possible TFs: SP1	*3.81323*	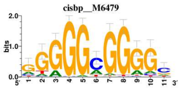	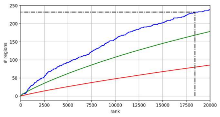	**link**	**link**	PWMs
105	☐ transfac_pro__M00375<br>Description: P$TGA1B_Q2: TGA1b	*3.80934*	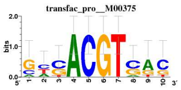	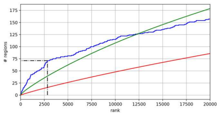	**link**	**link**	PWMs
106	☐ dbcorrdb__POLR2A__ENCSR000BHH_1__m1<br>Description: POLR2A (ENCSR000BHH-1, motif 1)<br>Possible TFs: POLR2A	*3.80917*	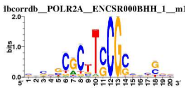	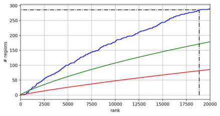	**link**	**link**	PWMs
107	☐ cisbp__M4597<br>Description: ATF3[gene ID: "ENSG00000162772" species: "Homo sapiens" TF status: "direct" TF family: "bZIP" DBDs: "bZIP_1"]<br>Possible TFs: ATF3	*3.80594*	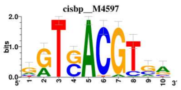	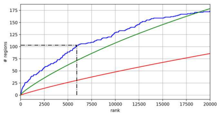	**link**	**link**	PWMs
108	☐ ENCFF001WEM<br>Description: DNase-seq on human Caco-2	*3.80360*		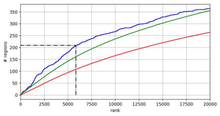	**link**	**link**	DHS & FAIRE
109	☐ dbcorrdb__PBX3__ENCSR000BGR_1__m2<br>Description: PBX3 (ENCSR000BGR-1, motif 2)<br>Possible TFs: PBX3	*3.80142*	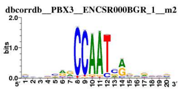	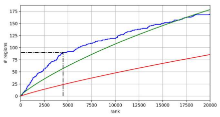	**link**	**link**	PWMs
110	☐ transfac_pro__M07063<br>Description: V$SP1_Q3: Sp1<br>Possible TFs: SP1	*3.79268*	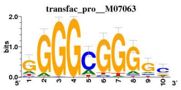	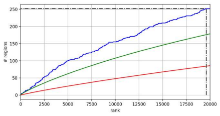	**link**	**link**	PWMs
111	☐ dbcorrdb__SREBF1__ENCSR000EEO_1__m2<br>Description: SREBF1 (ENCSR000EEO-1, motif 2)<br>Possible TFs: SREBF1	*3.79158*	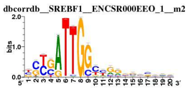	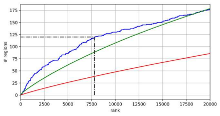	**link**	**link**	PWMs

#	Feature	NES	Logo	Recovery Curve	Candidate targets	All regions in top 20000	Database
112	☐ swissregulon__hs__ATF5_CREB3.p2<br>Description: hs__ATF5_CREB3.p2<br>Possible TFs: ATF5, CREB3	*3.78934*	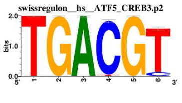	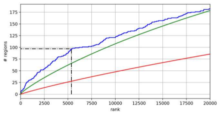	link	link	PWMs
113	☐ dbcorrdb__STAT1__ENCSR000DZM_1__m1<br>Description: STAT1 (ENCSR000DZM-1, motif 1)<br>Possible TFs: STAT1	*3.78557*	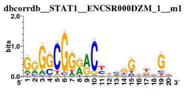	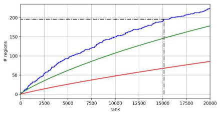	link	link	PWMs
114	☐ transfac_pro__M05438<br>Description: V$OSX_01: Osx<br>Possible TFs: SP7	*3.78229*	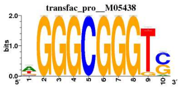	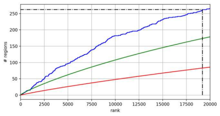	link	link	PWMs
115	☐ transfac_pro__M05554<br>Description: V$OSX_02: Osx<br>Possible TFs: SP7	*3.77231*	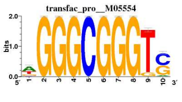	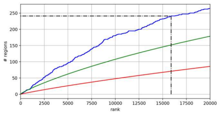	link	link	PWMs
116	☐ transfac_pro__M05040<br>Description: P$ERF2_04: ERF2 (2)	*3.77051*	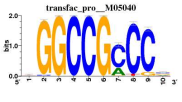	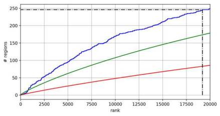	link	link	PWMs
117	☐ ENCFF001WCI<br>Description: DNase-seq on human SK-N-SH treated with 6uM retinoic acid for 48 hours	*3.75690*		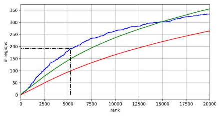	link	link	DHS & FAIRE
118	☐ transfac_pro__M05045<br>Description: P$DREB2C_01: DREB2C	*3.74969*	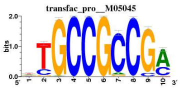	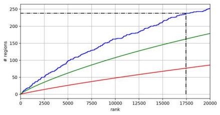	link	link	PWMs
119	☐ dbcorrdb__POLR2A__ENCSR000EEP_1__m1<br>Description: POLR2A (ENCSR000EEP-1, motif 1)<br>Possible TFs: POLR2A	*3.74333*	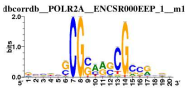	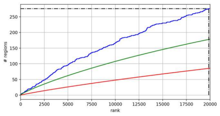	link	link	PWMs
120	☐ transfac_pro__M05042<br>Description: P$ERF3_03: ERF3	*3.74167*	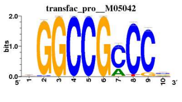	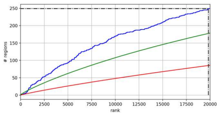	link	link	PWMs
121	☐ dbcorrdb__ATF3__ENCSR000BKC_1__m2<br>Description: ATF3 (ENCSR000BKC-1, motif 2)<br>Possible TFs: ATF3	*3.73556*	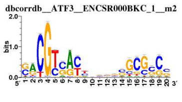	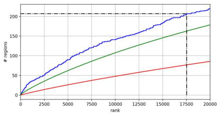	link	link	PWMs
122	☐ predrem__nrMotif1018<br>Description: 164_fMuscle_arm-DS18080.M145	*3.73346*	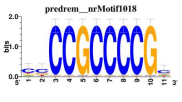	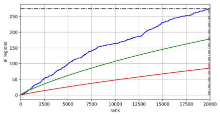	link	link	PWMs
123	☐ dbcorrdb__BRCA1__ENCSR000EBX_1__m1<br>Description: BRCA1 (ENCSR000EBX-1, motif 1)<br>Possible TFs: BRCA1	*3.73107*	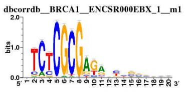	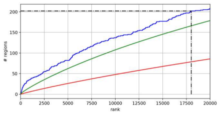	link	link	PWMs
124	☐ transfac_pro__M05012<br>Description: P$ERF3_02: ERF3	*3.72430*	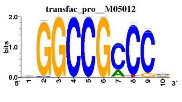	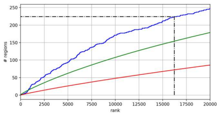	link	link	PWMs

#	Feature	NES	Logo	Recovery Curve	Candidate targets	All regions in top 20000	Database
125	☐ dbcorrdb__NFYB__ENCSR000EGQ_1__m1<br>Description: NFYB (ENCSR000EGQ-1, motif 1)<br>Possible TFs: NFYB	*3.71655*	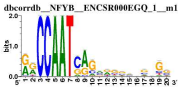	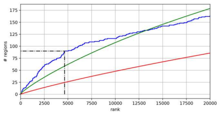	link	link	PWMs
126	☐ predrem__nrMotif1831<br>Description: 343_SKNSH-DS8482.M638	*3.71500*	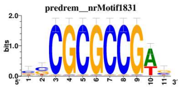	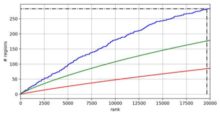	link	link	PWMs
127	☐ transfac_pro__M05169<br>Description: P$RRTF1_01: RRTF1	*3.70580*	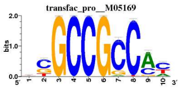	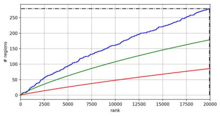	link	link	PWMs
128	☐ dbcorrdb__E2F4__ENCSR000EVL_1__m2<br>Description: E2F4 (ENCSR000EVL-1, motif 2)<br>Possible TFs: E2F4	*3.70567*	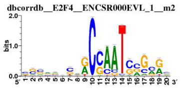	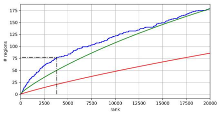	link	link	PWMs
129	☐ transfac_pro__M03567<br>Description: V$SP2_Q3: Sp2<br>Possible TFs: SP2	*3.70350*	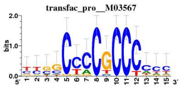	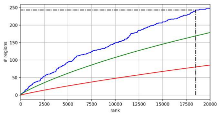	link	link	PWMs
130	☐ dbcorrdb__BRCA1__ENCSR000EDY_1__m1<br>Description: BRCA1 (ENCSR000EDY-1, motif 1)<br>Possible TFs: BRCA1	*3.69946*	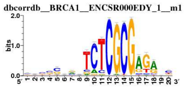	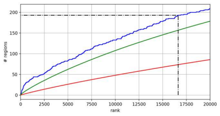	link	link	PWMs
131	☐ jaspar__MA0443.1<br>Description: btd<br>Possible TFs: SP1, SP2, SP3, SP4	*3.69544*	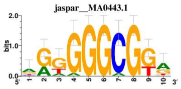	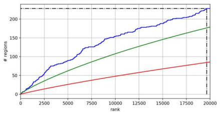	link	link	PWMs
132	☐ dbcorrdb__NFYA__ENCSR000EGR_1__m1<br>Description: NFYA (ENCSR000EGR-1, motif 1)<br>Possible TFs: NFYA	*3.69360*	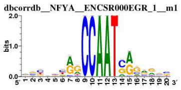	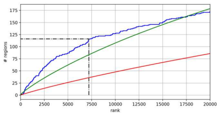	link	link	PWMs
133	☐ transfac_pro__M05311<br>Description: V$TIEG1_01: TIEG-1<br>Possible TFs: KLF10	*3.68152*	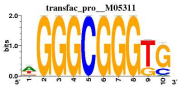	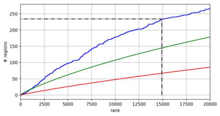	link	link	PWMs
134	☐ taipale_cyt_meth__SP3_NRCCMCGCCCMYN_eDBD_meth<br>Description: SP3 [Znf_C2H2, CpG-meth]<br>Possible TFs: SP3	*3.68081*	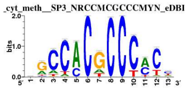	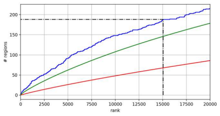	link	link	PWMs
135	☐ transfac_pro__M05067<br>Description: P$AT5G25190_01: At5g25190	*3.67603*	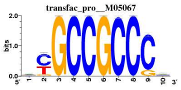	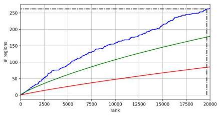	link	link	PWMs
136	☐ dbcorrdb__EZH2__ENCSR000AQE_1__m9<br>Description: EZH2 (ENCSR000AQE-1, motif 9)<br>Possible TFs: EZH2	*3.67581*	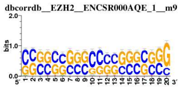	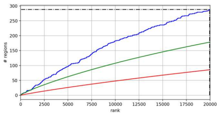	link	link	PWMs
137	☐ ENCFF001SQC<br>Description: DNase-seq on human Caco-2	*3.67002*		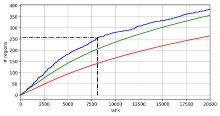	link	link	DHS & FAIRE

#	Feature	NES	Logo	Recovery Curve	Candidate targets	All regions in top 20000	Database
138	☐ transfac_pro__M06796<br>Description: V$BTEB1_01: BTEB1	*3.66785*	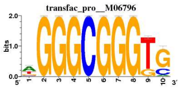	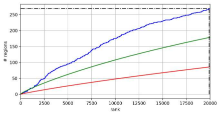	**link**	**link**	PWMs
139	☐ cisbp__M0270<br>Description: M0270	*3.66716*	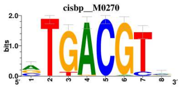	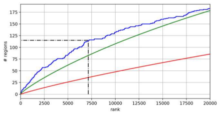	**link**	**link**	PWMs
140	☐ elemento__CGTCACG<br>Description: Conserved regulatory element CGTCACG between Hs and Mm	*3.66591*	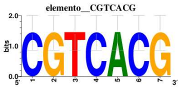	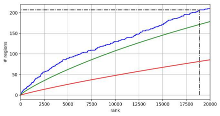	**link**	**link**	PWMs
141	☐ ENCFF001WEL<br>Description: DNase-seq on human Caco-2	*3.66431*		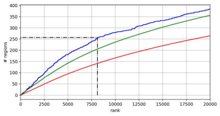	**link**	**link**	DHS & FAIRE
142	☐ ENCFF001WGI<br>Description: DNase-seq on human H7-hESC differentiated for 9 days	*3.65360*		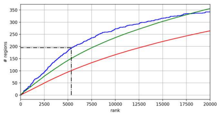	**link**	**link**	DHS & FAIRE
143	☐ taipale_cyt_meth__SP9_NCCACGCCCMYN_eDBD<br>Description: SP9 [ZZnf_C2H2]<br>Possible TFs: SP9	*3.65275*	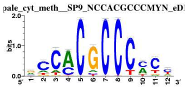	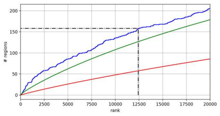	**link**	**link**	PWMs
144	☐ flyfactorsurvey__btd_NAR_FBgn0000233<br>Description: btd_NAR_FBgn0000233<br>Possible TFs: SP1, SP2, SP3, SP4	*3.64724*	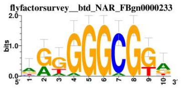	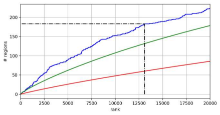	**link**	**link**	PWMs
145	☐ E125-H4K20me1-broadpeak<br>Description: H4K20me1 in NH-A Astrocytes Primary Cells (E125, broadpeak)	*3.64668*		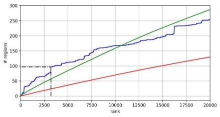	**link**	**link**	Histone modifications
146	☐ ENCFF001SSI<br>Description: DNase-seq on human SK-N-SH treated with 6uM retinoic acid for 48 hours	*3.64545*		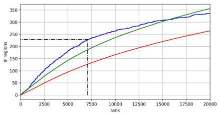	**link**	**link**	DHS & FAIRE
147	☐ ENCFF001WSU<br>Description: DNase-seq on human SK-N-SH treated with 6uM retinoic acid for 48 hours	*3.64393*		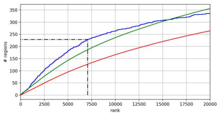	**link**	**link**	DHS & FAIRE
148	☐ cisbp__M0088<br>Description: M0088	*3.63738*	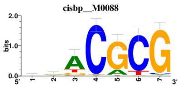	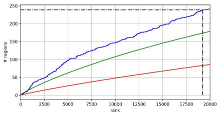	**link**	**link**	PWMs
149	☐ transfac_pro__M05312<br>Description: V$FKLF_01: FKLF<br>Possible TFs: KLF11	*3.63556*	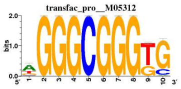	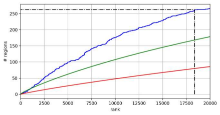	**link**	**link**	PWMs
150	☐ hdpi__RBM17<br>Description: RBM17<br>Possible TFs: RBM17	*3.63485*	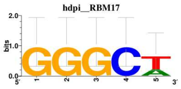	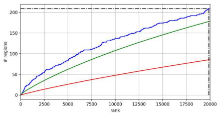	**link**	**link**	PWMs

#	Feature	NES	Logo	Recovery Curve	Candidate targets	All regions in top 20000	Database
151	☐ jaspar__MA1053.1<br>Description: ERF109	*3.63406*	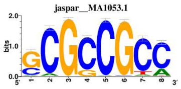	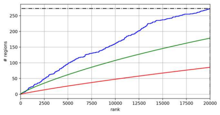	**link**	**link**	PWMs
152	☐ ENCFF001VZI<br>Description: DNase-seq on human HA-h	*3.63144*		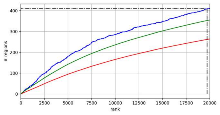	**link**	**link**	DHS & FAIRE
153	☐ flyfactorsurvey__Sp1_SANGER_5_FBgn0020378<br>Description: Sp1_SANGER_5_FBgn0020378<br>Possible TFs: SP8, SP9, SP6, SP7	*3.63077*	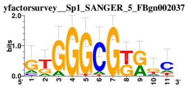	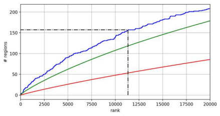	**link**	**link**	PWMs
154	☐ dbcorrdb__MXI1__ENCSR000EIA_1__m4<br>Description: MXI1 (ENCSR000EIA-1, motif 4)<br>Possible TFs: MXI1	*3.63076*	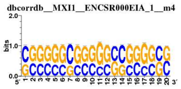	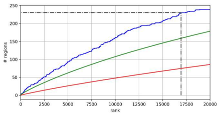	**link**	**link**	PWMs
155	☐ taipale_cyt_meth__SP8_NCCACGCCCMYN_eDBD_meth<br>Description: SP8 [Znf_C2H2, CpG-meth]<br>Possible TFs: SP8	*3.62997*	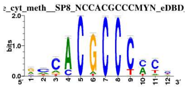	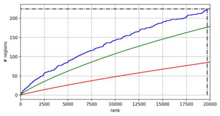	**link**	**link**	PWMs
156	☐ ENCFF001VZA<br>Description: DNase-seq on human GM06990	*3.62861*		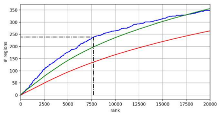	**link**	**link**	DHS & FAIRE
157	☐ transfac_pro__M05095<br>Description: P$ATERF11_01: ATERF11	*3.62712*	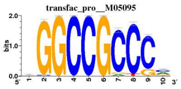	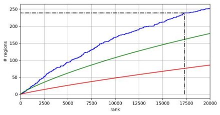	**link**	**link**	PWMs
158	☐ ENCFF001WFK<br>Description: DNase-seq on human GM06990	*3.62541*		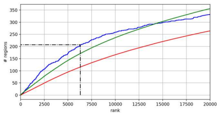	**link**	**link**	DHS & FAIRE

#	Feature	NES	Logo	Recovery Curve	Candidate targets	All regions in top 20000	Database
159	☐ cisbp__M6480<br>Description: SP1[gene ID: "ENSG00000185591" species: "Homo sapiens" TF status: "direct" TF family: "C2H2 ZF" DBDs: "zf-C2H2"]; SP5[gene ID: "ENSG00000204335" species: "Homo sapiens" TF status: "inferred" TF family: "C2H2 ZF" DBDs: "zf-C2H2"]; SP6[gene ID: "ENSG00000189120" species: "Homo sapiens" TF status: "inferred" TF family: "C2H2 ZF" DBDs: "zf-C2H2"]; SP7[gene ID: "ENSG00000170374" species: "Homo sapiens" TF status: "inferred" TF family: "C2H2 ZF" DBDs: "zf-C2H2"]; SP9[gene ID: "ENSG00000217236" species: "Homo sapiens" TF status: "inferred" TF family: "C2H2 ZF" DBDs: "zf-C2H2"]; Sp2[gene ID: "ENSMUSG00000018678" species: "Mus musculus" TF status: "inferred" TF family: "C2H2 ZF" DBDs: "zf-C2H2"]; Sp3[gene ID: "ENSMUSG00000027109" species: "Mus musculus" TF status: "inferred" TF family: "C2H2 ZF" DBDs: "zf-C2H2"]; Sp5[gene ID: "ENSMUSG00000075304" species: "Mus musculus" TF status: "inferred" TF family: "C2H2 ZF" DBDs: "zf-C2H2"]; Sp6[gene ID: "ENSMUSG00000038560" species: "Mus musculus" TF status: "inferred" TF family: "C2H2 ZF" DBDs: "zf-C2H2"]; Sp7[gene ID: "ENSMUSG00000060284" species: "Mus musculus" TF status: "inferred" TF family: "C2H2 ZF" DBDs: "zf-C2H2"]; Sp8[gene ID: "ENSMUSG00000048562" species: "Mus musculus" TF status: "inferred" TF family: "C2H2 ZF" DBDs: "zf-C2H2"]; Sp9[gene ID: "ENSMUSG00000068859" species: "Mus musculus" TF status: "inferred" TF family: "C2H2 ZF" DBDs: "zf-C2H2"]<br>Possible TFs: SP1	*3.62504*	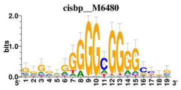	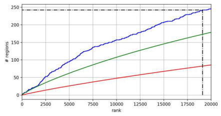	**link**	**link**	PWMs
160	☐ transfac_pro__M07520<br>Description: P$RAP26_03: RAP2-6	*3.62357*	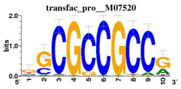	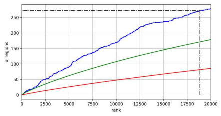	**link**	**link**	PWMs
161	☐ dbcorrdb__SREBF2__ENCSR000DYT_1__m7<br>Description: SREBF2 (ENCSR000DYT-1, motif 7)<br>Possible TFs: SREBF2	*3.61699*	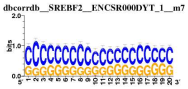	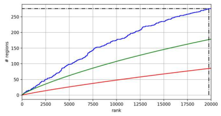	**link**	**link**	PWMs
162	☐ jaspar__MA1047.1<br>Description: TGA5	*3.61636*	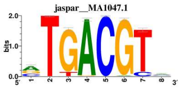	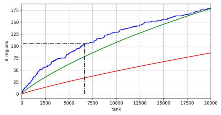	**link**	**link**	PWMs
163	☐ elemento__AGCCGCG<br>Description: Conserved regulatory element AGCCGCG between Hs and Mm	*3.61361*	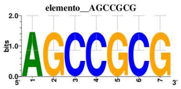	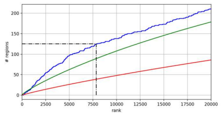	**link**	**link**	PWMs
164	☐ dbcorrdb__NRF1__ENCSR000EHZ_1__m1<br>Description: NRF1 (ENCSR000EHZ-1, motif 1)<br>Possible TFs: NRF1	*3.61134*	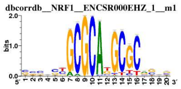	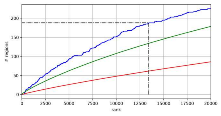	**link**	**link**	PWMs
165	☐ dbcorrdb__NRF1__ENCSR000EDJ_1__m1<br>Description: NRF1 (ENCSR000EDJ-1, motif 1)<br>Possible TFs: NRF1	*3.61063*	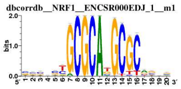	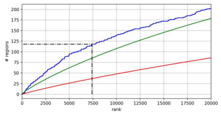	**link**	**link**	PWMs
166	☐ elemento__CGCGGCC<br>Description: Conserved regulatory element CGCGGCC between Hs and Mm	*3.60966*	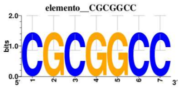	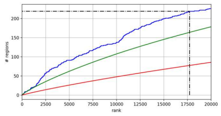	**link**	**link**	PWMs

#	Feature	NES	Logo	Recovery Curve	Candidate targets	All regions in top 20000	Database
167	☐ dbcorrdb__ZBTB33__ENCSR000BHR_1__m1<br>Description: ZBTB33 (ENCSR000BHR-1, motif 1)<br>Possible TFs: ZBTB33	3.60307	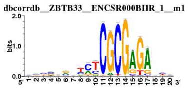	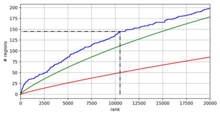	link	link	PWMs
168	☐ transfac_pro__M05360<br>Description: V$TIEG1_02: TIEG-1<br>Possible TFs: KLF10	3.60288	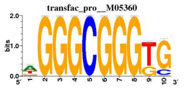	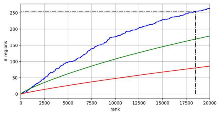	link	link	PWMs
169	☐ cisbp__M3088<br>Description: ATF2[gene ID: "ENSG00000115966" species: "Homo sapiens" TF status: "direct" TF family: "bZIP" DBDs: "bZIP_1"]; CREB5[gene ID: "ENSG00000146592" species: "Homo sapiens" TF status: "inferred" TF family: "bZIP" DBDs: "bZIP_1"]<br>Possible TFs: ATF2	3.59751	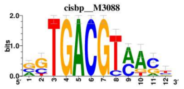	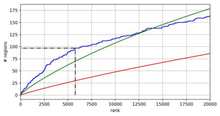	link	link	PWMs
170	☐ cisbp__M1553<br>Description: M1553	3.59666	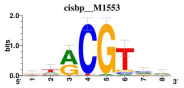	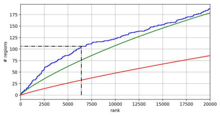	link	link	PWMs
171	☐ transfac_pro__M05332<br>Description: V$SP2_05: Sp2<br>Possible TFs: SP2	3.59087	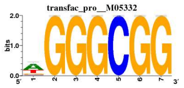	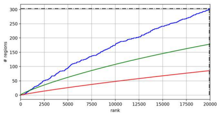	link	link	PWMs
172	☐ transfac_pro__M04971<br>Description: P$WRAF1_01: WRAF1	3.58943	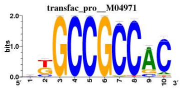	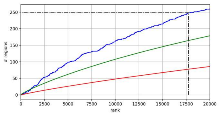	link	link	PWMs
173	☐ dbcorrdb__FOS__ENCSR000FAI_1__m1<br>Description: FOS (ENCSR000FAI-1, motif 1)<br>Possible TFs: FOS	3.58926	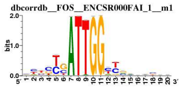	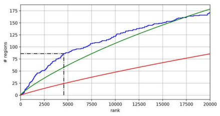	link	link	PWMs
174	☐ factorbook__NFY<br>Description: NFY<br>Possible TFs: NFYB, NFYA	3.58824	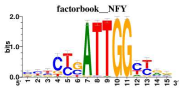	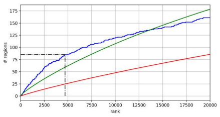	link	link	PWMs
175	☐ ENCFF001WNK<br>Description: DNase-seq on human Jurkat	3.58736		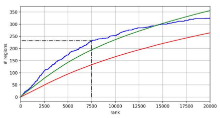	link	link	DHS & FAIRE
176	☐ transfac_pro__M00376<br>Description: P$TGA1A_Q2: TGA1a	3.58598	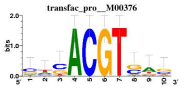	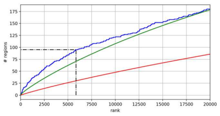	link	link	PWMs
177	☐ factorbook__CREB-ext<br>Description: CREB-ext<br>Possible TFs: ATF3	3.58520	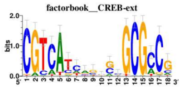	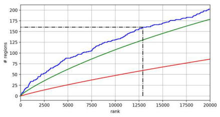	link	link	PWMs
178	☐ dbcorrdb__POLR2A__ENCSR000DLV_1__m1<br>Description: POLR2A (ENCSR000DLV-1, motif 1)<br>Possible TFs: POLR2A	3.58357	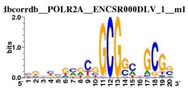	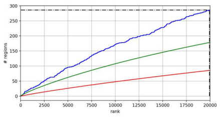	link	link	PWMs
179	☐ hdpi__TRIM21<br>Description: TRIM21<br>Possible TFs: TRIM21	3.58337	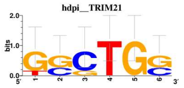	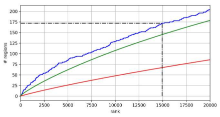	link	link	PWMs

#	Feature	NES	Logo	Recovery Curve	Candidate targets	All regions in top 20000	Database
180	☐ transfac_pro__M04991<br>Description: P$ERF4_01: ERF-4	3.57908	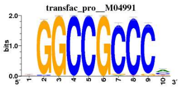	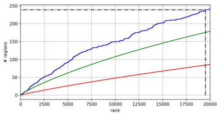	link	link	PWMs
181	☐ dbcorrdb__POLR2A__ENCSR000EUU_1__m1<br>Description: POLR2A (ENCSR000EUU-1, motif 1)<br>Possible TFs: POLR2A	3.57716	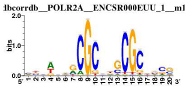	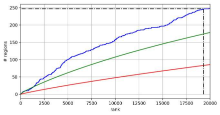	link	link	PWMs
182	☐ transfac_pro__M05361<br>Description: V$SP6_01: Sp6<br>Possible TFs: SP6	3.57618	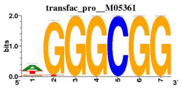	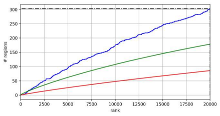	link	link	PWMs
183	☐ cisbp__M1781<br>Description: RSC3[gene ID: "YDR303C" species: "Saccharomyces cerevisiae" TF status: "direct" TF family: "Zinc cluster" DBDs: "Zn_clus"]	3.57538	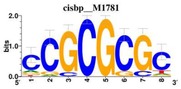	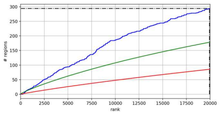	link	link	PWMs
184	☐ transfac_pro__M05665<br>Description: V$SP3_02: Sp3<br>Possible TFs: SP3	3.57177	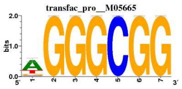	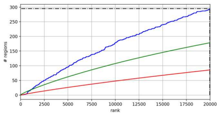	link	link	PWMs
185	☐ taipale_cyt_meth__SP9_NCCACGCCCMYN_eDBD_meth<br>Description: SP9 [Znf_C2H2, CpG-meth]<br>Possible TFs: SP9	3.57098	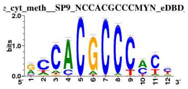	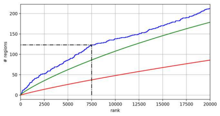	link	link	PWMs
186	☐ dbcorrdb__NRF1__ENCSR000EHH_1__m1<br>Description: NRF1 (ENCSR000EHH-1, motif 1)<br>Possible TFs: NRF1	3.56964	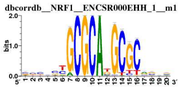	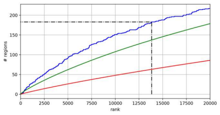	link	link	PWMs
187	☐ transfac_pro__M05455<br>Description: V$SP6_02: SP6<br>Possible TFs: SP6	3.56657	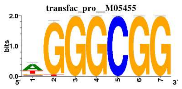	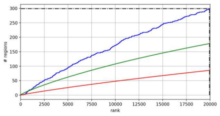	link	link	PWMs
188	☐ transfac_pro__M04998<br>Description: P$OPBP1_01: OPBP1	3.56614	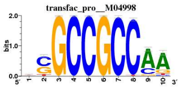	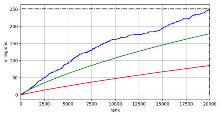	link	link	PWMs
189	☐ cisbp__M1551<br>Description: Gmeb2[gene ID: "ENSMUSG00000038705" species: "Mus musculus" TF status: "direct" TF family: "SAND" DBDs: "SAND"]<br>Possible TFs: GMEB2	3.56215	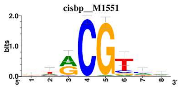	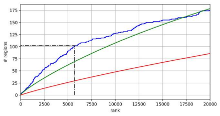	link	link	PWMs
190	☐ dbcorrdb__CHD2__ENCSR000ECP_1__m1<br>Description: CHD2 (ENCSR000ECP-1, motif 1)<br>Possible TFs: CHD2	3.55907	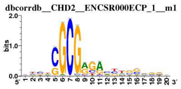	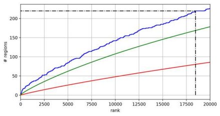	link	link	PWMs
191	☐ predrem__nrMotif1814<br>Description: 304_hTH1-DS7840.M463	3.55155	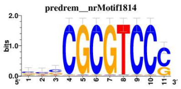	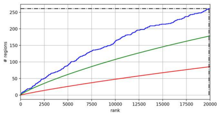	link	link	PWMs
192	☐ swissregulon__hs__CREB1.p2<br>Description: hs__CREB1.p2<br>Possible TFs: CREB1	3.55082	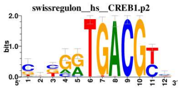	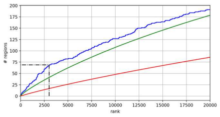	link	link	PWMs

#	Feature	NES	Logo	Recovery Curve	Candidate targets	All regions in top 20000	Database
193	taipale_cyt_meth__KLF13_NRCCACGCCCMYN_eDBD_meth<br>Description: KLF13 [Znf_C2H2, CpG-meth]<br>Possible TFs: KLF13	3.54834	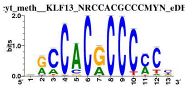	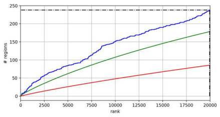	link	link	PWMs
194	taipale_cyt_meth__CREM_NRTGACGTCAYN_eDBD<br>Description: CREM [bZIP]<br>Possible TFs: CREM	3.54397	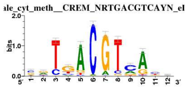	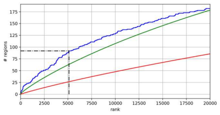	link	link	PWMs
195	dbcorrdb__SP1__ENCSR000BKO_1__m1<br>Description: SP1 (ENCSR000BKO-1, motif 1)<br>Possible TFs: SP1	3.54168	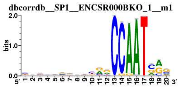	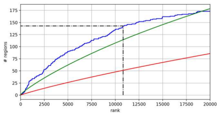	link	link	PWMs
196	transfac_public__M00287<br>Description: V$NFY_01: NF-Y<br>Possible TFs: NFYB	3.54123	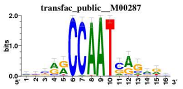	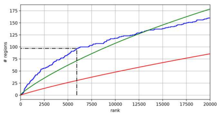	link	link	PWMs
197	factorbook__NRF1<br>Description: NRF1<br>Possible TFs: NRF1	3.53952	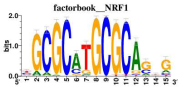	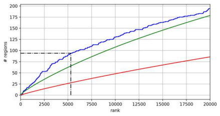	link	link	PWMs
198	jaspar__MA1052.1<br>Description: RAP2-6	3.53673	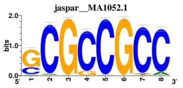	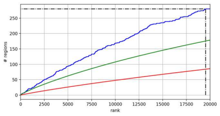	link	link	PWMs
199	dbcorrdb__IRF3__ENCSR000EEJ_1__m1<br>Description: IRF3 (ENCSR000EEJ-1, motif 1)<br>Possible TFs: IRF3	3.53312	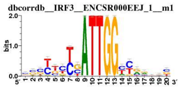	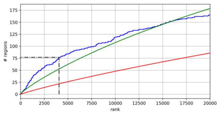	link	link	PWMs
200	ENCFF001WFJ<br>Description: DNase-seq on human GM06990	3.52485		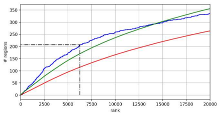	link	link	DHS & FAIRE
201	predrem__nrMotif2348<br>Description: 164_fMuscle_arm-DS18080.M589	3.52366	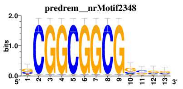	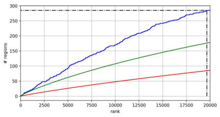	link	link	PWMs
202	ENCFF001SQG<br>Description: DNase-seq on human GM06990	3.52217		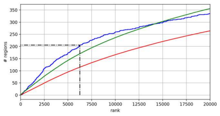	link	link	DHS & FAIRE
203	dbcorrdb__HDAC2__ENCSR000AQG_1__m4<br>Description: HDAC2 (ENCSR000AQG-1, motif 4)<br>Possible TFs: HDAC2	3.51625	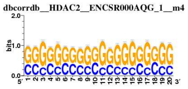	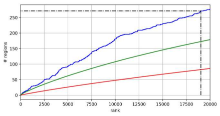	link	link	PWMs
204	transfac_pro__M00981<br>Description: V$CREBATF_Q6: CREB, ATF<br>Possible TFs: CREB1, CREM, ATF5, ATF4, ATF7, ATF6, ATF1, ATF3, ATF2	3.50682	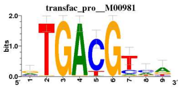	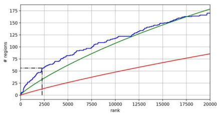	link	link	PWMs
205	transfac_pro__M05138<br>Description: P$ATERF8_01: ATERF8	3.50434	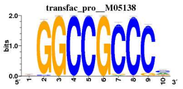	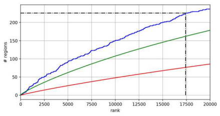	link	link	PWMs

#	Feature	NES	Logo	Recovery Curve	Candidate targets	All regions in top 20000	Database
206	☐ dbcorrdb__NRF1__ENCSR000DZO_1__m1<br>Description: NRF1 (ENCSR000DZO-1, motif 1)<br>Possible TFs: NRF1	*3.50430*	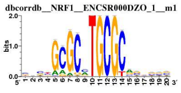	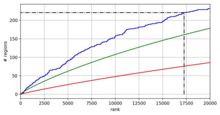	**link**	**link**	PWMs
207	☐ cisbp__M0613<br>Description: Mbd1[gene ID: "ENSMUSG00000024561" species: "Mus musculus" TF status: "direct" TF family: "CxxC" DBDs: "zf-CXXC"]<br>Possible TFs: MBD1	*3.50188*	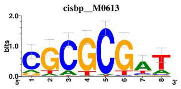	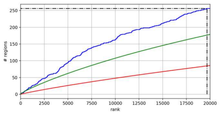	**link**	**link**	PWMs
208	☐ ENCFF001WCO<br>Description: DNase-seq on human Th1	*3.50125*		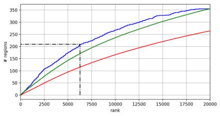	**link**	**link**	DHS & FAIRE
209	☐ transfac_pro__M05557<br>Description: V$BTEB3_01: BTEB3<br>Possible TFs: KLF13	*3.49987*	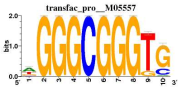	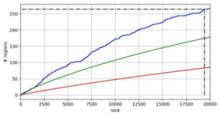	**link**	**link**	PWMs
210	☐ transfac_pro__M07302<br>Description: V$NFY_Q3: NF-Y<br>Possible TFs: NFYC, NFYB, NFYA	*3.49089*	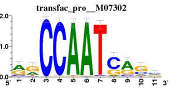	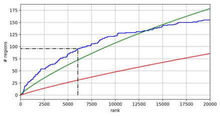	**link**	**link**	PWMs
211	☐ cisbp__M0297<br>Description: Creb1[gene ID: "ENSMUSG00000025958" species: "Mus musculus" TF status: "direct" TF family: "bZIP" DBDs: "bZIP_1"]; CrebB-17A[gene ID: "FBgn0014467" species: "Drosophila melanogaster" TF status: "inferred" TF family: "bZIP" DBDs: "bZIP_1"]<br>Possible TFs: CREB1	*3.48970*	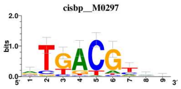	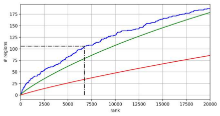	**link**	**link**	PWMs
212	☐ dbcorrdb__YY1__ENCSR000EXG_1__m2<br>Description: YY1 (ENCSR000EXG-1, motif 2)<br>Possible TFs: YY1	*3.48706*	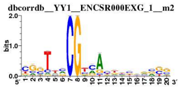	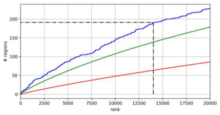	**link**	**link**	PWMs
213	☐ jaspar__MA0604.1<br>Description: Atf1<br>Possible TFs: ATF1	*3.48531*	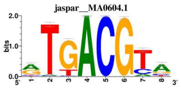	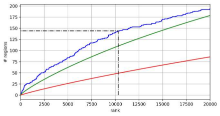	**link**	**link**	PWMs
214	☐ transfac_pro__M00944<br>Description: P$CPRF3_01: CPRF-3	*3.48500*	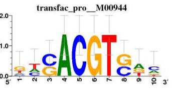	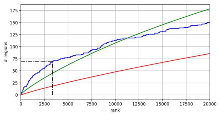	**link**	**link**	PWMs
215	☐ taipale_cyt_meth__KLF13_NRCCACGCCCMYN_FL_meth<br>Description: KLF13 [Znf_C2H2, CpG-meth]<br>Possible TFs: KLF13	*3.47908*	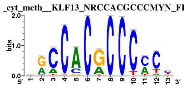	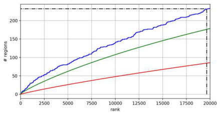	**link**	**link**	PWMs
216	☐ transfac_pro__M03544<br>Description: V$CREB1_Q6: CREB1<br>Possible TFs: CREB1	*3.47631*	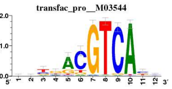	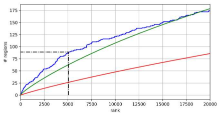	**link**	**link**	PWMs
217	☐ transfac_pro__M02106<br>Description: V$NFYA_Q5: NF-YA<br>Possible TFs: NFYA	*3.47246*	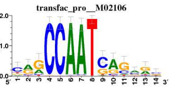	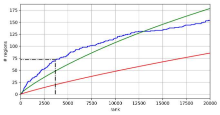	**link**	**link**	PWMs

#	Feature	NES	Logo	Recovery Curve	Candidate targets	All regions in top 20000	Database
218	☐ transfac_pro__M01928<br>Description: F$HAL9_02: HAL9	3.46914	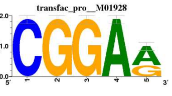	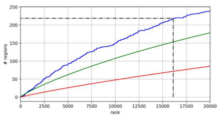	link	link	PWMs
219	☐ predrem__nrMotif1895<br>Description: 205_fMuscle_upper_back-DS19283.M616	3.46907	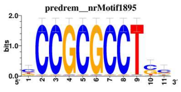	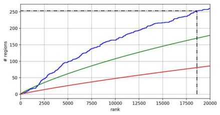	link	link	PWMs
220	☐ cisbp__M0295<br>Description: Atf1[gene ID: "ENSMUSG00000023027" species: "Mus musculus" TF status: "direct" TF family: "bZIP" DBDs: "bZIP_1"]; CrebB-17A[gene ID: "FBgn0014467" species: "Drosophila melanogaster" TF status: "inferred" TF family: "bZIP" DBDs: "bZIP_1"]<br>Possible TFs: ATF1	3.46879	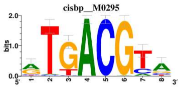	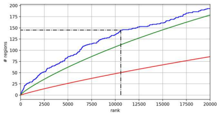	link	link	PWMs
221	☐ taipale_cyt_meth__KLF13_NRCCACGCCCMYN_FL_repr<br>Description: KLF13 [Znf_C2H2]<br>Possible TFs: KLF13	3.46877	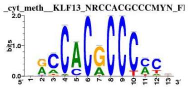	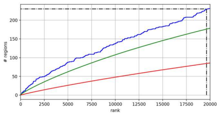	link	link	PWMs
222	☐ dbcorrdb__RFX5__ENCSR000DZW_1__m2<br>Description: RFX5 (ENCSR000DZW-1, motif 2)<br>Possible TFs: RFX5	3.46757	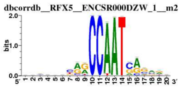	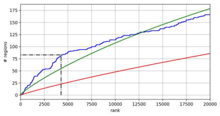	link	link	PWMs
223	☐ jaspar__MA0311.1<br>Description: HAL9	3.46680	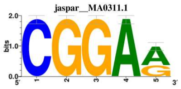	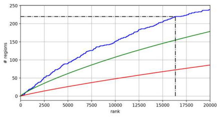	link	link	PWMs
224	☐ dbcorrdb__NRF1__ENCSR000ECC_1__m1<br>Description: NRF1 (ENCSR000ECC-1, motif 1)<br>Possible TFs: NRF1	3.46661	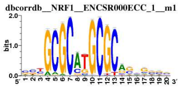	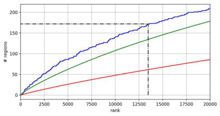	link	link	PWMs
225	☐ ENCFF001WTE<br>Description: DNase-seq on human Th1	3.46095		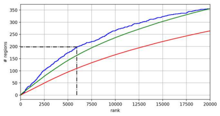	link	link	DHS & FAIRE
226	☐ scertf__badis.CST6<br>Description: badis.CST6	3.46000	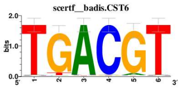	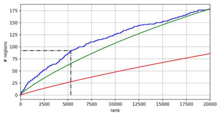	link	link	PWMs
227	☐ dbcorrdb__NRF1__ENCSR000EEH_1__m1<br>Description: NRF1 (ENCSR000EEH-1, motif 1)<br>Possible TFs: NRF1	3.45960	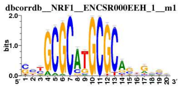	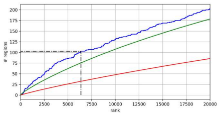	link	link	PWMs
228	☐ transfac_pro__M05000<br>Description: P$ERF112_01: ERF112	3.45904	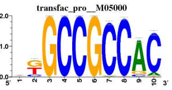	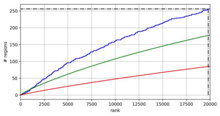	link	link	PWMs
229	☐ swissregulon__sacCer__RSC3<br>Description: sacCer__RSC3	3.44720	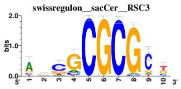	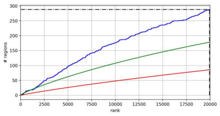	link	link	PWMs
230	☐ dbcorrdb__ATF3__ENCSR000BKE_1__m2<br>Description: ATF3 (ENCSR000BKE-1, motif 2)<br>Possible TFs: ATF3	3.44481	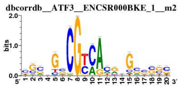	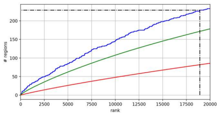	link	link	PWMs

#	Feature	NES	Logo	Recovery Curve	Candidate targets	All regions in top 20000	Database
231	☐ transfac_pro__M05301<br>Description: V$CPBP_01: CPBP<br>Possible TFs: KLF6	3.44368	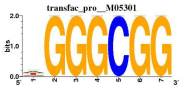	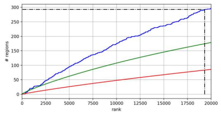	link	link	PWMs
232	☐ jaspar__MA0975.1<br>Description: CRF2	3.44323	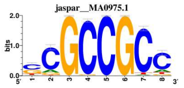	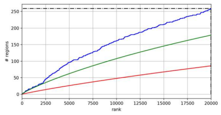	link	link	PWMs
233	☐ homer__AGCCAATCGG_NFY<br>Description: NFY(CCAAT)/Promoter/Homer	3.44058	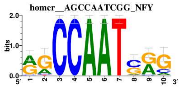	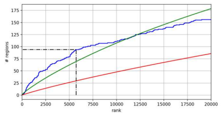	link	link	PWMs
234	☐ jaspar__MA0283.1<br>Description: CHA4	3.44030	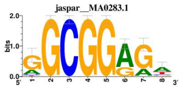	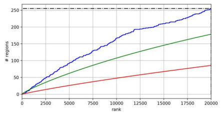	link	link	PWMs
235	☐ transfac_pro__M01916<br>Description: F$CHA4_02: Cha4p	3.44012	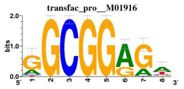	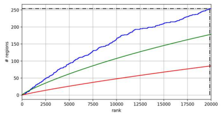	link	link	PWMs
236	☐ cisbp__M2088<br>Description: CHA4[gene ID: "YLR098C" species: "Saccharomyces cerevisiae" TF status: "direct" TF family: "Zinc cluster" DBDs: "Zn_clus"]	3.43551	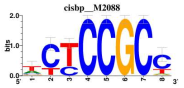	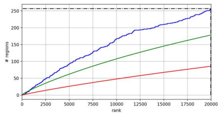	link	link	PWMs
237	☐ dbcorrdb__EZH2__ENCSR000ARK_1__m4<br>Description: EZH2 (ENCSR000ARK-1, motif 4)<br>Possible TFs: EZH2	3.43409	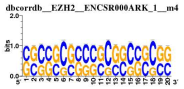	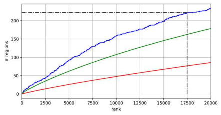	link	link	PWMs
238	☐ hocomoco__NRF1_HUMAN.H11MO.0.A<br>Description: NRF1_HUMAN<br>Possible TFs: NRF1	3.43391	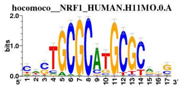	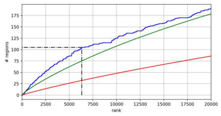	link	link	PWMs
239	☐ dbcorrdb__KDM5B__ENCSR000AQA_1__m2<br>Description: KDM5B (ENCSR000AQA-1, motif 2)<br>Possible TFs: KDM5B	3.43176	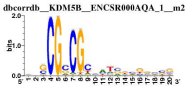	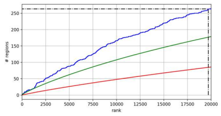	link	link	PWMs
240	☐ transfac_pro__M05391<br>Description: V$LKLF_01: LKLF<br>Possible TFs: KLF2	3.43067	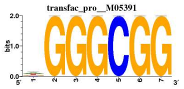	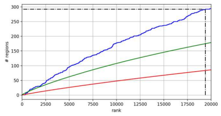	link	link	PWMs
241	☐ cisbp__M0673<br>Description: M0673	3.42995	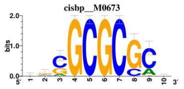	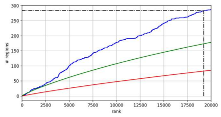	link	link	PWMs
242	☐ swissregulon__hs__NRF1.p2<br>Description: hs__NRF1.p2<br>Possible TFs: NRF1	3.42801	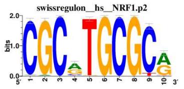	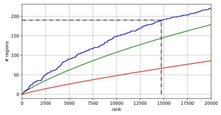	link	link	PWMs
243	☐ transfac_pro__M05444<br>Description: V$CPBP_02: CPBP<br>Possible TFs: KLF6	3.42722	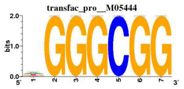	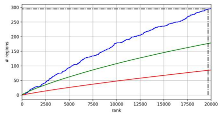	link	link	PWMs

#	Feature	NES	Logo	Recovery Curve	Candidate targets	All regions in top 20000	Database
244	cisbp__M1544<br>Description: M1544	3.42128	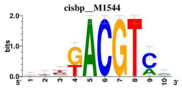	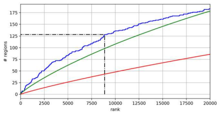	link	link	PWMs
245	dbcorrdb__POLR2A__ENCSR000DMK_1__m1<br>Description: POLR2A (ENCSR000DMK-1, motif 1)<br>Possible TFs: POLR2A	3.41589	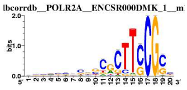	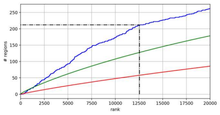	link	link	PWMs
246	transfac_pro__M05029<br>Description: P$CBF2_03: CBF2	3.41543	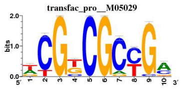	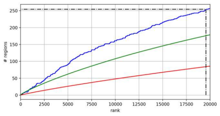	link	link	PWMs
247	hocomoco__NFYB_HUMAN.H11MO.0.A<br>Description: NFYB_HUMAN<br>Possible TFs: NFYB	3.41341	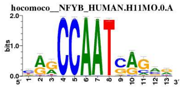	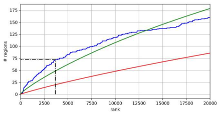	link	link	PWMs
248	swissregulon__sacCer__CST6<br>Description: sacCer__CST6	3.41138	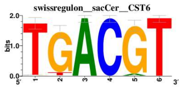	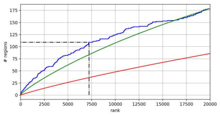	link	link	PWMs
249	dbcorrdb__POLR2A__ENCSR000BKI_1__m1<br>Description: POLR2A (ENCSR000BKI-1, motif 1)<br>Possible TFs: POLR2A	3.41096	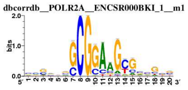	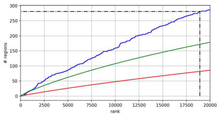	link	link	PWMs
250	transfac_pro__M05499<br>Description: V$LKLF_02: LKLF<br>Possible TFs: KLF2	3.40630	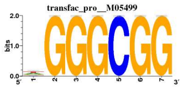	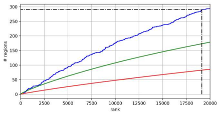	link	link	PWMs
251	hocomoco__NRF1_MOUSE.H11MO.0.A<br>Description: NRF1_MOUSE<br>Possible TFs: NRF1	3.40567	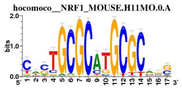	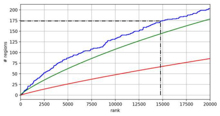	link	link	PWMs
252	dbcorrdb__NFYB__ENCSR000DNM_1__m1<br>Description: NFYB (ENCSR000DNM-1, motif 1)<br>Possible TFs: NFYB	3.40553	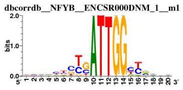	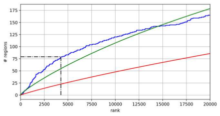	link	link	PWMs
253	factorbook__UA1<br>Description: UA1<br>Possible TFs: ZBTB33	3.40030	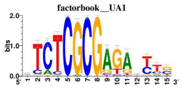	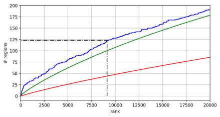	link	link	PWMs
254	cisbp__M0275<br>Description: M0275	3.39954	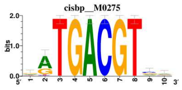	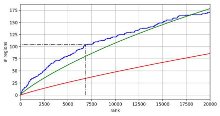	link	link	PWMs
255	transfac_pro__M05023<br>Description: P$ERF110_02: ERF110	3.39924	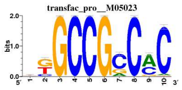	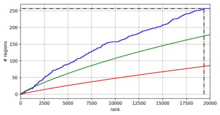	link	link	PWMs
256	elemento__CGCGCGC<br>Description: Conserved regulatory element CGCGCGC between Hs and Mm	3.39734	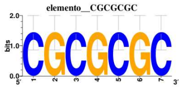	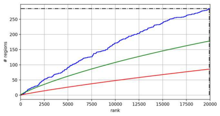	link	link	PWMs

#	Feature	NES	Logo	Recovery Curve	Candidate targets	All regions in top 20000	Database
257	☐ hocomoco__HAND1_HUMAN.H11MO.1.D<br>Description: HAND1_HUMAN<br>Possible TFs: HAND1	3.39442	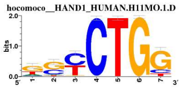	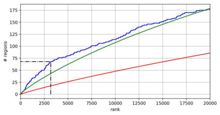	link	link	PWMs
258	☐ jaspar__MA0527.1<br>Description: ZBTB33<br>Possible TFs: ZBTB33	3.39386	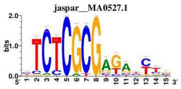	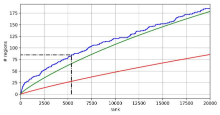	link	link	PWMs
259	☐ cisbp__M6371<br>Description: NFYA[gene ID: "ENSG00000001167" species: "Homo sapiens" TF status: "direct" TF family: "CBF/NF-Y" DBDs: "CBFB_NFYA"]; Nf-YA[gene ID: "FBgn0035993" species: "Drosophila melanogaster" TF status: "inferred" TF family: "CBF/NF-Y" DBDs: "CBFB_NFYA"]; Nfya[gene ID: "ENSMUSG00000023994" species: "Mus musculus" TF status: "inferred" TF family: "CBF/NF-Y" DBDs: "CBFB_NFYA"]<br>Possible TFs: NFYA	3.39372	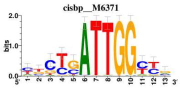	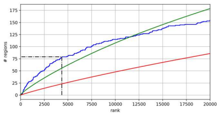	link	link	PWMs
260	☐ ENCFF001SPD<br>Description: DNase-seq on human ishikawa treated with 4-hydroxy-tamoxifen at 100nM for 30 minutes	3.39358		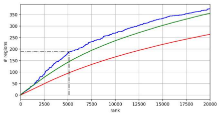	link	link	DHS & FAIRE
261	☐ transfac_public__M00017<br>Description: V$ATF_01: ATF	3.39157	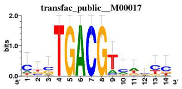	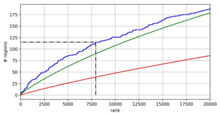	link	link	PWMs
262	☐ ENCFF001SOP<br>Description: Duke human GM18507 DNase-seq	3.38529		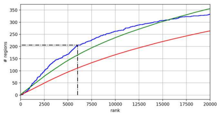	link	link	DHS & FAIRE
263	☐ cisbp__M3083<br>Description: CREB1[gene ID: "ENSG00000118260" species: "Homo sapiens" TF status: "direct" TF family: "bZIP" DBDs: "bZIP_1"]; CrebB-17A[gene ID: "FBgn0014467" species: "Drosophila melanogaster" TF status: "inferred" TF family: "bZIP" DBDs: "bZIP_1"]<br>Possible TFs: CREB1	3.38217	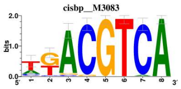	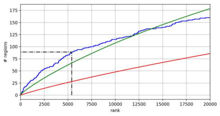	link	link	PWMs
264	☐ jaspar__MA0320.1<br>Description: IME1	3.38131	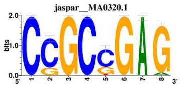	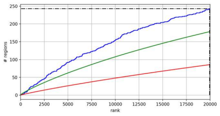	link	link	PWMs
265	☐ flyfactorsurvey__CG3065_F1-3_SOLEXA_2.5_FBgn0034946<br>Description: CG3065_F1-3_SOLEXA_2.5_FBgn0034946<br>Possible TFs: KLF18, KLF17	3.37966	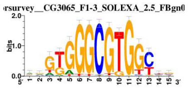	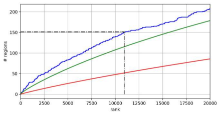	link	link	PWMs
266	☐ elemento__CGCGGGA<br>Description: Conserved regulatory element CGCGGGA between Hs and Mm	3.37930	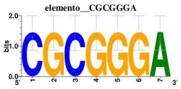	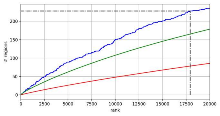	link	link	PWMs
267	☐ dbcorrdb__POLR2A__ENCSR000EAR_1__m1<br>Description: POLR2A (ENCSR000EAR-1, motif 1)<br>Possible TFs: POLR2A	3.37808	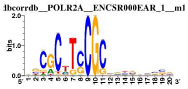	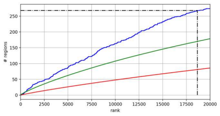	link	link	PWMs
268	☐ cisbp__M0029<br>Description: M0029	3.37527	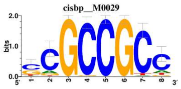	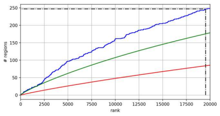	link	link	PWMs

#	Feature	NES	Logo	Recovery Curve	Candidate targets	All regions in top 20000	Database
269	☐ dbcorrdb__CHD2__ENCSR000EBT_1__m2<br>Description: CHD2 (ENCSR000EBT-1, motif 2)<br>Possible TFs: CHD2	*3.37365*	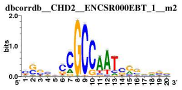	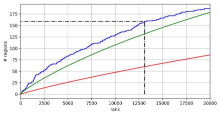	link	link	PWMs
270	☐ transfac_pro__M09580<br>Description: P$TGA6_03: TGA6	*3.37081*	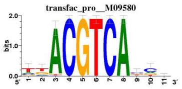	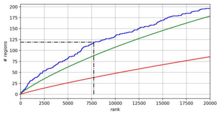	link	link	PWMs
271	☐ transfac_pro__M01186<br>Description: P$STF1_01: STF1	*3.36794*	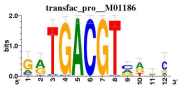	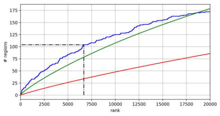	link	link	PWMs
272	☐ dbcorrdb__RBBP5__ENCSR000AQC_1__m5<br>Description: RBBP5 (ENCSR000AQC-1, motif 5)<br>Possible TFs: RBBP5	*3.36727*	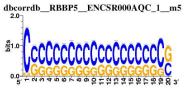	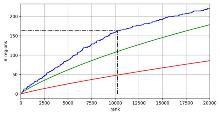	link	link	PWMs
273	☐ dbcorrdb__ESRRA__ENCSR000DYQ_1__m5<br>Description: ESRRA (ENCSR000DYQ-1, motif 5)<br>Possible TFs: ESRRA	*3.36715*	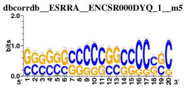	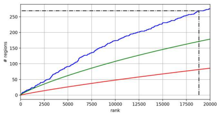	link	link	PWMs
274	☐ transfac_pro__M09066<br>Description: P$ERF105_03: ERF105	*3.36713*	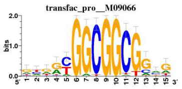	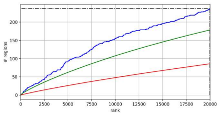	link	link	PWMs
275	☐ transfac_pro__M05386<br>Description: V$KLF17_01: KLF17<br>Possible TFs: KLF17	*3.36639*	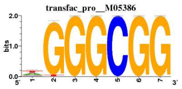	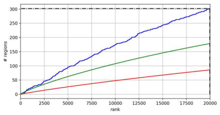	link	link	PWMs
276	☐ hdpi__NAP1L1<br>Description: NAP1L1<br>Possible TFs: NAP1L1	*3.36401*	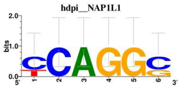	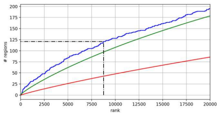	link	link	PWMs
277	☐ transfac_pro__M01742<br>Description: V$ZFP206_01: Zfp206<br>Possible TFs: ZSCAN10	*3.36065*	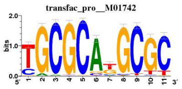	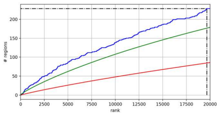	link	link	PWMs
278	☐ ENCFF001SPK<br>Description: DNase-seq on human PanIsletD	*3.35994*		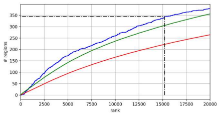	link	link	DHS & FAIRE
279	☐ ENCFF001SQD<br>Description: DNase-seq on human CD20+ (RO01778)	*3.35976*		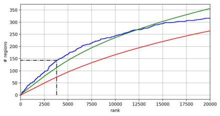	link	link	DHS & FAIRE
280	☐ cisbp__M2125<br>Description: IME1[gene ID: "YJR094C" species: "Saccharomyces cerevisiae" TF status: "direct" TF family: "Unknown" DBDs: "UNKNOWN"]	*3.35827*	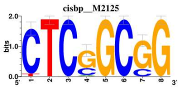	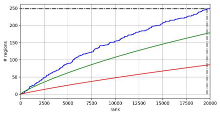	link	link	PWMs
281	☐ ENCFF001WEQ<br>Description: DNase-seq on human CD20+ (RO01778)	*3.35678*		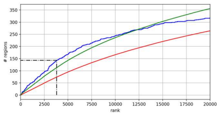	link	link	DHS & FAIRE

#	Feature	NES	Logo	Recovery Curve	Candidate targets	All regions in top 20000	Database
282	☐ dbcorrdb__NFYA__ENCSR000DNN_1__m1<br>Description: NFYA (ENCSR000DNN-1, motif 1)<br>Possible TFs: NFYA	3.35656	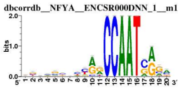	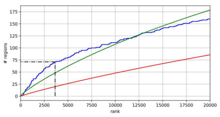	link	link	PWMs
283	☐ transfac_pro__M05105<br>Description: P$AT1G68550_01: At1g68550	3.35616	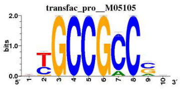	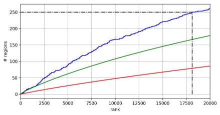	link	link	PWMs
284	☐ transfac_pro__M05134<br>Description: P$AT3G61630_01: At3g61630	3.35515	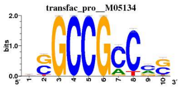	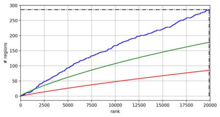	link	link	PWMs
285	☐ dbcorrdb__SP1__ENCSR000BIR_1__m1<br>Description: SP1 (ENCSR000BIR-1, motif 1)<br>Possible TFs: SP1	3.35360	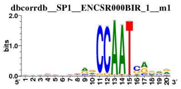	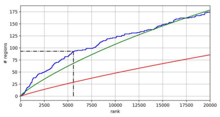	link	link	PWMs
286	☐ hocomoco__ATF1_HUMAN.H11MO.0.B<br>Description: ATF1_HUMAN<br>Possible TFs: ATF1	3.35256	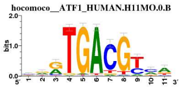	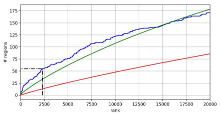	link	link	PWMs
287	☐ yetfasco__YDR303C_580<br>Description: YDR303C_580	3.35020	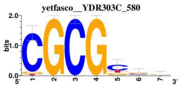	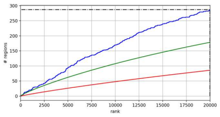	link	link	PWMs
288	☐ transfac_pro__M05407<br>Description: V$KLF17_02: Klf17<br>Possible TFs: KLF17	3.34528	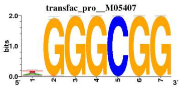	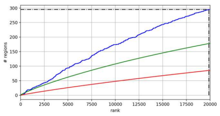	link	link	PWMs
289	☐ transfac_pro__M01934<br>Description: F$RSC3_02: Rsc3p	3.34379	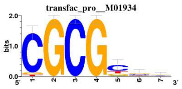	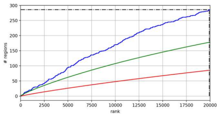	link	link	PWMs
290	☐ cisbp__M4783<br>Description: Bteb2[gene ID: "FBgn0025679" species: "Drosophila melanogaster" TF status: "direct" TF family: "C2H2 ZF" DBDs: "zf-C2H2"]<br>Possible TFs: KLF15	3.34192	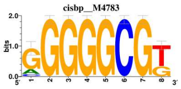	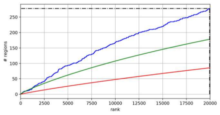	link	link	PWMs
291	☐ jaspar__MA0374.1<br>Description: RSC3	3.34074	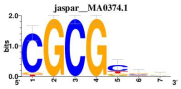	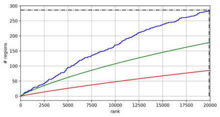	link	link	PWMs
292	☐ jaspar__MA0980.1<br>Description: RAP2-10	3.33918	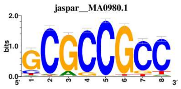	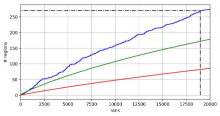	link	link	PWMs
293	☐ swissregulon__hs__NFY_A_B_C_.p2<br>Description: hs__NFY{A,B,C}.p2<br>Possible TFs: NFYC, NFYB, NFYA	3.33350	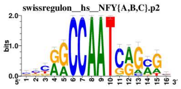	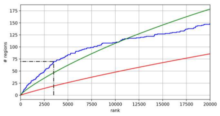	link	link	PWMs
294	☐ cisbp__M1789<br>Description: RSC30[gene ID: "YHR056C" species: "Saccharomyces cerevisiae" TF status: "direct" TF family: "Zinc cluster" DBDs: "Zn_clus"]	3.33308	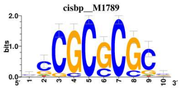	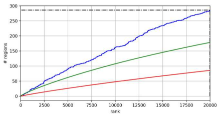	link	link	PWMs

#	Feature	NES	Logo	Recovery Curve	Candidate targets	All regions in top 20000	Database
295	☐ dbcorrdb__SIN3A__ENCSR000BGL_1__m1<br>Description: SIN3A (ENCSR000BGL-1, motif 1)<br>Possible TFs: SIN3A	*3.33306*	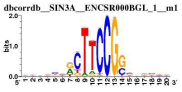	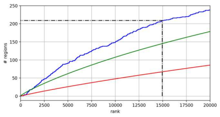	**link**	**link**	PWMs
296	☐ cisbp__M0392<br>Description: SP5[gene ID: "ENSG00000204335" species: "Homo sapiens" TF status: "inferred" TF family: "C2H2 ZF" DBDs: "zf-C2H2"]; SP6[gene ID: "ENSG00000189120" species: "Homo sapiens" TF status: "inferred" TF family: "C2H2 ZF" DBDs: "zf-C2H2"]; SP7[gene ID: "ENSG00000170374" species: "Homo sapiens" TF status: "inferred" TF family: "C2H2 ZF" DBDs: "zf-C2H2"]; SP9[gene ID: "ENSG00000217236" species: "Homo sapiens" TF status: "inferred" TF family: "C2H2 ZF" DBDs: "zf-C2H2"]; Sp1[gene ID: "ENSMUSG00000001280" species: "Mus musculus" TF status: "direct" TF family: "C2H2 ZF" DBDs: "zf-C2H2"]; Sp2[gene ID: "ENSMUSG00000018678" species: "Mus musculus" TF status: "inferred" TF family: "C2H2 ZF" DBDs: "zf-C2H2"]; Sp3[gene ID: "ENSMUSG00000027109" species: "Mus musculus" TF status: "inferred" TF family: "C2H2 ZF" DBDs: "zf-C2H2"]; Sp5[gene ID: "ENSMUSG00000075304" species: "Mus musculus" TF status: "inferred" TF family: "C2H2 ZF" DBDs: "zf-C2H2"]; Sp6[gene ID: "ENSMUSG00000038560" species: "Mus musculus" TF status: "inferred" TF family: "C2H2 ZF" DBDs: "zf-C2H2"]; Sp7[gene ID: "ENSMUSG00000060284" species: "Mus musculus" TF status: "inferred" TF family: "C2H2 ZF" DBDs: "zf-C2H2"]; Sp8[gene ID: "ENSMUSG00000048562" species: "Mus musculus" TF status: "inferred" TF family: "C2H2 ZF" DBDs: "zf-C2H2"]; Sp9[gene ID: "ENSMUSG00000068859" species: "Mus musculus" TF status: "inferred" TF family: "C2H2 ZF" DBDs: "zf-C2H2"]<br>Possible TFs: SP1	*3.33305*	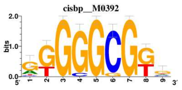	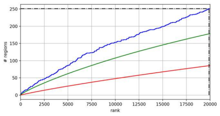	**link**	**link**	PWMs
297	☐ transfac_pro__M04969<br>Description: P$ABI4_03: ABI4	*3.33049*	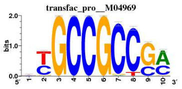	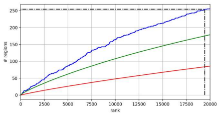	**link**	**link**	PWMs
298	☐ transfac_pro__M05096<br>Description: P$ATERF9_01: ATERF9	*3.32843*	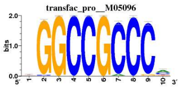	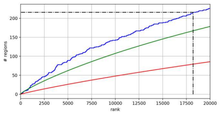	**link**	**link**	PWMs
299	☐ dbcorrdb__HCFC1__ENCSR000ECH_1__m3<br>Description: HCFC1 (ENCSR000ECH-1, motif 3)<br>Possible TFs: HCFC1	*3.32801*	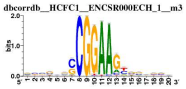	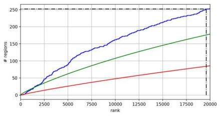	**link**	**link**	PWMs
300	☐ transfac_pro__M04760<br>Description: V$NR3C1_06: GR<br>Possible TFs: NR3C1	*3.32670*	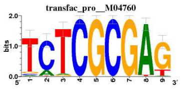	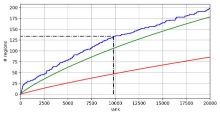	**link**	**link**	PWMs
301	☐ cisbp__M6372<br>Description: NFYB[gene ID: "ENSG00000120837" species: "Homo sapiens" TF status: "direct" TF family: "Unknown" DBDs: "UNKNOWN"]<br>Possible TFs: NFYB	*3.32637*	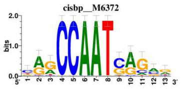	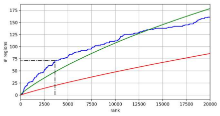	**link**	**link**	PWMs
302	☐ cisbp__M1545<br>Description: GMEB1[gene ID: "ENSG00000162419" species: "Homo sapiens" TF status: "direct" TF family: "SAND" DBDs: "SAND"]<br>Possible TFs: GMEB1	*3.32505*	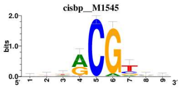	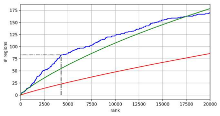	**link**	**link**	PWMs

#	Feature	NES	Logo	Recovery Curve	Candidate targets	All regions in top 20000	Database
303	☐ hocomoco__SP3_HUMAN.H11MO.0.B<br>Description: SP3_HUMAN<br>Possible TFs: SP3	*3.32052*	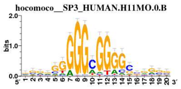	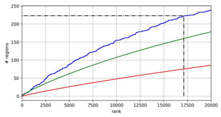	**link**	**link**	PWMs
304	☐ dbcorrdb__CHD2__ENCSR000DZR_1__m1<br>Description: CHD2 (ENCSR000DZR-1, motif 1)<br>Possible TFs: CHD2	*3.31995*	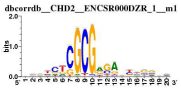	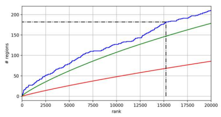	**link**	**link**	PWMs
305	☐ dbcorrdb__NELFE__ENCSR000DOF_1__m6<br>Description: NELFE (ENCSR000DOF-1, motif 6)<br>Possible TFs: NELFE	*3.31816*	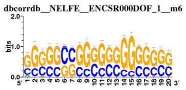	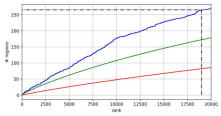	**link**	**link**	PWMs
306	☐ ENCFF001SPC<br>Description: DNase-seq on human ishikawa treated with estradiol at 10nM for 30 minutes	*3.31478*		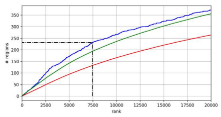	**link**	**link**	DHS & FAIRE
307	☐ cisbp__M0025<br>Description: M0025	*3.31474*	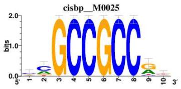	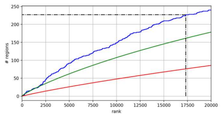	**link**	**link**	PWMs
308	☐ hdpi__AKR1A1<br>Description: AKR1A1<br>Possible TFs: AKR1A1	*3.31365*	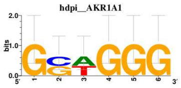	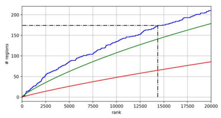	**link**	**link**	PWMs
309	☐ taipale_cyt_meth__KLF13_NRCCACGCCCMYN_eDBD<br>Description: KLF13 [Znf_C2H2]<br>Possible TFs: KLF13	*3.31331*	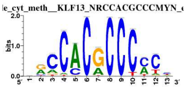	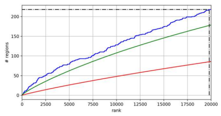	**link**	**link**	PWMs
310	☐ dbcorrdb__GABPA__ENCSR000BGC_1__m1<br>Description: GABPA (ENCSR000BGC-1, motif 1)<br>Possible TFs: GABPA	*3.31277*	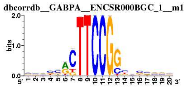	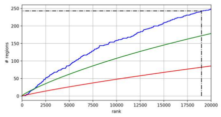	**link**	**link**	PWMs
311	☐ dbcorrdb__SMARCA4__ENCSR000EZC_1__m10<br>Description: SMARCA4 (ENCSR000EZC-1, motif 10)<br>Possible TFs: SMARCA4	*3.31067*	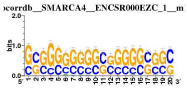	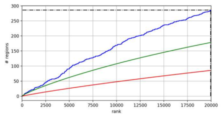	**link**	**link**	PWMs
312	☐ transfac_pro__M02773<br>Description: V$KLF7_03: Klf7<br>Possible TFs: KLF7	*3.31061*	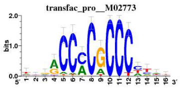	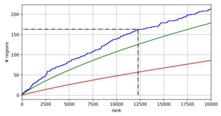	**link**	**link**	PWMs
313	☐ transfac_pro__M05145<br>Description: P$DREB2D_01: DREB 2D	*3.31028*	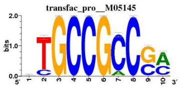	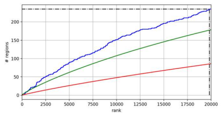	**link**	**link**	PWMs
314	☐ ENCFF001SSJ<br>Description: DNase-seq on human Th2	*3.30585*		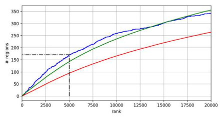	**link**	**link**	DHS & FAIRE
315	☐ transfac_pro__M07052<br>Description: V$NRF1_Q5: NRF-1<br>Possible TFs: NRF1	*3.30536*	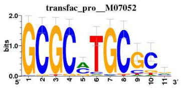	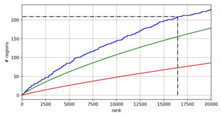	**link**	**link**	PWMs

#	Feature	NES	Logo	Recovery Curve	Candidate targets	All regions in top 20000	Database
316	☐ dbcorrdb__POLR2A__ENCSR000BQB_1__m1<br>Description: POLR2A (ENCSR000BQB-1, motif 1)<br>Possible TFs: POLR2A	*3.30343*	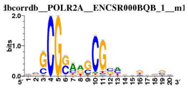	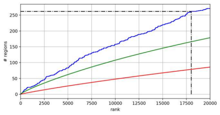	link	link	PWMs
317	☐ ENCFF001WTO<br>Description: DNase-seq on human Th2	*3.30006*		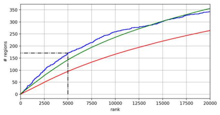	link	link	DHS & FAIRE
318	☐ scertf__badis.RSC3<br>Description: badis.RSC3	*3.29473*	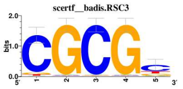	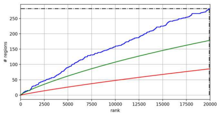	link	link	PWMs
319	☐ transfac_pro__M00370<br>Description: P$CPRF3_Q2: CPRF-3	*3.29381*	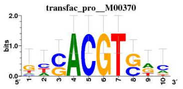	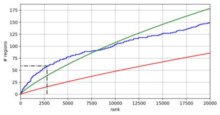	link	link	PWMs
320	☐ dbcorrdb__ZBTB33__ENCSR000BHC_1__m1<br>Description: ZBTB33 (ENCSR000BHC-1, motif 1)<br>Possible TFs: ZBTB33	*3.29200*	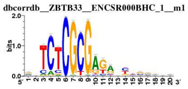	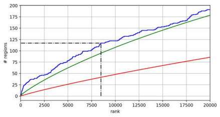	link	link	PWMs
321	☐ transfac_pro__M04694<br>Description: V$NRF1_03: NRF-1<br>Possible TFs: NRF1	*3.28469*	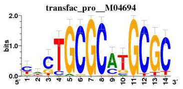	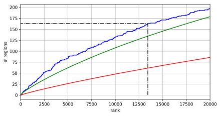	link	link	PWMs
322	☐ predrem__nrMotif1056<br>Description: 151_fLung_R-DS16566.M380	*3.28369*	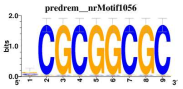	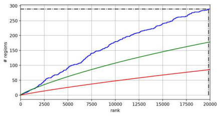	link	link	PWMs
323	☐ cisbp__M6197<br>Description: E4F1[gene ID: "ENSG00000167967" species: "Homo sapiens" TF status: "direct" TF family: "C2H2 ZF" DBDs: "zf-C2H2"]<br>Possible TFs: E4F1	*3.27999*	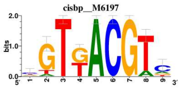	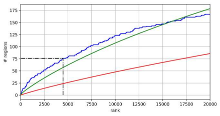	link	link	PWMs
324	☐ transfac_pro__M05069<br>Description: P$ATERF12_01: ATERF12	*3.27738*	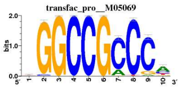	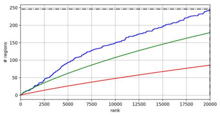	link	link	PWMs
325	☐ predrem__nrMotif2242<br>Description: 234_fStomach-DS17172.M267	*3.27605*	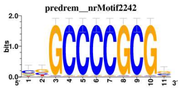	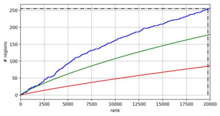	link	link	PWMs
326	☐ elemento__CCGCCTC<br>Description: Conserved regulatory element CCGCCTC between Hs and Mm	*3.27531*	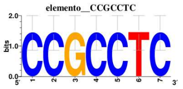	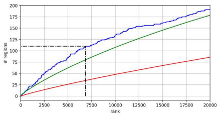	link	link	PWMs
327	☐ E093-DNase.hotspot.all.peaks-narrowpeak<br>Description: DNase in Fetal Thymus (E093, narrowpeak, hotspot)	*3.27211*		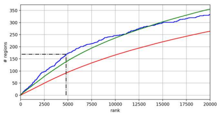	link	link	DHS & FAIRE
328	☐ jaspar__MA0096.1<br>Description: bZIP910	*3.27130*	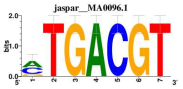	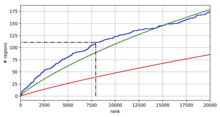	link	link	PWMs

#	Feature	NES	Logo	Recovery Curve	Candidate targets	All regions in top 20000	Database
329	☐ dbcorrdb__POLR2A__ENCSR000DLQ_1__m2<br>Description: POLR2A (ENCSR000DLQ-1, motif 2)<br>Possible TFs: POLR2A	3.27055	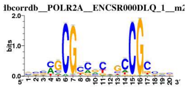	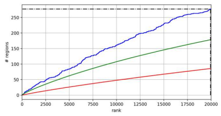	link	link	PWMs
330	☐ ENCFF001WIV<br>Description: DNase-seq on human HepG2	3.27023		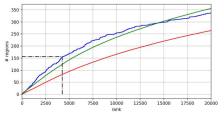	link	link	DHS & FAIRE
331	☐ transfac_pro__M05094<br>Description: P$AT1G24590_01: At1g24590	3.26453	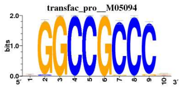	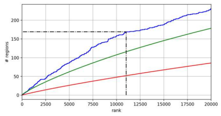	link	link	PWMs
332	☐ elemento__CGCGTGAC<br>Description: Conserved regulatory element CGCGTGAC between Hs and Mm	3.26350	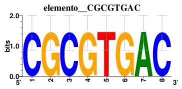	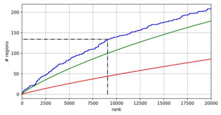	link	link	PWMs
333	☐ taipale_cyt_meth__XBP1_NRTGACGTCAYN_FL<br>Description: XBP1 [bZIP]<br>Possible TFs: XBP1	3.26280	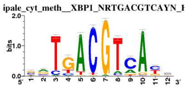	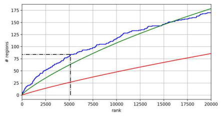	link	link	PWMs
334	☐ transfac_pro__M05893<br>Description: V$ZNF676_04: ZNF676<br>Possible TFs: ZNF676	3.26249	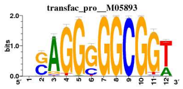	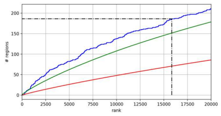	link	link	PWMs
335	☐ cisbp__M1920<br>Description: M1920	3.25963	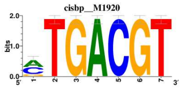	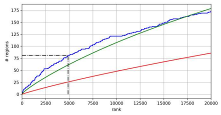	link	link	PWMs
336	☐ ENCFF001SPR<br>Description: DNase-seq on human adult CD4 Th0	3.25948		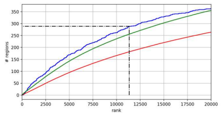	link	link	DHS & FAIRE
337	☐ transfac_pro__M07449<br>Description: F$FL_01: FL	3.25822	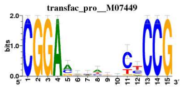	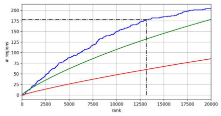	link	link	PWMs
338	☐ E092-DNase.hotspot.all.peaks-narrowpeak<br>Description: DNase in Fetal Stomach (E092, narrowpeak, hotspot)	3.25615		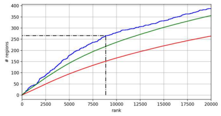	link	link	DHS & FAIRE
339	☐ dbcorrdb__POLR2A__ENCSR000DLJ_1__m1<br>Description: POLR2A (ENCSR000DLJ-1, motif 1)<br>Possible TFs: POLR2A	3.25592	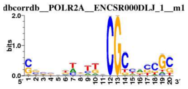	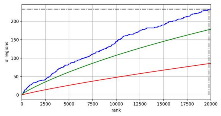	link	link	PWMs
340	☐ cisbp__M1383<br>Description: M1383	3.25551	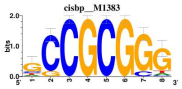	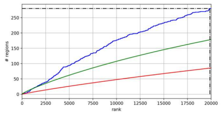	link	link	PWMs
341	☐ transfac_pro__M09033<br>Description: P$AT4G18450_01: AT4G18450	3.25543	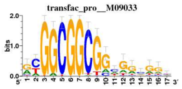	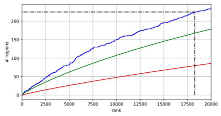	link	link	PWMs

#	Feature	NES	Logo	Recovery Curve	Candidate targets	All regions in top 20000	Database
342	☐ elemento__CGCGAGA<br>Description: Conserved regulatory element CGCGAGA between Hs and Mm	*3.25473*	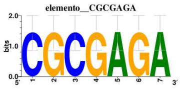	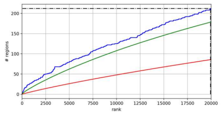	link	link	PWMs
343	☐ transfac_pro__M05035<br>Description: P$TSRF1_01: TSRF1	*3.25336*	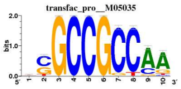	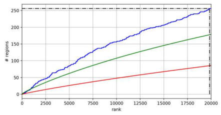	link	link	PWMs
344	☐ jaspar__MA1051.1<br>Description: RAP2-3	*3.24970*	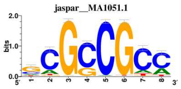	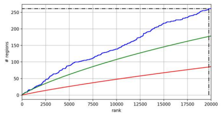	link	link	PWMs
345	☐ jaspar__MA1069.1<br>Description: TGA6	*3.24596*	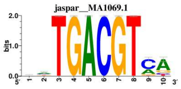	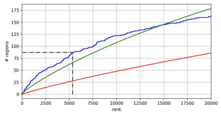	link	link	PWMs
346	☐ cisbp__M1280<br>Description: M1280	*3.24502*	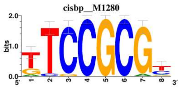	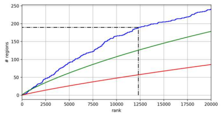	link	link	PWMs
347	☐ dbcorrdb__ATF3__ENCSR000BKE_1__m1<br>Description: ATF3 (ENCSR000BKE-1, motif 1)<br>Possible TFs: ATF3	*3.24428*	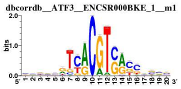	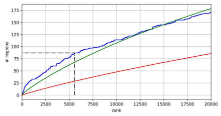	link	link	PWMs
348	☐ transfac_pro__M04975<br>Description: P$ERF3_01: ERF3	*3.24346*	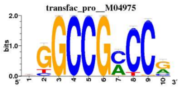	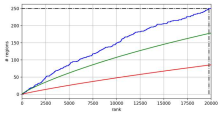	link	link	PWMs
349	☐ transfac_pro__M01783<br>Description: V$SP2_01: SP2<br>Possible TFs: SP2	*3.24092*	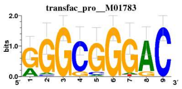	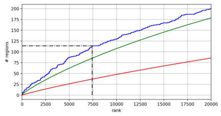	link	link	PWMs

#	Feature	NES	Logo	Recovery Curve	Candidate targets	All regions in top 20000	Database
350	☐ cisbp__M6482<br>Description: SP3[gene ID: "ENSG00000172845" species: "Homo sapiens" TF status: "direct" TF family: "C2H2 ZF" DBDs: "zf-C2H2"]; SP5[gene ID: "ENSG00000204335" species: "Homo sapiens" TF status: "inferred" TF family: "C2H2 ZF" DBDs: "zf-C2H2"]; SP6[gene ID: "ENSG00000189120" species: "Homo sapiens" TF status: "inferred" TF family: "C2H2 ZF" DBDs: "zf-C2H2"]; SP7[gene ID: "ENSG00000170374" species: "Homo sapiens" TF status: "inferred" TF family: "C2H2 ZF" DBDs: "zf-C2H2"]; SP9[gene ID: "ENSG00000217236" species: "Homo sapiens" TF status: "inferred" TF family: "C2H2 ZF" DBDs: "zf-C2H2"]; Sp2[gene ID: "ENSMUSG00000018678" species: "Mus musculus" TF status: "inferred" TF family: "C2H2 ZF" DBDs: "zf-C2H2"]; Sp3[gene ID: "ENSMUSG00000027109" species: "Mus musculus" TF status: "inferred" TF family: "C2H2 ZF" DBDs: "zf-C2H2"]; Sp5[gene ID: "ENSMUSG00000075304" species: "Mus musculus" TF status: "inferred" TF family: "C2H2 ZF" DBDs: "zf-C2H2"]; Sp6[gene ID: "ENSMUSG00000038560" species: "Mus musculus" TF status: "inferred" TF family: "C2H2 ZF" DBDs: "zf-C2H2"]; Sp7[gene ID: "ENSMUSG00000060284" species: "Mus musculus" TF status: "inferred" TF family: "C2H2 ZF" DBDs: "zf-C2H2"]; Sp8[gene ID: "ENSMUSG00000048562" species: "Mus musculus" TF status: "inferred" TF family: "C2H2 ZF" DBDs: "zf-C2H2"]; Sp9[gene ID: "ENSMUSG00000068859" species: "Mus musculus" TF status: "inferred" TF family: "C2H2 ZF" DBDs: "zf-C2H2"]<br>Possible TFs: SP3	*3.24003*	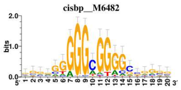	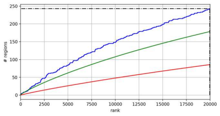	link	link	PWMs
351	☐ homer__CGGTGACGTCAC_CRE<br>Description: CRE(bZIP)/Promoter/Homer	*3.23866*	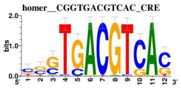	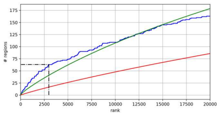	link	link	PWMs
352	☐ transfac_public__M00039<br>Description: V$CREB_01: CREB<br>Possible TFs: CREB1	*3.23806*	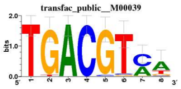	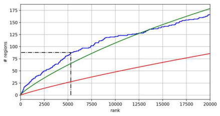	link	link	PWMs
353	☐ dbcorrdb__POLR2A__ENCSR000EBG_1__m1<br>Description: POLR2A (ENCSR000EBG-1, motif 1)<br>Possible TFs: POLR2A	*3.23577*	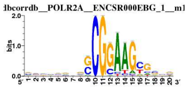	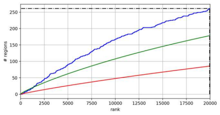	link	link	PWMs
354	☐ dbcorrdb__POLR2A__ENCSR000DKM_1__m2<br>Description: POLR2A (ENCSR000DKM-1, motif 2)<br>Possible TFs: POLR2A	*3.23486*	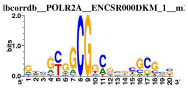	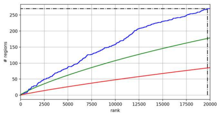	link	link	PWMs
355	☐ transfac_pro__M05051<br>Description: P$DREB1A_02: DREB1A	*3.23309*	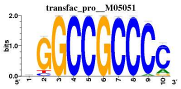	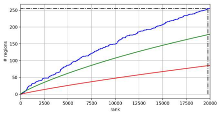	link	link	PWMs
356	☐ dbcorrdb__YY1__ENCSR000EWF_1__m2<br>Description: YY1 (ENCSR000EWF-1, motif 2)<br>Possible TFs: YY1	*3.23242*	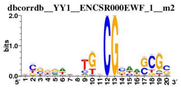	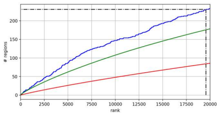	link	link	PWMs
357	☐ scertf__macisaac.URC2<br>Description: macisaac.URC2	*3.23119*	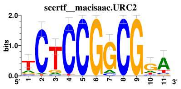	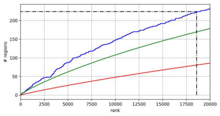	link	link	PWMs

#	Feature	NES	Logo	Recovery Curve	Candidate targets	All regions in top 20000	Database
358	☐ transfac_pro__M04710<br>Description: V$CHD2_01: CHD2<br>Possible TFs: CHD2	3.22947	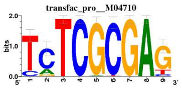	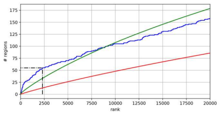	link	link	PWMs
359	☐ swissregulon__sacCer__CAT8<br>Description: sacCer__CAT8	3.22548	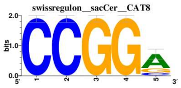	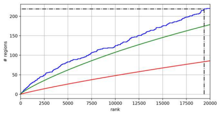	link	link	PWMs
360	☐ taipale_cyt_meth__CREB1_NRTGAYGTN_eDBD_meth<br>Description: CREB1 [bZIP, CpG-meth]<br>Possible TFs: CREB1	3.22426	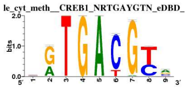	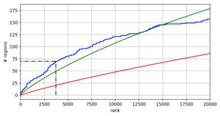	link	link	PWMs
361	☐ cisbp__M1543<br>Description: M1543	3.22277	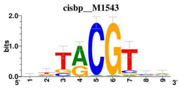	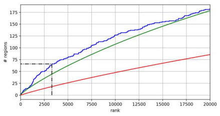	link	link	PWMs
362	☐ hocomoco__E4F1_HUMAN.H11MO.0.D<br>Description: E4F1_HUMAN<br>Possible TFs: E4F1	3.21903	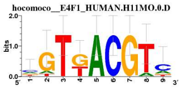	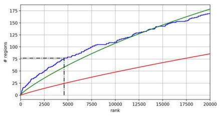	link	link	PWMs
363	☐ swissregulon__hs__ATF4.p2<br>Description: hs__ATF4.p2<br>Possible TFs: ATF4	3.21847	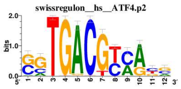	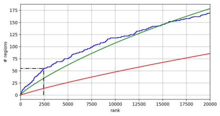	link	link	PWMs
364	☐ dbcorrdb__PHF8__ENCSR000AQH_1__m1<br>Description: PHF8 (ENCSR000AQH-1, motif 1)<br>Possible TFs: PHF8	3.21429	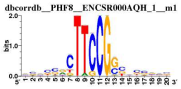	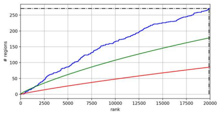	link	link	PWMs
365	☐ transfac_pro__M09077<br>Description: P$ERF6_03: ERF6	3.21129	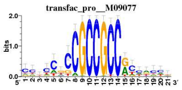	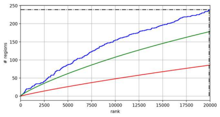	link	link	PWMs
366	☐<br>taipale_cyt_meth__ZNF454_TRGCGCCGGCGCYN_eDBD_meth<br>Description: ZNF454 [KRAB_Znf_C2H2, CpG-meth]<br>Possible TFs: ZNF454	3.21077	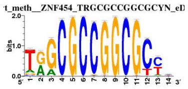	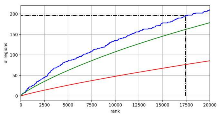	link	link	PWMs
367	☐ dbcorrdb__POLR2A__ENCSR000FAW_1__m1<br>Description: POLR2A (ENCSR000FAW-1, motif 1)<br>Possible TFs: POLR2A	3.21040	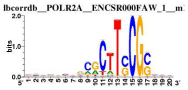	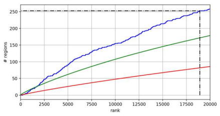	link	link	PWMs
368	☐ hocomoco__KLF3_HUMAN.H11MO.0.B<br>Description: KLF3_HUMAN<br>Possible TFs: KLF3	3.20981	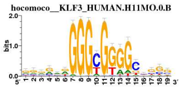	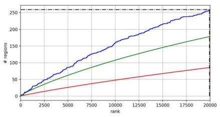	link	link	PWMs
369	☐ transfac_pro__M05139<br>Description: P$AT5G07310_01: At5g07310	3.20773	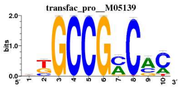	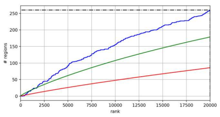	link	link	PWMs
370	☐ cisbp__M0319<br>Description: CrebB-17A[gene ID: "FBgn0014467" species: "Drosophila melanogaster" TF status: "inferred" TF family: "bZIP" DBDs: "bZIP_1"]; Crem[gene ID: "ENSMUSG00000063889" species: "Mus musculus" TF status: "direct" TF family: "bZIP" DBDs: "bZIP_1"]<br>Possible TFs: CREM	3.20745	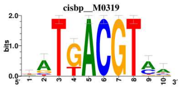	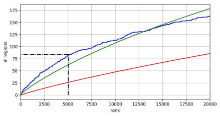	link	link	PWMs

#	Feature	NES	Logo	Recovery Curve	Candidate targets	All regions in top 20000	Database
371	☐ ENCFF001SOX<br>Description: DNase-seq on embryonic human myoblast (HSMM)	*3.20717*		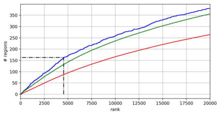	link	link	DHS & FAIRE
372	☐ dbcorrdb__POLR2A__ENCSR000BJM_1__m1<br>Description: POLR2A (ENCSR000BJM-1, motif 1)<br>Possible TFs: POLR2A	*3.20603*	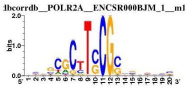	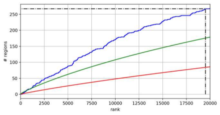	link	link	PWMs
373	☐ transfac_pro__M04869<br>Description: V$EGR1_11: Egr-1<br>Possible TFs: EGR1	*3.20383*	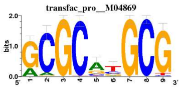	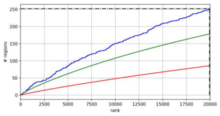	link	link	PWMs
374	☐ flyfactorsurvey__CG3065_F1-3_SANGER_2.5_FBgn0034946<br>Description: CG3065_F1-3_SANGER_2.5_FBgn0034946<br>Possible TFs: KLF18, KLF17	*3.20371*	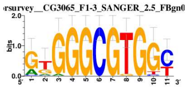	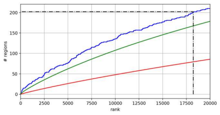	link	link	PWMs
375	☐ transfac_pro__M02091<br>Description: V$E4F1_Q6_01: E4F1<br>Possible TFs: E4F1	*3.20336*	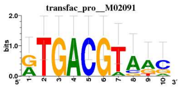	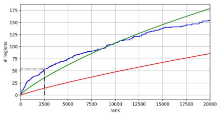	link	link	PWMs
376	☐ transfac_pro__M05099<br>Description: P$AT5G11190_01: At5g11190	*3.20267*	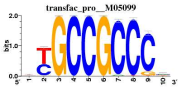	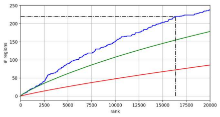	link	link	PWMs
377	☐ dbcorrdb__ZMIZ1__ENCSR000EFQ_1__m1<br>Description: ZMIZ1 (ENCSR000EFQ-1, motif 1)<br>Possible TFs: ZMIZ1	*3.20251*	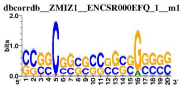	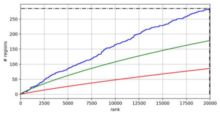	link	link	PWMs
378	☐ cisbp__M1548<br>Description: M1548	*3.20153*	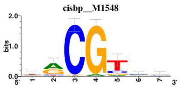	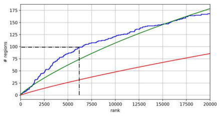	link	link	PWMs
379	☐ jaspar__MA0976.1<br>Description: CRF4	*3.20073*	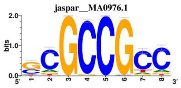	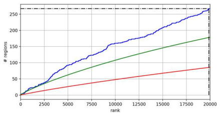	link	link	PWMs
380	☐ elemento__CCGCCGC<br>Description: Conserved regulatory element CCGCCGC between Hs and Mm	*3.20059*	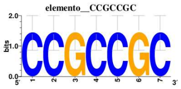	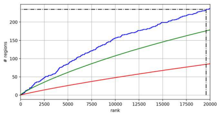	link	link	PWMs

#	Feature	NES	Logo	Recovery Curve	Candidate targets	All regions in top 20000	Database
381	☐ cisbp__M0401<br>Description: SP5[gene ID: "ENSG00000204335" species: "Homo sapiens" TF status: "inferred" TF family: "C2H2 ZF" DBDs: "zf-C2H2"]; SP6[gene ID: "ENSG00000189120" species: "Homo sapiens" TF status: "inferred" TF family: "C2H2 ZF" DBDs: "zf-C2H2"]; SP7[gene ID: "ENSG00000170374" species: "Homo sapiens" TF status: "inferred" TF family: "C2H2 ZF" DBDs: "zf-C2H2"]; SP9[gene ID: "ENSG00000217236" species: "Homo sapiens" TF status: "inferred" TF family: "C2H2 ZF" DBDs: "zf-C2H2"]; Sp2[gene ID: "ENSMUSG00000018678" species: "Mus musculus" TF status: "inferred" TF family: "C2H2 ZF" DBDs: "zf-C2H2"]; Sp3[gene ID: "ENSMUSG00000027109" species: "Mus musculus" TF status: "inferred" TF family: "C2H2 ZF" DBDs: "zf-C2H2"]; Sp4[gene ID: "ENSMUSG00000025323" species: "Mus musculus" TF status: "direct" TF family: "C2H2 ZF" DBDs: "zf-C2H2"]; Sp5[gene ID: "ENSMUSG00000075304" species: "Mus musculus" TF status: "inferred" TF family: "C2H2 ZF" DBDs: "zf-C2H2"]; Sp6[gene ID: "ENSMUSG00000038560" species: "Mus musculus" TF status: "inferred" TF family: "C2H2 ZF" DBDs: "zf-C2H2"]; Sp7[gene ID: "ENSMUSG00000060284" species: "Mus musculus" TF status: "inferred" TF family: "C2H2 ZF" DBDs: "zf-C2H2"]; Sp8[gene ID: "ENSMUSG00000048562" species: "Mus musculus" TF status: "inferred" TF family: "C2H2 ZF" DBDs: "zf-C2H2"]; Sp9[gene ID: "ENSMUSG00000068859" species: "Mus musculus" TF status: "inferred" TF family: "C2H2 ZF" DBDs: "zf-C2H2"]<br>Possible TFs: SP4	*3.19772*	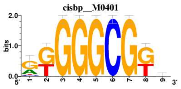	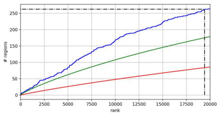	**link**	**link**	PWMs
382	☐ transfac_pro__M05044<br>Description: P$ERF1B_03: ERF1B	*3.19548*	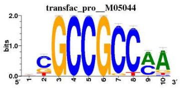	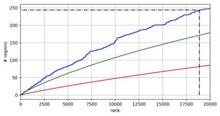	**link**	**link**	PWMs
383	☐ jaspar__MA0375.1<br>Description: RSC30	*3.19548*	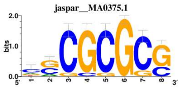	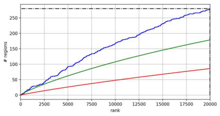	**link**	**link**	PWMs
384	☐ transfac_pro__M01899<br>Description: F$RSC30_02: Rsc30p	*3.19505*	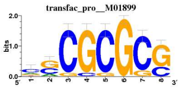	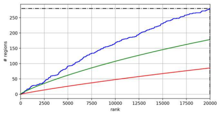	**link**	**link**	PWMs
385	☐ cisbp__M4838<br>Description: CG3065[gene ID: "FBgn0034946" species: "Drosophila melanogaster" TF status: "direct" TF family: "C2H2 ZF" DBDs: "zf-C2H2"]<br>Possible TFs: KLF18, KLF17	*3.19339*	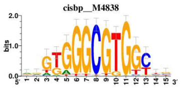	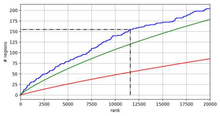	**link**	**link**	PWMs
386	☐ cisbp__M3090<br>Description: CREB1[gene ID: "ENSG00000118260" species: "Homo sapiens" TF status: "direct" TF family: "bZIP" DBDs: "bZIP_1"]; CrebB-17A[gene ID: "FBgn0014467" species: "Drosophila melanogaster" TF status: "inferred" TF family: "bZIP" DBDs: "bZIP_1"]<br>Possible TFs: CREB1	*3.19196*	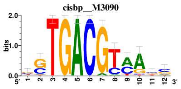	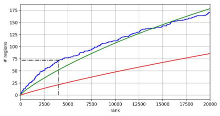	**link**	**link**	PWMs
387	☐ homer__CTGCGCATGCGC_NRF1<br>Description: NRF1(NRF)/MCF7-NRF1-ChIP-Seq(Unpublished)/Homer<br>Possible TFs: NRF1	*3.19057*	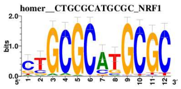	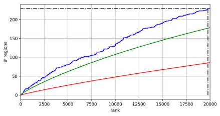	**link**	**link**	PWMs

#	Feature	NES	Logo	Recovery Curve	Candidate targets	All regions in top 20000	Database
388	☐ transfac_pro__M09026<br>Description: P$AT1G28160_02: AT1G28160	3.19012	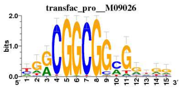	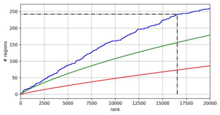	link	link	PWMs
389	☐ transfac_pro__M09578<br>Description: P$RAP23_05: RAP2-3	3.18935	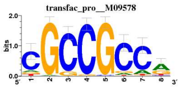	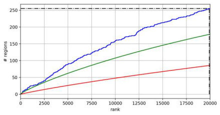	link	link	PWMs
390	☐ cisbp__M0265<br>Description: M0265	3.18616	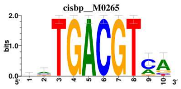	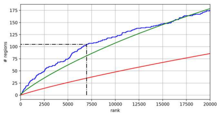	link	link	PWMs
391	☐ transfac_pro__M04703<br>Description: V$ETS1_05: c-ets-1<br>Possible TFs: ETS1	3.18561	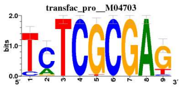	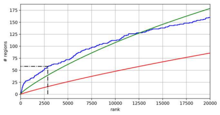	link	link	PWMs
392	☐ dbcorrdb__ELF1__ENCSR000BPT_1__m2<br>Description: ELF1 (ENCSR000BPT-1, motif 2)<br>Possible TFs: ELF1	3.18553	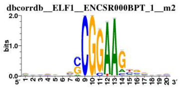	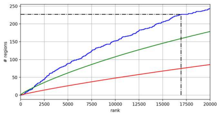	link	link	PWMs
393	☐ transfac_pro__M01253<br>Description: V$CNOT3_01: CNOT3<br>Possible TFs: CNOT3	3.18516	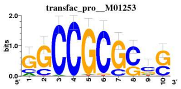	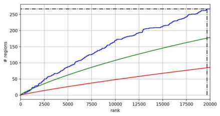	link	link	PWMs
394	☐ transfac_pro__M05026<br>Description: P$DREB1A_01: DREB1A	3.18448	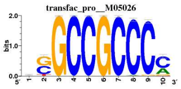	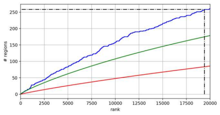	link	link	PWMs
395	☐ cisbp__M4473<br>Description: AL662830.5[gene ID: "ENSG00000237344" species: "Homo sapiens" TF status: "inferred" TF family: "Homeodomain" DBDs: "Homeobox"]; AL845464.3[gene ID: "ENSG00000206315" species: "Homo sapiens" TF status: "inferred" TF family: "Homeodomain" DBDs: "Homeobox"]; BX284686.1[gene ID: "ENSG00000224952" species: "Homo sapiens" TF status: "inferred" TF family: "Homeodomain" DBDs: "Homeobox"]; BX927239.6[gene ID: "ENSG00000232005" species: "Homo sapiens" TF status: "inferred" TF family: "Homeodomain" DBDs: "Homeobox"]; CR812478.2[gene ID: "ENSG00000225987" species: "Homo sapiens" TF status: "inferred" TF family: "Homeodomain" DBDs: "Homeobox"]; CR933878.8[gene ID: "ENSG00000236353" species: "Homo sapiens" TF status: "inferred" TF family: "Homeodomain" DBDs: "Homeobox"]; PBX3[gene ID: "ENSG00000167081" species: "Homo sapiens" TF status: "direct" TF family: "Homeodomain" DBDs: "Homeobox"]; Pbx2[gene ID: "ENSMUSG00000034673" species: "Mus musculus" TF status: "inferred" TF family: "Homeodomain" DBDs: "Homeobox"]; Pbx3[gene ID: "ENSMUSG00000038718" species: "Mus musculus" TF status: "inferred" TF family: "Homeodomain" DBDs: "Homeobox"]; Pbx4[gene ID: "ENSMUSG00000031860" species: "Mus musculus" TF status: "inferred" TF family: "Homeodomain" DBDs: "Homeobox"]<br>Possible TFs: PBX3	3.18159	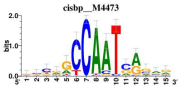	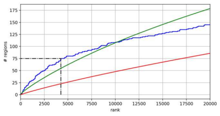	link	link	PWMs

#	Feature	NES	Logo	Recovery Curve	Candidate targets	All regions in top 20000	Database
396	☐ transfac_pro__M00371<br>Description: P$CPRF2_Q2: CPRF-2	3.18024	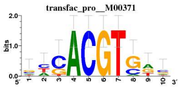	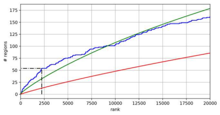	link	link	PWMs
397	☐ cisbp__M1546<br>Description: M1546	3.17644	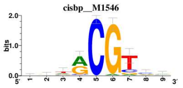	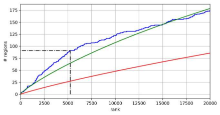	link	link	PWMs
398	☐ jaspar__MA0280.1<br>Description: CAT8	3.17556	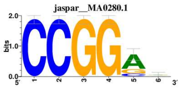	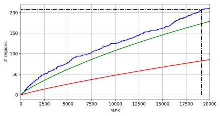	link	link	PWMs
399	☐ transfac_pro__M02810<br>Description: V$SP4_03: Sp4<br>Possible TFs: SP4	3.17501	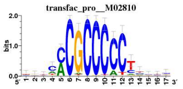	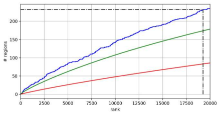	link	link	PWMs
400	☐ dbcorrdb__POLR2A__ENCSR000EFB_1__m3<br>Description: POLR2A (ENCSR000EFB-1, motif 3)<br>Possible TFs: POLR2A	3.17485	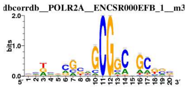	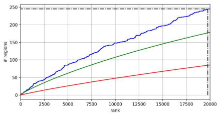	link	link	PWMs
401	☐ transfac_pro__M07248<br>Description: V$CREB1_Q3: CREB1<br>Possible TFs: CREB1	3.17128	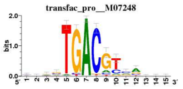	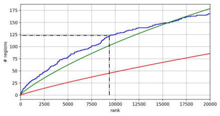	link	link	PWMs
402	☐ cisbp__M6152<br>Description: ATF1[gene ID: "ENSG00000123268" species: "Homo sapiens" TF status: "direct" TF family: "bZIP" DBDs: "bZIP_1"]; CrebB-17A[gene ID: "FBgn0014467" species: "Drosophila melanogaster" TF status: "inferred" TF family: "bZIP" DBDs: "bZIP_1"]<br>Possible TFs: ATF1	3.17091	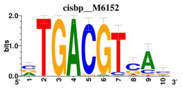	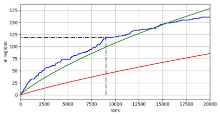	link	link	PWMs
403	☐ cisbp__M2506<br>Description: M2506	3.17064	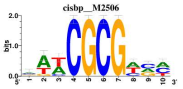	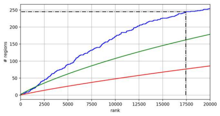	link	link	PWMs
404	☐ dbcorrdb__POLR2A__ENCSR000EXX_1__m1<br>Description: POLR2A (ENCSR000EXX-1, motif 1)<br>Possible TFs: POLR2A	3.17052	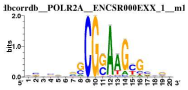	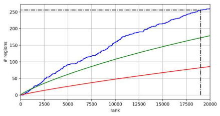	link	link	PWMs
405	☐ cisbp__M2323<br>Description: ZBTB33[gene ID: "ENSG00000177485" species: "Homo sapiens" TF status: "direct" TF family: "C2H2 ZF" DBDs: "zf-C2H2"]<br>Possible TFs: ZBTB33	3.16704	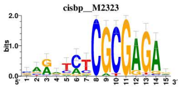	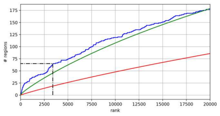	link	link	PWMs
406	☐ dbcorrdb__ELK4__ENCSR000EVI_1__m1<br>Description: ELK4 (ENCSR000EVI-1, motif 1)<br>Possible TFs: ELK4	3.16581	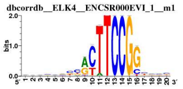	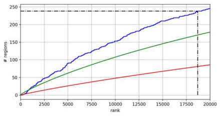	link	link	PWMs
407	☐ taipale_cyt_meth__KLF14_NRCCACGCCCMYN_eDBD<br>Description: KLF14 [Znf_C2H2]<br>Possible TFs: KLF14	3.16560	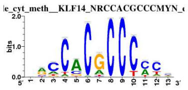	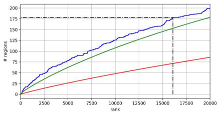	link	link	PWMs

#	Feature	NES	Logo	Recovery Curve	Candidate targets	All regions in top 20000	Database
408	☐ elemento__CGCCGCC<br>Description: Conserved regulatory element CGCCGCC between Dmel and Dpse; Conserved regulatory element CGCCGCC between Hs and Mm	*3.16192*	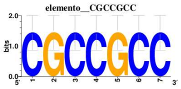	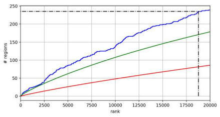	**link**	**link**	PWMs
409	☐ swissregulon__sacCer__RDS1<br>Description: sacCer__RDS1	*3.15898*	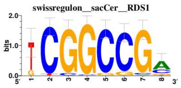	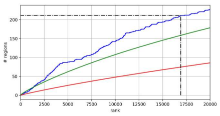	**link**	**link**	PWMs
410	☐ transfac_pro__M04973<br>Description: P$PTI5_01: Pti5	*3.15801*	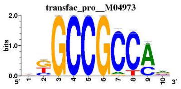	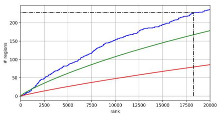	**link**	**link**	PWMs
411	☐ hdpi__SMAP1L<br>Description: SMAP1L<br>Possible TFs: SMAP2	*3.15581*	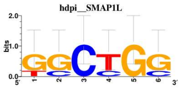	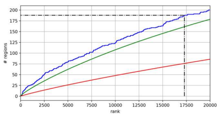	**link**	**link**	PWMs
412	☐ jaspar__MA0079.3<br>Description: SP1<br>Possible TFs: SP1	*3.15574*	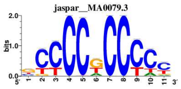	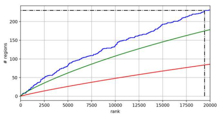	**link**	**link**	PWMs
413	☐ taipale_cyt_meth__CREB1_NRTGACGTCAYN_eDBD<br>Description: CREB1 [bZIP]<br>Possible TFs: CREB1	*3.15502*	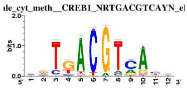	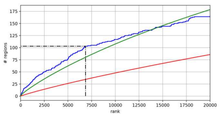	**link**	**link**	PWMs
414	☐ dbcorrdb__ATF1__ENCSR000DNZ_1__m2<br>Description: ATF1 (ENCSR000DNZ-1, motif 2)<br>Possible TFs: ATF1	*3.15422*	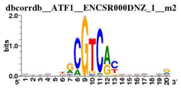	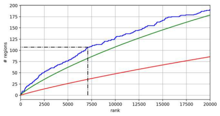	**link**	**link**	PWMs
415	☐ dbcorrdb__HA-E2F1__ENCSR000EVM_1__m1<br>Description: HA-E2F1 (ENCSR000EVM-1, motif 1)<br>Possible TFs: E2F1	*3.15412*	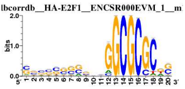	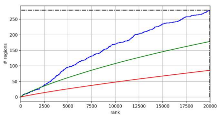	**link**	**link**	PWMs
416	☐ transfac_pro__M05014<br>Description: P$AT5G25390_01: At5g25390	*3.15215*	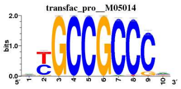	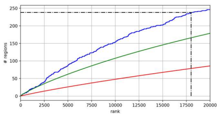	**link**	**link**	PWMs
417	☐ yetfasco__YML076C_325<br>Description: YML076C_325	*3.15144*	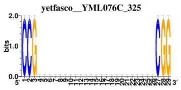	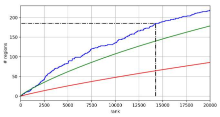	**link**	**link**	PWMs
418	☐ fantom__motif65_TNGCGA<br>Description: DENOVO_SCANALL_HUMAN: motif_TNGCGA	*3.14857*	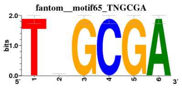	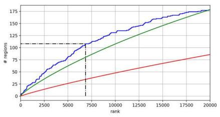	**link**	**link**	PWMs
419	☐ dbcorrdb__POLR2A__ENCSR000EZL_1__m1<br>Description: POLR2A (ENCSR000EZL-1, motif 1)<br>Possible TFs: POLR2A	*3.14739*	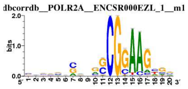	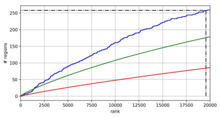	**link**	**link**	PWMs
420	☐ transfac_pro__M09087<br>Description: P$PUCHI_01: PUCHI	*3.14128*	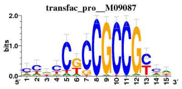	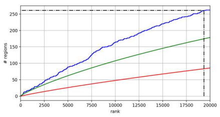	**link**	**link**	PWMs

#	Feature	NES	Logo	Recovery Curve	Candidate targets	All regions in top 20000	Database
421	☐ E126-H4K20me1-broadpeak<br>Description: H4K20me1 in NHDF-Ad Adult Dermal Fibroblast Primary Cells (E126, broadpeak)	3.14108		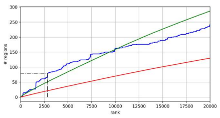	link	link	Histone modifications
422	☐ transfac_pro__M09053<br>Description: P$AT1G68550_02: AT1G68550	3.14012	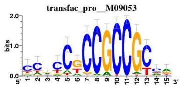	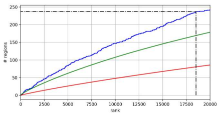	link	link	PWMs
423	☐ cisbp__M1806<br>Description: CHA4[gene ID: "YLR098C" species: "Saccharomyces cerevisiae" TF status: "direct" TF family: "Zinc cluster" DBDs: "Zn_clus"]	3.13987	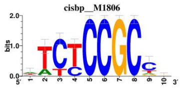	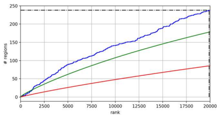	link	link	PWMs
424	☐ cisbp__M1547<br>Description: M1547	3.13904	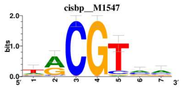	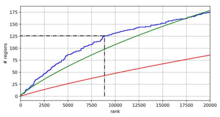	link	link	PWMs
425	☐ dbcorrdb__POLR2A__ENCSR000EBC_1__m1<br>Description: POLR2A (ENCSR000EBC-1, motif 1)<br>Possible TFs: POLR2A	3.13498	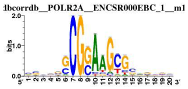	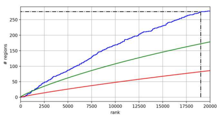	link	link	PWMs
426	☐ transfac_pro__M05144<br>Description: P$AT1G49120_01: At1g49120	3.12744	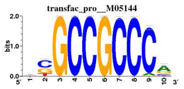	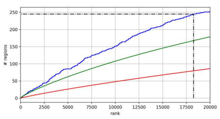	link	link	PWMs
427	☐ transfac_pro__M07514<br>Description: P$RRTF1_02: RRTF1	3.12631	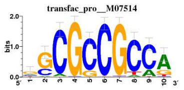	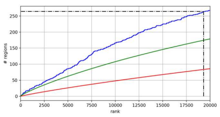	link	link	PWMs
428	☐ dbcorrdb__POLR2A__ENCSR000DKM_1__m1<br>Description: POLR2A (ENCSR000DKM-1, motif 1)<br>Possible TFs: POLR2A	3.12339	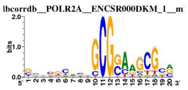	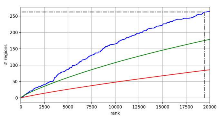	link	link	PWMs
429	☐ ENCFF001VZY<br>Description: DNase-seq on human HFF	3.12272		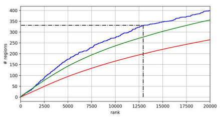	link	link	DHS & FAIRE
430	☐ cisbp__M5593<br>Description: KLF16[gene ID: "ENSG00000129911" species: "Homo sapiens" TF status: "direct" TF family: "C2H2 ZF" DBDs: "zf-C2H2"]; Klf11[gene ID: "ENSMUSG00000020653" species: "Mus musculus" TF status: "inferred" TF family: "C2H2 ZF" DBDs: "zf-C2H2"]; Klf13[gene ID: "ENSMUSG00000052040" species: "Mus musculus" TF status: "inferred" TF family: "C2H2 ZF" DBDs: "zf-C2H2"]; Klf14[gene ID: "ENSMUSG00000073209" species: "Mus musculus" TF status: "inferred" TF family: "C2H2 ZF" DBDs: "zf-C2H2"]<br>Possible TFs: KLF16	3.12183	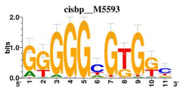	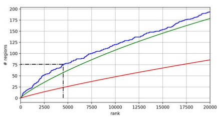	link	link	PWMs
431	☐ scertf__badis.RSC30<br>Description: badis.RSC30	3.11860	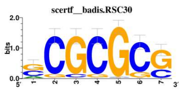	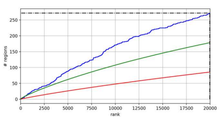	link	link	PWMs
432	☐ dbcorrdb__POLR2A__ENCSR000FAL_1__m1<br>Description: POLR2A (ENCSR000FAL-1, motif 1)<br>Possible TFs: POLR2A	3.11503	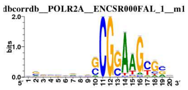	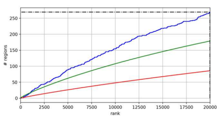	link	link	PWMs

#	Feature	NES	Logo	Recovery Curve	Candidate targets	All regions in top 20000	Database
433	☐ cisbp__M4552<br>Description: ATF3[gene ID: "ENSG00000162772" species: "Homo sapiens" TF status: "direct" TF family: "bZIP" DBDs: "bZIP_1"]<br>Possible TFs: ATF3	*3.11489*	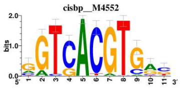	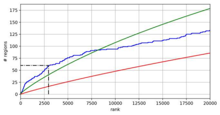	link	link	PWMs
434	☐ dbcorrdb__POLR2A__ENCSR000BMR_1__m1<br>Description: POLR2A (ENCSR000BMR-1, motif 1)<br>Possible TFs: POLR2A	*3.11307*	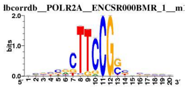	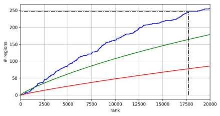	link	link	PWMs
435	☐ dbcorrdb__ZBTB33__ENCSR000BNY_1__m1<br>Description: ZBTB33 (ENCSR000BNY-1, motif 1)<br>Possible TFs: ZBTB33	*3.10969*	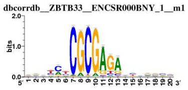	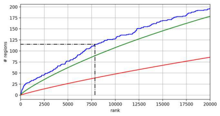	link	link	PWMs
436	☐ taipale__KLF16_DBD_GCCMCGCCCMC_repr<br>Description: KLF16 monomeric C2H2 DBD<br>Possible TFs: KLF16	*3.10954*	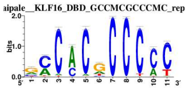	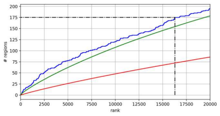	link	link	PWMs
437	☐ transfac_pro__M09082<br>Description: P$ESE1_01: ESE1	*3.10925*	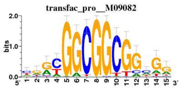	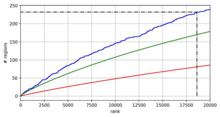	link	link	PWMs
438	☐ dbcorrdb__E2F6__ENCSR000EWJ_1__m2<br>Description: E2F6 (ENCSR000EWJ-1, motif 2)<br>Possible TFs: E2F6	*3.10892*	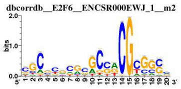	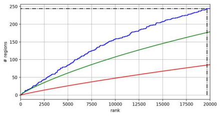	link	link	PWMs
439	☐ elemento__ATTGGCCG<br>Description: Conserved regulatory element ATTGGCCG between Hs and Mm	*3.10587*	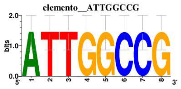	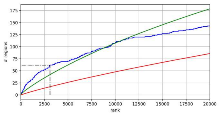	link	link	PWMs
440	☐ cisbp__M0033<br>Description: M0033	*3.10496*	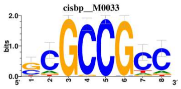	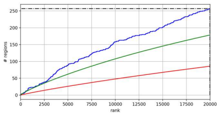	link	link	PWMs
441	☐ cisbp__M1550<br>Description: Gmeb1[gene ID: "ENSMUSG00000028901" species: "Mus musculus" TF status: "direct" TF family: "SAND" DBDs: "SAND"]<br>Possible TFs: GMEB1	*3.10369*	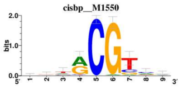	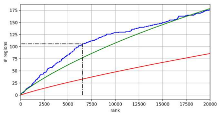	link	link	PWMs
442	☐ elemento__GCCGCGC<br>Description: Conserved regulatory element GCCGCGC between Hs and Mm	*3.10239*	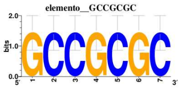	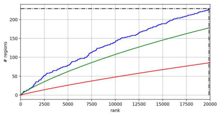	link	link	PWMs
443	☐ dbcorrdb__POLR2A__ENCSR000EAM_1__m1<br>Description: POLR2A (ENCSR000EAM-1, motif 1)<br>Possible TFs: POLR2A	*3.10154*	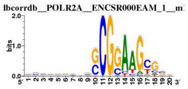	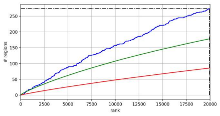	link	link	PWMs
444	☐ dbcorrdb__UBTF__ENCSR000EFW_1__m1<br>Description: UBTF (ENCSR000EFW-1, motif 1)<br>Possible TFs: UBTF	*3.09961*	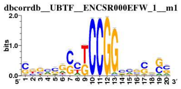	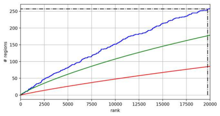	link	link	PWMs
445	☐ hocomoco__ATF1_MOUSE.H11MO.0.B<br>Description: ATF1_MOUSE<br>Possible TFs: ATF1	*3.09426*	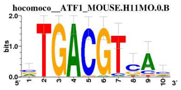	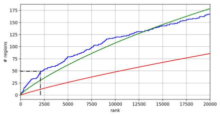	link	link	PWMs

#	Feature	NES	Logo	Recovery Curve	Candidate targets	All regions in top 20000	Database
446	☐ dbcorrdb__ATF3__ENCSR000BKC_1__m1<br>Description: ATF3 (ENCSR000BKC-1, motif 1)<br>Possible TFs: ATF3	3.09390	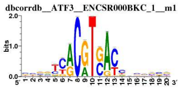	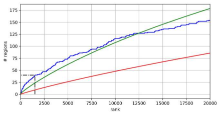	link	link	PWMs
447	☐ transfac_pro__M04990<br>Description: P$DREB2B_01: DREB2B	3.09303	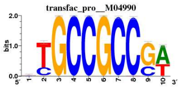	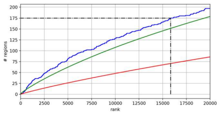	link	link	PWMs
448	☐ dbcorrdb__POLR2A__ENCSR000EZA_1__m1<br>Description: POLR2A (ENCSR000EZA-1, motif 1)<br>Possible TFs: POLR2A	3.09013	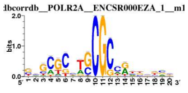	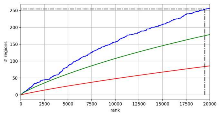	link	link	PWMs
449	☐ scertf__badis.CAT8<br>Description: badis.CAT8	3.08977	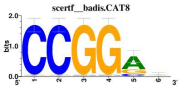	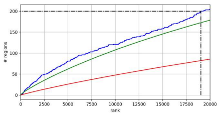	link	link	PWMs
450	☐ elemento__AGCGGCG<br>Description: Conserved regulatory element AGCGGCG between Hs and Mm	3.08837	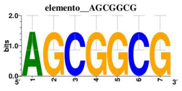	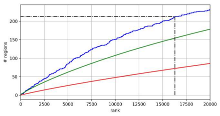	link	link	PWMs
451	☐ dbcorrdb__ZBTB33__ENCSR000BPZ_1__m1<br>Description: ZBTB33 (ENCSR000BPZ-1, motif 1)<br>Possible TFs: ZBTB33	3.08713	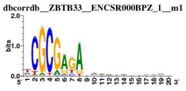	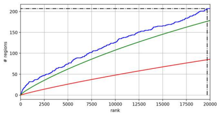	link	link	PWMs
452	☐ E007-DNase.hotspot.all.peaks-narrowpeak<br>Description: DNase in H1 Derived Neuronal Progenitor Cultured Cells (E007, narrowpeak, hotspot)	3.08655		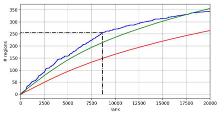	link	link	DHS & FAIRE
453	☐ cisbp__M0076<br>Description: M0076	3.08487	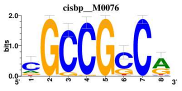	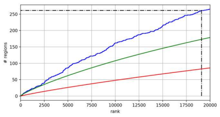	link	link	PWMs
454	☐ transfac_pro__M09080<br>Description: P$ERF8_02: ERF8	3.08443	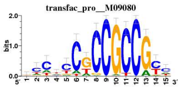	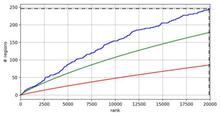	link	link	PWMs
455	☐ elemento__CGCCTCC<br>Description: Conserved regulatory element CGCCTCC between Hs and Mm	3.08369	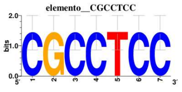	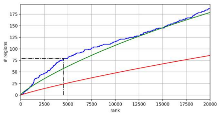	link	link	PWMs
456	☐ elemento__TCGCCGC<br>Description: Conserved regulatory element TCGCCGC between Hs and Mm	3.08013	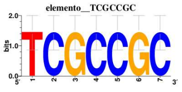	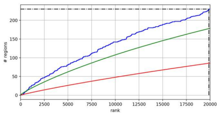	link	link	PWMs
457	☐ yetfasco__YIL036W_585<br>Description: YIL036W_585	3.07995	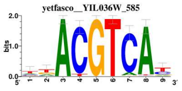	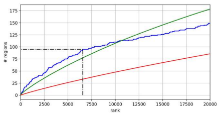	link	link	PWMs
458	☐ transfac_pro__M01615<br>Description: F$CST6_01: Cst6p	3.07837	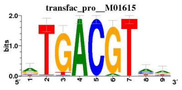	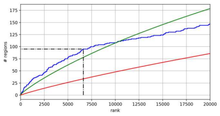	link	link	PWMs

#	Feature	NES	Logo	Recovery Curve	Candidate targets	All regions in top 20000	Database
459	☐ transfac_pro__M00917<br>Description: V$CREB_Q4_01: CREB<br>Possible TFs: CREM, CREB1	3.07830	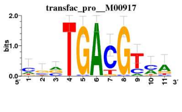	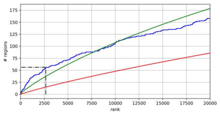	link	link	PWMs
460	☐ hocomoco__KAISO_HUMAN.H11MO.0.A<br>Description: KAISO_HUMAN<br>Possible TFs: ZBTB33	3.07530	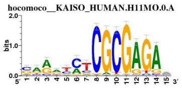	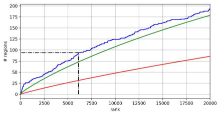	link	link	PWMs
461	☐ transfac_public__M00178<br>Description: V$CREB_Q4: CREB<br>Possible TFs: CREB1	3.07420	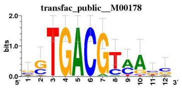	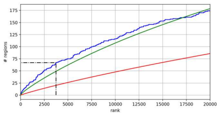	link	link	PWMs
462	☐ transfac_pro__M04683<br>Description: V$NFYA_02: NF-YA<br>Possible TFs: NFYA	3.07241	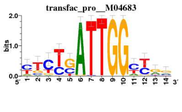	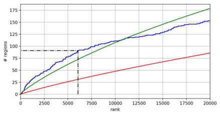	link	link	PWMs
463	☐ jaspar__MA0286.1<br>Description: CST6	3.07231	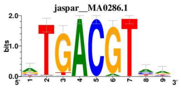	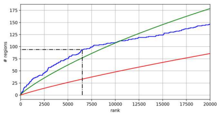	link	link	PWMs
464	☐ cisbp__M2091<br>Description: CST6[gene ID: "YIL036W" species: "Saccharomyces cerevisiae" TF status: "direct" TF family: "bZIP" DBDs: "bZIP_1"]	3.07036	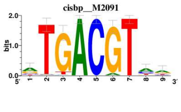	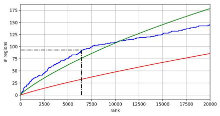	link	link	PWMs
465	☐ transfac_pro__M04790<br>Description: V$E2F6_02: E2F-6<br>Possible TFs: E2F6	3.06981	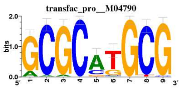	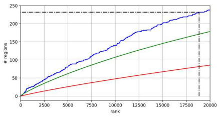	link	link	PWMs
466	☐ jaspar__MA0506.1<br>Description: NRF1<br>Possible TFs: NRF1	3.06886	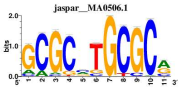	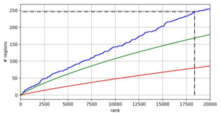	link	link	PWMs
467	☐ cisbp__M4307<br>Description: CST6[gene ID: "YIL036W" species: "Saccharomyces cerevisiae" TF status: "direct" TF family: "bZIP" DBDs: "bZIP_1"]	3.06684	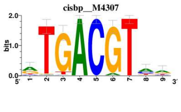	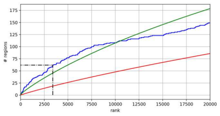	link	link	PWMs
468	☐ yetfasco__YLR278C_2112<br>Description: YLR278C_2112	3.06484	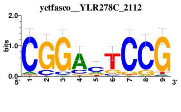	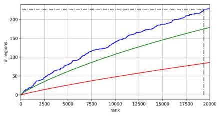	link	link	PWMs
469	☐ transfac_pro__M01273<br>Description: V$SP4_Q5: SP4<br>Possible TFs: SP4	3.06476	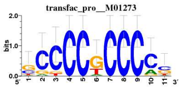	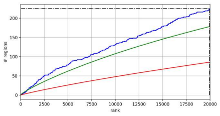	link	link	PWMs
470	☐ transfac_pro__M05353<br>Description: V$KLF14_02: Klf14<br>Possible TFs: KLF14	3.06250	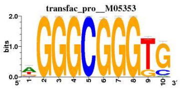	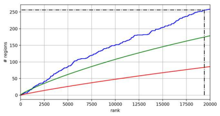	link	link	PWMs
471	☐ jaspar__MA1005.1<br>Description: ERF3	3.06130	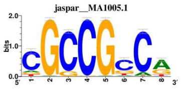	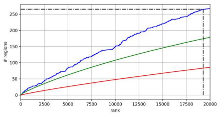	link	link	PWMs

#	Feature	NES	Logo	Recovery Curve	Candidate targets	All regions in top 20000	Database
472	☐ transfac_pro__M05140<br>Description: P$AT4G28140_01: At4g28140	*3.05962*	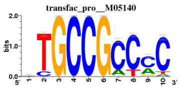	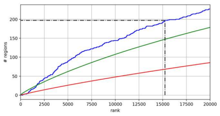	**link**	**link**	PWMs
473	☐<br>taipale_cyt_meth__ZNF704_NRCCGGCCGGYN_FL_meth_repr<br>Description: ZNF704 [Znf_C2H2, CpG-meth]<br>Possible TFs: ZNF704	*3.05919*	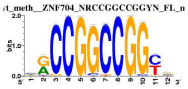	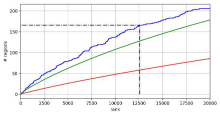	**link**	**link**	PWMs
474	☐ transfac_pro__M05013<br>Description: P$ERF4_02: ERF4	*3.05501*	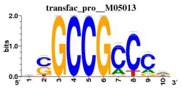	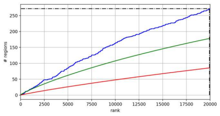	**link**	**link**	PWMs
475	☐ transfac_pro__M09084<br>Description: P$LEP_02: LEP	*3.05465*	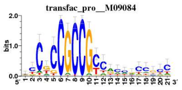	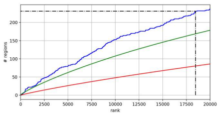	**link**	**link**	PWMs
476	☐ dbcorrdb__TAF1__ENCSR000BJN_1__m1<br>Description: TAF1 (ENCSR000BJN-1, motif 1)<br>Possible TFs: TAF1	*3.05429*	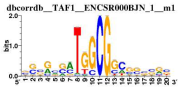	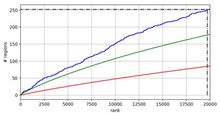	**link**	**link**	PWMs
477	☐ elemento__CGCGCCG<br>Description: Conserved regulatory element CGCGCCG between Hs and Mm	*3.05031*	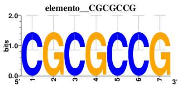	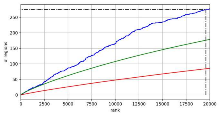	**link**	**link**	PWMs
478	☐ dbcorrdb__POLR2A__ENCSR000DYO_1__m2<br>Description: POLR2A (ENCSR000DYO-1, motif 2)<br>Possible TFs: POLR2A	*3.04638*	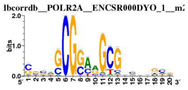	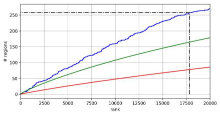	**link**	**link**	PWMs
479	☐ elemento__AGCGCGC<br>Description: Conserved regulatory element AGCGCGC between Dmel and Dpse; Conserved regulatory element AGCGCGC between Hs and Mm	*3.04351*	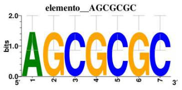	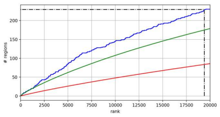	**link**	**link**	PWMs
480	☐ ENCFF001SPN<br>Description: Duke human ProgFib DNase-seq	*3.04020*		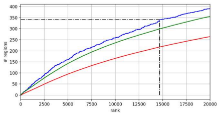	**link**	**link**	DHS & FAIRE
481	☐ transfac_public__M00179<br>Description: V$CREBP1_Q2: ATF2<br>Possible TFs: ATF2	*3.03964*	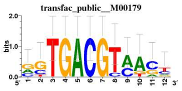	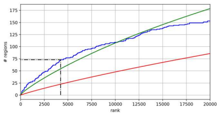	**link**	**link**	PWMs
482	☐ swissregulon__sacCer__GAL4<br>Description: sacCer__GAL4	*3.03930*	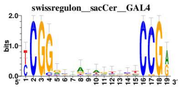	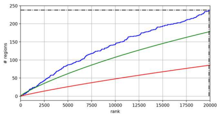	**link**	**link**	PWMs

#	Feature	NES	Logo	Recovery Curve	Candidate targets	All regions in top 20000	Database
483	☐ cisbp__M1906<br>Description: SP1[gene ID: "ENSG00000185591" species: "Homo sapiens" TF status: "direct" TF family: "C2H2 ZF" DBDs: "zf-C2H2"]; SP5[gene ID: "ENSG00000204335" species: "Homo sapiens" TF status: "inferred" TF family: "C2H2 ZF" DBDs: "zf-C2H2"]; SP6[gene ID: "ENSG00000189120" species: "Homo sapiens" TF status: "inferred" TF family: "C2H2 ZF" DBDs: "zf-C2H2"]; SP7[gene ID: "ENSG00000170374" species: "Homo sapiens" TF status: "inferred" TF family: "C2H2 ZF" DBDs: "zf-C2H2"]; SP9[gene ID: "ENSG00000217236" species: "Homo sapiens" TF status: "inferred" TF family: "C2H2 ZF" DBDs: "zf-C2H2"]; Sp2[gene ID: "ENSMUSG00000018678" species: "Mus musculus" TF status: "inferred" TF family: "C2H2 ZF" DBDs: "zf-C2H2"]; Sp3[gene ID: "ENSMUSG00000027109" species: "Mus musculus" TF status: "inferred" TF family: "C2H2 ZF" DBDs: "zf-C2H2"]; Sp5[gene ID: "ENSMUSG00000075304" species: "Mus musculus" TF status: "inferred" TF family: "C2H2 ZF" DBDs: "zf-C2H2"]; Sp6[gene ID: "ENSMUSG00000038560" species: "Mus musculus" TF status: "inferred" TF family: "C2H2 ZF" DBDs: "zf-C2H2"]; Sp7[gene ID: "ENSMUSG00000060284" species: "Mus musculus" TF status: "inferred" TF family: "C2H2 ZF" DBDs: "zf-C2H2"]; Sp8[gene ID: "ENSMUSG00000048562" species: "Mus musculus" TF status: "inferred" TF family: "C2H2 ZF" DBDs: "zf-C2H2"]; Sp9[gene ID: "ENSMUSG00000068859" species: "Mus musculus" TF status: "inferred" TF family: "C2H2 ZF" DBDs: "zf-C2H2"]<br>Possible TFs: SP1	*3.03838*	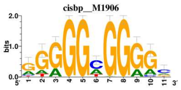	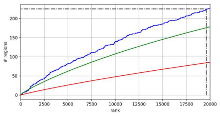	**link**	**link**	PWMs
484	☐ cisbp__M6373<br>Description: NFYC[gene ID: "ENSG00000066136" species: "Homo sapiens" TF status: "direct" TF family: "Unknown" DBDs: "UNKNOWN"]<br>Possible TFs: NFYC	*3.03721*	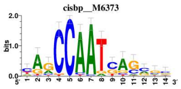	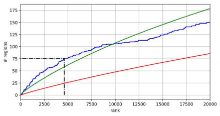	**link**	**link**	PWMs
485	☐ dbcorrdb__SIN3A__ENCSR000BOW_1__m3<br>Description: SIN3A (ENCSR000BOW-1, motif 3)<br>Possible TFs: SIN3A	*3.03696*	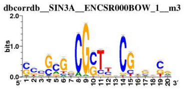	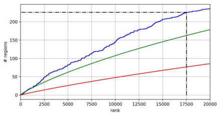	**link**	**link**	PWMs
486	☐ transfac_pro__M05041<br>Description: P$ERF4_03: ERF4	*3.03591*	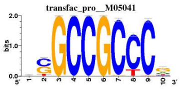	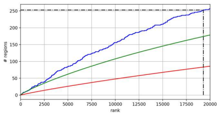	**link**	**link**	PWMs
487	☐ scertf__macisaac.SWI6<br>Description: macisaac.SWI6	*3.03570*	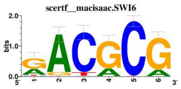	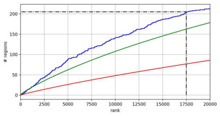	**link**	**link**	PWMs
488	☐ dbcorrdb__SREBF1__ENCSR000EZP_1__m1<br>Description: SREBF1 (ENCSR000EZP-1, motif 1)<br>Possible TFs: SREBF1	*3.03429*	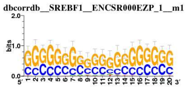	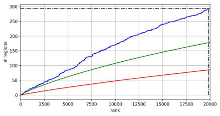	**link**	**link**	PWMs
489	☐ E089-DNase.hotspot.all.peaks-narrowpeak<br>Description: DNase in Fetal Muscle Trunk (E089, narrowpeak, hotspot)	*3.03338*		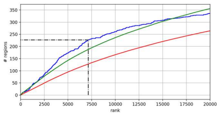	**link**	**link**	DHS & FAIRE
490	☐ dbcorrdb__MAZ__ENCSR000ECL_1__m1<br>Description: MAZ (ENCSR000ECL-1, motif 1)<br>Possible TFs: MAZ	*3.02946*	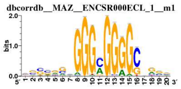	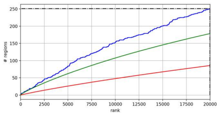	**link**	**link**	PWMs

#	Feature	NES	Logo	Recovery Curve	Candidate targets	All regions in top 20000	Database
491	☐ hdpi__AFF4<br>Description: AFF4<br>Possible TFs: AFF4	*3.02824*	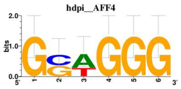	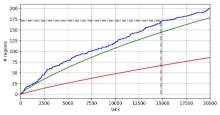	link	link	PWMs
492	☐ hocomoco__CREB1_HUMAN.H11MO.0.A<br>Description: CREB1_HUMAN<br>Possible TFs: CREB1	*3.02683*	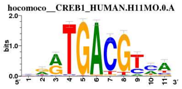	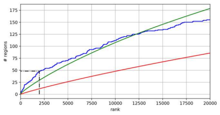	link	link	PWMs
493	☐ taipale_cyt_meth__ATF2_NRTGAYGTMAYN_FL<br>Description: ATF2 [bZIP]<br>Possible TFs: ATF2	*3.02622*	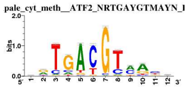	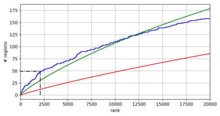	link	link	PWMs
494	☐ transfac_pro__M05111<br>Description: P$AT1G44830_01: At1g44830	*3.02425*	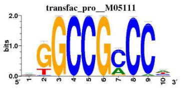	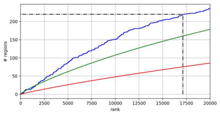	link	link	PWMs
495	☐ transfac_pro__M05479<br>Description: V$ZNF580_01: znf580<br>Possible TFs: ZNF580	*3.02305*	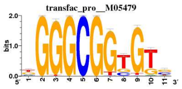	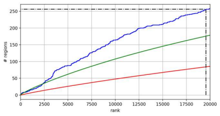	link	link	PWMs
496	☐ transfac_pro__M01520<br>Description: F$RSC30_01: Rsc30p	*3.02278*	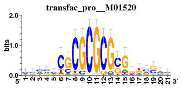	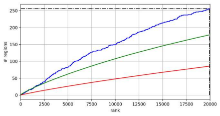	link	link	PWMs
497	☐ dbcorrdb__POLR2AphosphoS5__ENCSR000BIC_1__m1<br>Description: POLR2AphosphoS5 (ENCSR000BIC-1, motif 1)	*3.02276*	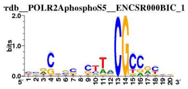	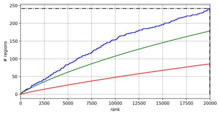	link	link	PWMs
498	☐ scertf__badis.GSM1<br>Description: badis.GSM1	*3.02034*	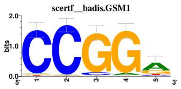	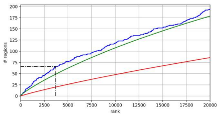	link	link	PWMs
499	☐ dbcorrdb__POLR2A__ENCSR000EAY_1__m1<br>Description: POLR2A (ENCSR000EAY-1, motif 1)<br>Possible TFs: POLR2A	*3.01969*	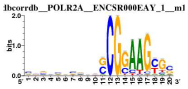	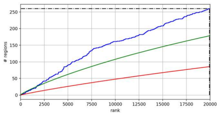	link	link	PWMs
500	☐ E080-DNase.hotspot.all.peaks-narrowpeak<br>Description: DNase in Fetal Adrenal Gland (E080, narrowpeak, hotspot)	*3.01960*		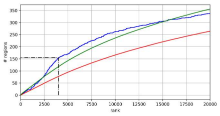	link	link	DHS & FAIRE
501	☐ taipale_cyt_meth__ZNF704_NRCCGGCCGGYN_FL<br>Description: ZNF704 [Znf_C2H2]<br>Possible TFs: ZNF704	*3.01876*	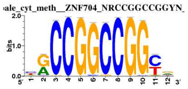	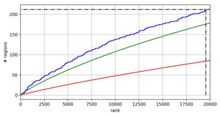	link	link	PWMs
502	☐ taipale_cyt_meth__CREB3L4_NRTGACGTCAYN_eDBD<br>Description: CREB3L4 [bZIP]<br>Possible TFs: CREB3L4	*3.01815*	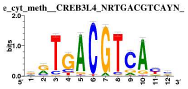	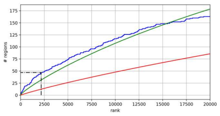	link	link	PWMs
503	☐ cisbp__M4300<br>Description: RSC30[gene ID: "YHR056C" species: "Saccharomyces cerevisiae" TF status: "direct" TF family: "Zinc cluster" DBDs: "Zn_clus"]	*3.01772*	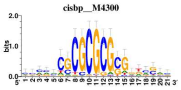	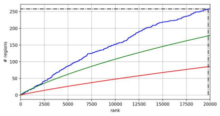	link	link	PWMs

#	Feature	NES	Logo	Recovery Curve	Candidate targets	All regions in top 20000	Database
504	☐ transfac_pro__M05146<br>Description: P$LEP_01: LEP	*3.01483*	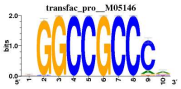	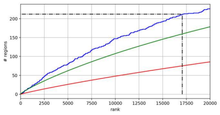	link	link	PWMs
505	☐ transfac_pro__M01721<br>Description: V$PUR1_Q4: PUR1<br>Possible TFs: PURA	*3.01192*	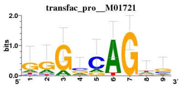	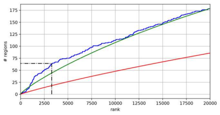	link	link	PWMs
506	☐ jaspar__MA0470.1<br>Description: E2F4<br>Possible TFs: E2F4	*3.00875*	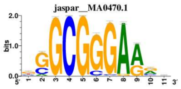	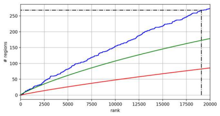	link	link	PWMs
507	☐ transfac_pro__M09079<br>Description: P$ERF73_01: ERF73	*3.00673*	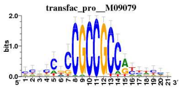	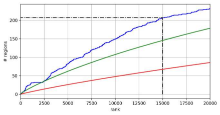	link	link	PWMs
508	☐ ENCFF001SPI<br>Description: Duke human Myometr DNase-seq	*3.00645*		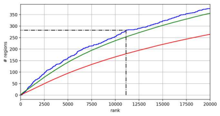	link	link	DHS & FAIRE
509	☐ dbcorrdb__POLR2AphosphoS5__ENCSR000BML_1__m1<br>Description: POLR2AphosphoS5 (ENCSR000BML-1, motif 1)	*3.00629*	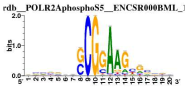	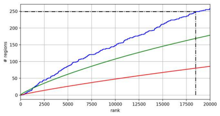	link	link	PWMs
510	☐ transfac_pro__M09065<br>Description: P$ERF104_02: ERF104	*3.00567*	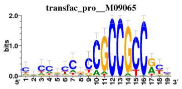	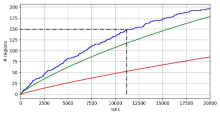	link	link	PWMs
511	☐ cisbp__M5853<br>Description: SP1[gene ID: "ENSG00000185591" species: "Homo sapiens" TF status: "direct" TF family: "C2H2 ZF" DBDs: "zf-C2H2"]; SP5[gene ID: "ENSG00000204335" species: "Homo sapiens" TF status: "inferred" TF family: "C2H2 ZF" DBDs: "zf-C2H2"]; SP6[gene ID: "ENSG00000189120" species: "Homo sapiens" TF status: "inferred" TF family: "C2H2 ZF" DBDs: "zf-C2H2"]; SP7[gene ID: "ENSG00000170374" species: "Homo sapiens" TF status: "inferred" TF family: "C2H2 ZF" DBDs: "zf-C2H2"]; SP9[gene ID: "ENSG00000217236" species: "Homo sapiens" TF status: "inferred" TF family: "C2H2 ZF" DBDs: "zf-C2H2"]; Sp2[gene ID: "ENSMUSG00000018678" species: "Mus musculus" TF status: "inferred" TF family: "C2H2 ZF" DBDs: "zf-C2H2"]; Sp3[gene ID: "ENSMUSG00000027109" species: "Mus musculus" TF status: "inferred" TF family: "C2H2 ZF" DBDs: "zf-C2H2"]; Sp5[gene ID: "ENSMUSG00000075304" species: "Mus musculus" TF status: "inferred" TF family: "C2H2 ZF" DBDs: "zf-C2H2"]; Sp6[gene ID: "ENSMUSG00000038560" species: "Mus musculus" TF status: "inferred" TF family: "C2H2 ZF" DBDs: "zf-C2H2"]; Sp7[gene ID: "ENSMUSG00000060284" species: "Mus musculus" TF status: "inferred" TF family: "C2H2 ZF" DBDs: "zf-C2H2"]; Sp8[gene ID: "ENSMUSG00000048562" species: "Mus musculus" TF status: "inferred" TF family: "C2H2 ZF" DBDs: "zf-C2H2"]; Sp9[gene ID: "ENSMUSG00000068859" species: "Mus musculus" TF status: "inferred" TF family: "C2H2 ZF" DBDs: "zf-C2H2"]<br>Possible TFs: SP1	*3.00307*	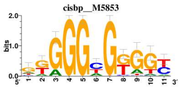	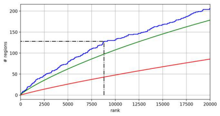	link	link	PWMs

#	Feature	NES	Logo	Recovery Curve	Candidate targets	All regions in top 20000	Database
512	☐ transfac_pro__M05048<br>Description: P$RAP26_02: RAP2-6	*3.00293*	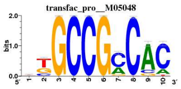	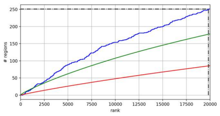	**link**	**link**	PWMs

© 2015-2019 **KULeuven** | Authors: Gert Hulselmans & Hana Imrichova | Web design (CSS) by **Andreas Viklund.**
