# Supplementary figures and images for: Characterization of Accessible Chromatin Regions in Cattle Rumen Epithelial Tissue during Weaning
Source: Genes (Basel). 2022 Mar 18;13(3):535. doi: 10.3390/genes13030535 (PMC8949786; doi:10.3390/genes13030535)

Weaning regions over chromosomes

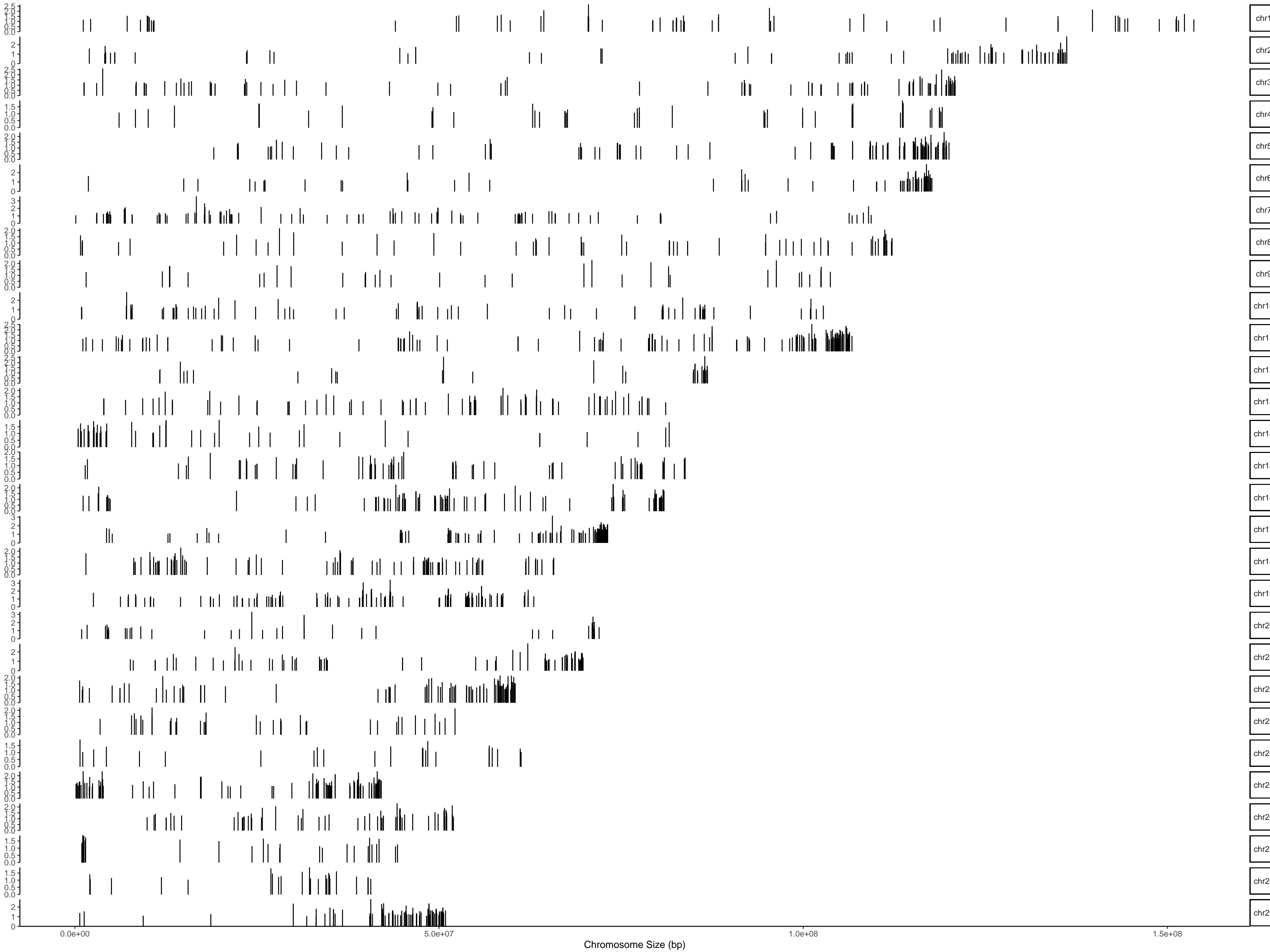

Supplement: Supplementary file 1 [file genes-13-00535-s001.zip › Figure S3.pdf]

# Cluster Dendrogram

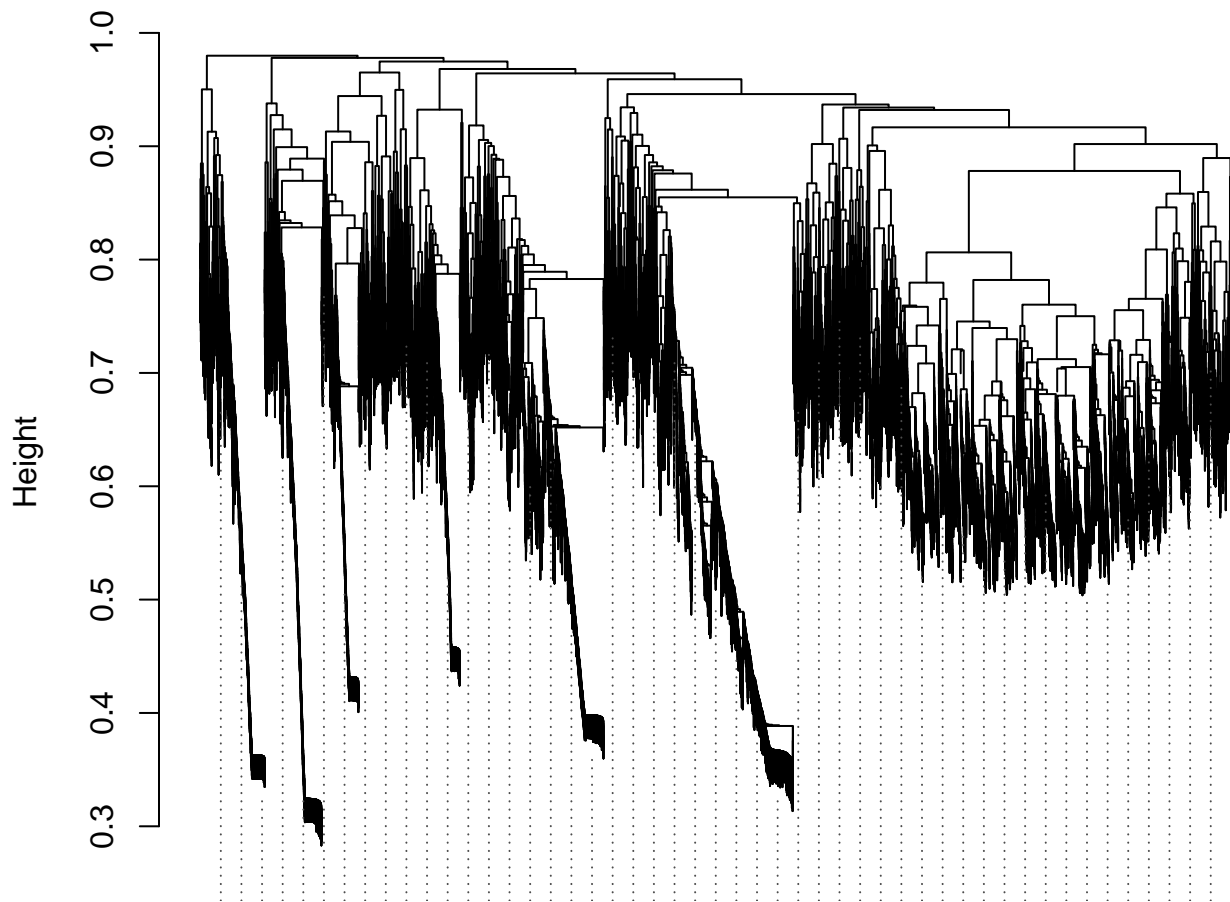

Dynamic Tree Cut

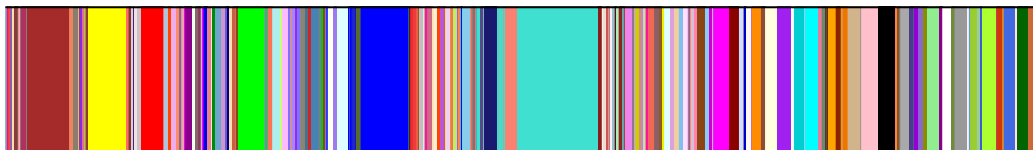

Merged dynamic

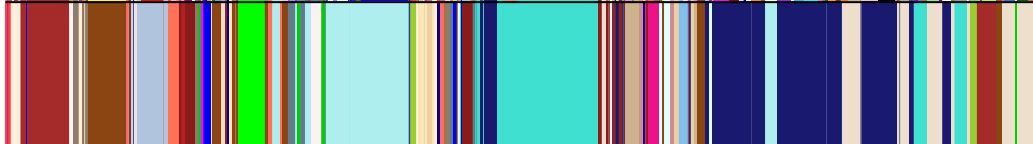

Supplement: Supplementary file 1 [file genes-13-00535-s001.zip › Figure S5.pdf]
